# Supplementary material for: Thiocoumarin Caged Nucleotides: Synthetic Access and Their Photophysical Properties
Source: Molecules. 2020 Nov 15;25(22):5325. doi: 10.3390/molecules25225325 (PMC7696096; doi:10.3390/molecules25225325)
Supplement: Supplementary file 1 [file molecules-25-05325-s001.pdf]

Thio-coumarin Caged Nucleotides: Synthetic Access and Their Photophysical Properties

Jiahui Ma [1], Alexander Ripp [1,2], Daniel Wassy [1], Tobias Dürr [1], Danye Qiu [1], Markus Häner [1], Thomas M. Haas [1], Christoph Popp [1], Dominik Bezold [1], Sabine Richert [3], Birgit Esser [1,2] and Henning J. Jessen [1,2,*]

[1] Institute of Organic Chemistry, University of Freiburg, Albertstr. 21, 79104 Freiburg, Germany
[2] Cluster of Excellence livMatS @ FIT − Freiburg Center for Interactive Materials and Bioinspired Technologies, University of Freiburg, Georges-Köhler-Allee 105, 79110 Freiburg, Germany
[3] Institute of Physical Chemistry, University of Freiburg, Albertstr. 21, 79104 Freiburg, Germany

Supplementary Information

Table of Contents

1. Abbreviations....2
2. General Remarks....3
3. Synthesis of the compounds....5
  3.1 Synthesis of photocage: Thio-DEACM....5
  3.2 Synthesis of photocaged nucleotides....8
4. Spectroscopic Characterization....15
  4.1 Absorption and Fluorescence Spectra....15
  4.2 Molar absorption coefficient....18
  4.3 Fluorescence quantum yield....18
  4.4 DFT Calculations....19
  4.5 Calculated Structures....20
5. Photolysis....23
  5.1 Aqueous stability of thio-DEACM caged compounds....23
  5.2 Experimental details....23
  5.3 Uncaging of thio-DEACM caged ATP at 490 nm....25
  5.4 Uncaging of thio-DEACM caged ADP at 490 nm....27
  5.5 Uncaging of thio-DEACM AP_4 at 490 nm....29
  5.6 Uncaging of DEACM caged ADP, ATP, AP_4 at 490 nm....31
  5.7 Uncaging of DEACM caged ATP and thio-DEACM caged ATP at 400 nm....32
  5.8 Explanation of absence of thio-DEACM in HPLC analysis....34
6. NMR spectra of the compounds....35
7. Reference....63

1. Abbreviations

DEACM : 7-dietehylamino-4-hydroxymethyl-coumarin

TBA : Tetrabutylammonium

ETT : 5-(Ethylthio)-1*H*-tetrazole

*m*CPBA : *meta*-Chloroperbenzoic acid

TEA : Triethylamine

Et_2O : Diethyl ether

MeCN : Acetonitrile

DMF : Dimethyl formamide

DBU : 1,8-Diazabicyclo[5.4.0]undec-7-ene

UV : ultra-violet

DCM: Dichloromethane

Rf: Retention factor

EtOAc: Ethyl acetate

Eq.: Equivalent

Thio-DEACM: 7-diethylamino-4-hydroxymethyl-thiocoumarin

Quant.: Quantitative yield

4-DMAP: 4-(dimethylamino)pyridine

Ar: Argon gas

TEAA: Triethylammonium acetate

TLC: Thin layer chromatography

2. General Remarks

Reagents were purchased from commercial suppliers (Acros, Merck, etc.) and used without further purification, unless noted otherwise. Solvents were obtained in analytical grade and used as received for reactions, extractions, chromatography and precipitation.

Nucleotides were obtained as sodium salts. The corresponding tetrabutylammonium (TBA) salts of these nucleotides were obtained by cation exchange, as described later on**.**

Thin Layer Chromatography was carried out using Merck silica gel 60 F254 plates, visualized with UV light.

Anion Exchange Chromatography was performed using Q Sepharose® Fast Flow or DEAE Sepharose® Fast Flow (Sigma). Crude products were loaded with water and eluted with stepwise increase of an aqueous ammonium bicarbonate solution (1 M) as an eluent.

Centrifugation was performed with a Hettich Universal 320.

Lyophilizations were done with Christ Freeze Dryer Alpha 1-4 LD+ and Zirbus Technology VaCo 5 freeze dryer.

Mass spectra were recorded by C. Warth (Mass spectrometry service of the University of Freiburg) on a Thermo LCQ Advantage [spray voltage: 2.5 – 4.0 kV, spray current: 5 µA, ion transfer tube: 250 (150) °C, evaporation temperature: 50 – 400 °C.

Preparative Reverse-Phase-MPLC (RP-MPLC) was performed with the Flash Chromatography System PuriFlash® 5.125 from Interchim® using an Interchim® PF-C18 AQ column.

Analytical RP-HPLC-MS was performed on an HPLC-MS from Thermo Scientific equipped with a Dionex UltiMate 3000 Pump, Dionex UltiMate 3000 Autosampler, Dionex UltiMate 3000 Colum Compartment, Dionex UltiMate 3000 Diode Array Detector, Dionex UltiMate 3000 Fluorescence Detector and MSQ Plus single-quadrupole mass spectrometer using an Isera ISAspher 100-3 C18 AQ, 150 × 3.0 mm column at a flow rate of 0.5 mL/min.

The ^{1}H-, ^{13}C-, ^{31}P-NMR spectra were measured on a Bruker Avance III HD 300 MHz (282 MHz for ^{19}F, 122 MHz for ^{31}P) and on a Bruker Avance Neo 400 MHz (101 MHz for ^{13}C, 377 MHz for ^{19}F, 162 MHz for ^{31}P) NMR spectrometer. All signals were referenced to an internal solvent signal (^{1}H-NMR: $CDCl_3$: δ = 7.29 ppm, D_2O: δ = 4.79 ppm; ^{13}C-NMR: $CDCl_3$: δ = 77.16 ppm). The signals of ^{19}F- and ^{31}P-NMR spectra were referenced to an external standard. The chemical shifts are quoted in ppm. The splitting patterns are labeled as: singlet (s), broad singlet (br s), doublet (d), triplet (t), quartett (q), septet (sep), multipet (m). The coupling constants J are given in Hertz (Hz). The evaluation of NMR-spectra was done using the software MestreNova from Mestrelab Research.

CE-ESI-MS analysis was performed on an Agilent 7100 capillary electrophoresis system, coupled to a Agilent 6520 Q-TOF with a commercial CE-MS adapter and sprayer kit by Agilent. A bare-fused silica capillary with a length of 100 cm (50 µm i.d. and 365 µm o.d.) was used. A 35 mM ammonium acetate buffer (pH 9.75) was used as a BGE. Water-isopropanol (1:1) spiked with mass references was used as a sheath liquid, applied at a constant flow rate of 1.5 µL/min. A constant CE current (23 µA) was used for analysis by

application of 30 kV over the capillary. Samples were analyzed in negative ESI ionization mode. The obtained data was analyzed by AgilentMassHunter Workstation.

3. Synthesis of the compounds

3.1 Synthesis of photocage: Thio-DEACM

Synthesis of **DEACM** was modified from a previously reported procedure [1](Timo Weinrich, Markus Granz, Christian Grunewald, Thomas F. Prisner and Michael W. Gobel. "Synthesis of a Cytidine Phosphoramidite with Protected Nitroxide Spin Label for EPR Experiments with RNA". *Eur. J. Org. Chem.* **2017**, 491–496.).

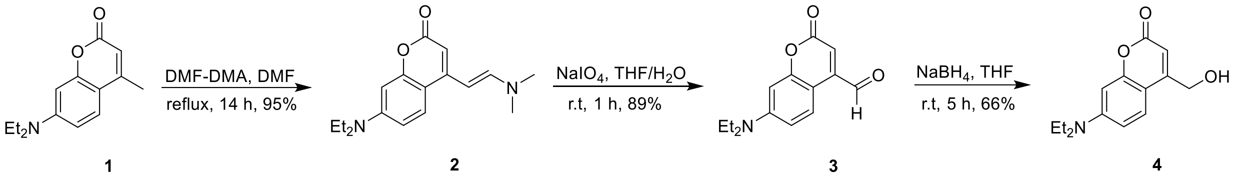

Scheme S1. Synthesis of DEACM **4**.

((*E*)-7-(Diethylamino)-4-[2-(dimethylamino)vinyl]-2*H*-chromen-2-one) (2)

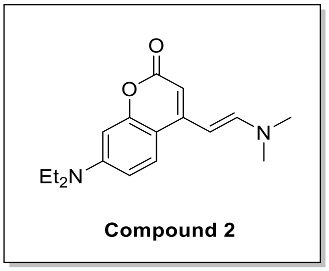

DMF–DMA (34.5 ml, 259.4 mmol, 2.0 eq.) was added to a solution of coumarin **1** (30.0 g, 129.7 mmol, 1.0 eq.) in DMF (200 ml) and it was then stirred under reflux for 14 h. After the reaction mixture cooled down to room temperature, it was slowly poured into ice water (400 ml). The formed yellow precipitate was filtered off and the filter cake was washed with water (5 x 300 ml). After the solid was dried at r.t. in an open beaker for 48 h, Compound 2 (35.23 g, 123.0 mmol, 95%) was obtained as a yellow solid and it can be used for the next step without further purification. Analytical data are consistent with those reported in the literature. Rf: 0.3 (DCM/EtOAc=4:1). **^{1}H NMR** (300 MHz, $CDCl_3$) δ 7.45 (d, J = 9.0 Hz, 1H), 7.14 (d, J = 13.0 Hz, 1H), 6.47 (dd, J = 9.0, 2.7 Hz, 1H), 6.41 (d, J = 2.6 Hz, 1H), 5.77 (s, 1H), 3.32 (q, J = 7.1 Hz, 4H), 2.91 (s, 6H), 1.12 (t, J = 7.1 Hz, 6H). **^{13}C NMR** (126 MHz, $CDCl_3$) δ 163.43, 156.40, 152.35, 150.14, 146.59, 124.87, 108.17, 107.92, 98.13, 93.47, 87.50, 44.68, 12.56.

(7-(Diethylamino)-2-oxo-2*H*-chromene-4-carbaldehyde) (3)

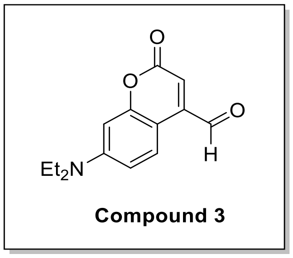

$NaIO_4$ (7.6 g, 35.7 mmol, 3.0 eq.) was added to a solution of enamine **2** (3.4 g, 11.9 mmol, 1.0 eq.) in THF/H_2O (80 ml 1:1) and the resulting mixture was stirred for 1 h at room temperature. The formed precipitate was filtered off and washed with EtOAc (3 x 20 ml). The filtrate was concentrated under reduced pressure (to evaporate the THF and EtOAc) and saturated aqueous $NaHCO_3$ solution was added. It was transferred into a separatory funnel, DCM (3 x 50ml) was used for extraction and the combined organic layers were dried over Na_2SO_4. The solvent was removed under reduced pressure and the residue was purified by silica gel chromatography (Cyclohexane/EtOAc, 10:1 to 5:1). Target compound **3** (2.6 g, 10.6 mmol, 89%) was obtained as a red solid. Analytical data are consistent with those reported in the literature. Rf: 0.3 (Cyclohexane/EtOAc=5:1). **^{1}H NMR** (300 MHz, $CDCl_3$) δ 9.96 (s, 1H), 8.23 (d, J = 9.2 Hz, 1H), 6.55 (dd, J = 9.2, 2.7 Hz, 1H), 6.45 (d, J = 2.6 Hz, 1H), 6.37 (s, 1H), 3.36 (q, J = 7.1 Hz, 4H), 1.15 (t, J = 7.1 Hz, 6H). **^{13}C NMR** (126 MHz, CDCl3) δ 192.54, 161.86, 157.42, 151.06, 143.93, 127.05, 117.34, 109.55, 103.74, 97.64, 44.84, 12.50.

(7-(Diethylamino)-4-(hydroxymethyl)-2*H*-chromen-2-one) (4)

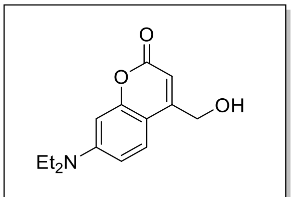

Compound 4

$NaBH_4$ (310 mg, 8.2 mmol, 2.0 eq.) was added to a 0 °C cold solution of aldehyde **3** (1.0 g, 4.1 mmol, 1.0 eq.) in THF (20 ml) and it was then stirred for 5 h at room temperature. Subsequently, saturated aqueous $NaHCO_3$ solution (40 ml) was added and extracted with DCM (3 x 40 ml) and the combined organic layers were dried with Na_2SO_4. The solvent was removed under reduced pressure and the residue was purified by silica gel chromatography (Cyclohexane/EtOAc, 8:1 to 1:1). Target compound **4** (680 mg, 2.7 mmol, 66%) was isolated as a light yellow solid. Analytical data are consistent with those reported in the literature. Rf: 0.2 (Cyclohexane/EtOAc=1:1). **^{1}H NMR** (300 MHz, $CDCl_3$) δ 7.33 (d, J = 9.0 Hz, 1H), 6.57 (dd, J = 9.0, 2.6 Hz, 1H), 6.48 (d, J = 2.6 Hz, 1H), 6.31 – 6.26 (m, 1H), 4.87 – 4.80 (d, 2H), 3.40 (q, J = 7.1 Hz, 4H), 3.07 – 2.94 (m, 1H), 1.20 (t, J = 7.1 Hz, 6H). **^{13}C NMR** (126 MHz, $CDCl_3$) δ 162.97, 156.12, 155.26, 150.55, 124.45, 108.68, 106.40, 105.32, 97.72, 60.90, 44.75, 12.50.

The synthesis of **Thio-DEACM** was modified from a previously reported procedure [2] (Ludovic Fournier, Carole Gauron, Lijun Xu.etc. “A Blue-Absorbing Photolabile Protecting Group for in Vivo Chromatically Orthogonal Photoactivation”. ACS Chem. Biol. 2013, 8, 1528−1536.).

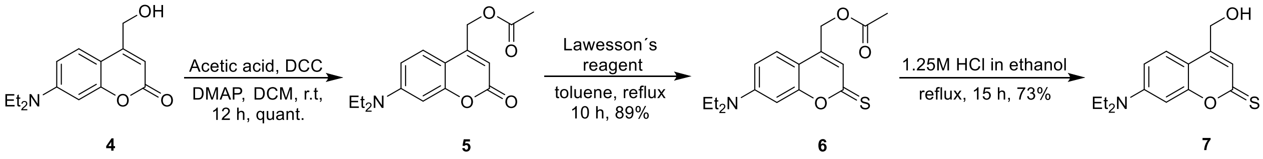

Scheme S2. Synthesis of thio-DEACM **7**.

(7-Diethylamino-4-methylacetoate-coumarin) (5)

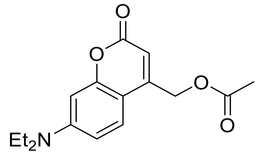

Compound 5

1,3-Dicyclohexylcarbodiimide (6.0 g, 29.2 mmol, 1.2 eq.) was added to a solution of **4** (6 g, 24.3 mmol, 1.0 eq.), acetic acid (1.7 ml, 29.2 mmol, 1.2 eq.) and 4-DMAP (3.6 g, 29.2 mmol, 1.2 eq.) in dry dichloromethane (100 ml) at 0 ºC under an atmosphere of argon. After 10 min at 0 ºC, the mixture was stirred at room temperature for 12 h in the dark. The formed precipitate was filtered off. The filtrate was washed with aqueous 1.0 M HCl, saturated aqueous $NaHCO_3$ solution and dried over Na_2SO_4. The solvent was evaporated under reduced pressure and the residue was purified by silica gel column chromatography (Cyclohexane/EtOAc, 10:1 to 5:1). Compound **5** (7 g, 24.2 mmol, quant.) was obtained as a yellow powder. Analytical data are in agreement with the literature. Rf: 0.2 (Cyclohexane/EtOAc=5:1). **^{1}H NMR** (300 MHz, $CDCl_3$) δ 7.21 (d, J = 9.1 Hz, 1H), 6.51 (dd, J = 9.0, 2.6 Hz, 1H), 6.44 (d, J = 2.6 Hz, 1H), 6.06 (d, J = 1.3 Hz, 1H), 5.14 (d, J = 1.3 Hz, 2H), 3.34 (q, J = 7.1 Hz, 4H), 2.12 (s, 3H), 1.14 (t, J = 7.1 Hz, 6H). **^{13}C NMR** (101 MHz, $CDCl_3$) δ 170.29, 161.91, 156.30, 150.71, 149.43, 124.41, 108.70, 106.46, 106.03, 97.88, 61.39, 44.80, 20.81, 12.48.

(7-Diethylamino-4-methylacetoate-thiocoumarin) (6)

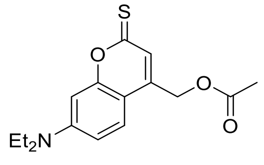

Compound 6

Lawesson's reagent (1.9 g, 4.7 mmol, 0.65 eq.) was added to a suspension of **5** (2.12 g, 7.3 mmol, 1.0 eq.) in dry toluene (100 ml) and it was stirred under reflux for 10 h, in the dark and under an atmosphere of argon. The solvent was removed under reduced pressure and the residue was purified by silica gel column chromatography (Cyclohexane/EtOAc, 10:1 to 6:1). Compound **6** (1.99 g, 6.5 mmol, 89%) was obtained as a yellow powder. Analytical data are consistent with those reported in the literature. Rf: 0.2 (Cyclohexane/EtOAc=5:1). **^{1}H NMR** (300 MHz, $CDCl_3$) δ 7.39 – 7.33 (d, 1H), 7.07 (s, 1H), 6.68 (m, 2H), 5.20 (d, J = 1.1 Hz, 2H), 3.45 (q, J = 7.1 Hz, 4H), 2.21 (s, 3H), 1.24 (t, J = 7.1 Hz, 6H). **^{13}C NMR** (101 MHz, $CDCl_3$) δ 197.19, 170.27, 159.04, 151.01, 141.84, 124.45, 120.56, 110.29, 108.18, 97.48, 60.97, 44.95, 20.79, 12.42.

(7-Diethylamino-4-hydroxymethyl-thiocoumarin) (7)

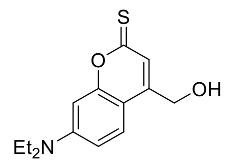

Compound 7

Ethanolic HCl solution (1.25 M, 36.6 ml, 45.8 mmol, 2.5 eq.) was added to a solution of **6** (5.6 g, 18.3 mmol, 1.0 eq.) in absolute ethanol (100 ml) and it was stirred under reflux for 6 h, in the dark and under argon atmosphere. After the addition of water (100 ml), the mixture was extracted with DCM (3 x 100 ml) and the combined organic layers were washed with saturated aqueous $NaHCO_3$ solution (3 x 100 ml), dried over Na_2SO_4. The solvent was removed under reduced pressure and the residue was purified by silica gel chromatography (Cyclohexane/EtOAc, 10:1 to 3:1). Compound **7** (3.5 g, 13.3 mmol, 73 %) was obtained as a yellow powder. Analytical data are consistent with those reported in the literature. Rf: 0.2 (Cyclohexane/EtOAc=3:1). **^{1}H NMR** (400 MHz, $CDCl_3$) δ 7.43 – 7.38 (d, 1H), 7.21 (t, *J* = 1.2 Hz, 1H), 6.75 – 6.59 (m, 2H), 4.83 (d, *J* = 5.1 Hz, 2H), 3.44 (q, *J* = 7.1 Hz, 4H), 1.23 (t, *J* = 7.1 Hz, 6H). **^{13}C NMR** (101 MHz, $CDCl_3$) δ 197.62, 158.99, 150.91, 147.36, 124.63, 119.78, 110.31, 108.45, 97.31, 60.64, 44.93 (2C), 12.44 (2C). **HRMS** (ESI): *m/z* for $C_{14}H_{17}NO_2S$, $[M+H]^+$ calcd: 264.1058; found : 264.1053. $[M-H]^-$ calcd : 262.0902; found : 262.0909

3.2 Synthesis of photocaged nucleotides

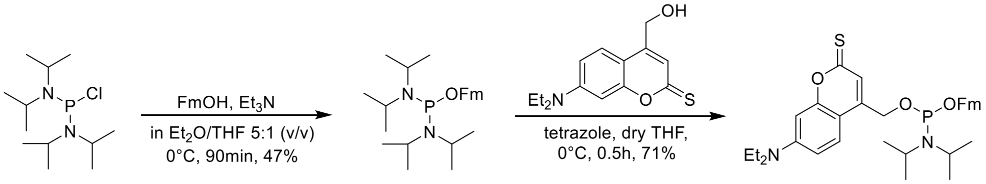

8

9

Scheme S3. Synthesis of P-amidite **9**.

((iPr$_2$N)$_2$P(OFm)) (8)

The synthesis of (iPr$_2$N) (FmO)P-OThioDEACM **(8)** **(9)** was adapted from a previously reported procedure [3] (Alexandre Hofer, Gregor S. Cremosnik, André C. Müller, Roberto Giambruno, Claudia Trefzer, Giulio Superti-Furga, Keiryn L. Bennett, and Henning J. Jessen*. “A Modular Synthesis of Modified Phosphoanhydrides”. Chem. Eur. J. 2015, 21, 10116 – 10122.)

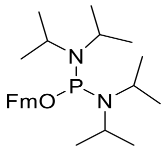

Compound 8

9-Fluorenylmethanol (6.03 g, 30.6 mmol, 1.02 eq.) was dried for 1 h under high vacuum. Afterwards, it was dissolved in dry Et_2O/THF 5:1 (v/v; 60 ml) under argon atmosphere, dry Et_3N (4.2 ml, 30.6 mmol, 1 eq.) was added and the mixture was cooled to 0 ºC. After the addition of bis(diisopropylamino)-chlorophosphine (8.16 g, 30.6 mmol, 1.00 eq.), it was stirred at 0 ºC for 1.5 h and the formed precipitate was quickly filtered off over neutral Al_2O_3. The filtrate was concentrated under reduced pressure and immediately purified by recrystallization from pentane 20 ml. (The product containing pentane was heated till all product dissolved in it, then filtered it through filter paper, the filtrate containing flask was kept in -20 ºC freezer for 5h, the crystal formed inside.) After filtration, the crystal was dried under reduced pressure (0.3 mbar) for 5h. Compound **8** (6.2 g, 14.5 mmol, 47%) was obtained as colorless, transparent crystals. Analytical data are consistent with those reported in the literature. **^{1}H NMR** (300 MHz, $CDCl_3$) δ 7.80 – 7.68 (m, 4H), 7.40 (td, *J* = 7.5, 1.2 Hz, 2H), 7.32 (td, *J* = 7.4, 1.2 Hz, 2H), 4.22 (t, *J* = 6.7 Hz, 1H), 3.92 (t, *J* = 6.8 Hz, 2H), 3.57 (sep, *J* = 10.7, 6.7 Hz, 4H), 1.19 (dd, *J* = 8.3, 6.8 Hz, 24H). **^{13}C NMR** (101 MHz, $CDCl_3$) δ 145.31 (2 C), 141.35 (2 C), 127.21 (2 C), 126.73 (2 C), 125.25 (2 C), 119.71 (2 C), 66.99 (d, J=22.5 Hz, 1 C), 49.78 (2 C), 49.68 (2 C), 44.56 (d, J=12.4 Hz, 1 C), 24.59 (2 C), 24.51 (2 C), 23.95 (2 C), 23.90 (2 C). **^{31}P NMR** (122 MHz, CDCl3) δ 121.85.

((*i*Pr_2N)(FmO)P-Othio-DEACM) (9)

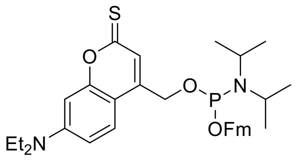

Compound 9

Compound 7 (1.02 g, 3.88 mmol, 1.0 eq.) and **Compound 8** (1.82 g, 4.27 mmol, 1.1 eq.) were separately co-evaporated with 2 x 1.5 ml dry CH_3CN. They were then dissolved in dry THF under Ar atmosphere and the mixture was cooled to 0 ºC. A dry solution of tetrazole (0.45 M in CH_3CN, 9.5 ml, 4.3 mmol, 1.1 eq.) was then added and it was stirred at 0 ºC for 2 h. A formed precipitate was quickly removed by filtration over neutral Al_2O_3 by using a glass filter funnel under reduced pressure. The filtrate was concentrated under reduced pressure and immediately purified by recrystallization (cyclohexane : EtOAc = 2:1, 15 ml). Compound **9** (1.63 g, 71 %) was obtained as yellow powder. **^{1}H NMR** (400 MHz, $CDCl_3$) δ 7.73 (dd, *J* = 7.6, 1.6 Hz, 2H), 7.65 (dd, *J* = 7.5, 1.0 Hz, 1H), 7.61 (dd, *J* = 7.5, 1.0 Hz, 1H), 7.40 – 7.33 (m, 3H), 7.29 (tt, *J* = 7.4, 1.5 Hz, 2H), 7.16 (d, *J* = 1.1 Hz, 1H), 6.67 (d, *J* = 2.5 Hz, 1H), 6.61 (dd, *J* = 9.1, 2.6 Hz, 1H), 4.71 – 4.55 (m, 2H), 4.19 (t, *J* = 6.6 Hz, 1H), 4.10 (dt, *J* = 9.8, 6.5 Hz, 1H), 3.88 (dt, *J* = 9.8, 6.9 Hz, 1H), 3.65 (dp, *J* = 10.2, 6.8 Hz, 2H), 3.42 (q, *J* = 7.1 Hz, 4H), 1.24 – 1.18 (m, 12H), 1.15 (d, *J* = 6.8 Hz, 6H). **^{13}C NMR** (101

MHz, $CDCl_3$) δ 197.59, 159.05, 150.80, 145.51, 145.44, 144.82, 144.47, 141.43 (d, J=5.5 Hz, 1 C), 127.53, 127.49, 127.01, 126.95, 125.42, 125.12, 124.92, 120.93, 119.90, 119.84, 110.03, 108.74, 97.44, 65.95 (d, J=17.4 Hz, 1 C), 61.25 (d, J=18.5 Hz, 1 C), 49.24, 49.16, 44.97, 43.38, 43.26, 24.85, 24.78, 24.69, 24.62, 12.52 (2 C). **^{31}P NMR** (162 MHz, $CDCl_3$) δ 148.59. **HRMS** (ESI): *m/z* for $C_{34}H_{41}N_2O_3PS$, $[M+H]^+$ calcd: 589.2654; found : 589.2643.

Stock solution of ADP&ATP&AP_4 • 2 TBA

ATP • 2 TBA solution: Adenosine 5'-triphosphate disodium salt (1.1 g, 2 mmol, 1 eq.) was dissolved in 50 ml Milli-Q water. This solution was passed through a column (9 × 2.5 cm) (30g) of Dowex® 50WX8 (H^+) 200-400 and the eluate was collected into a 250 ml flask. The elution of fully protonated adenosine 5'-triphosphate was easily monitored by the pH-value of the eluate. Subsequently, tetrabutylammonium (TBA) hydroxide 30-hydrate (3.52 g, 4.4 mmol, 2.2 eq.) was added to the eluate. Solid adenosine 5'-triphosphate TBA salt was obtained after lyophilization and the correct amount of TBA counter ion was determined by ^{1}H-NMR analysis, using PMe_4Br as internal standard. A stock solution of ATP • 2 TBA was prepared as a 0.2 **M** solution in dry DMF. Therefore, the obtained solid after lyophilization was co-evaporated 3 times with MeCN (5 ml), was then dissolved in dry DMF (10 ml) and stored over activated molecular sieve (3 Å) under an atmosphere of argon.

ADP • 2 TBA solution: procedure is same as ATP • 2 TBA solution.

AMP • 2 TBA solution: An aqueous solution of Adenosine 5′-monophosphate (694.44 mg, 2 mmol, 1 eq.) was mixed with tetrabutylammonium hydroxide 30-hydrate (3.52 g, 4.4 mmol, 2.2 eq.). After lyophilization, the correct amount of TBA counter ion was determined by ^{1}H-NMR analysis, using PMe_4Br as internal standard. A stock solution of AMP • 2 TBA was prepared as a 0.1 **M** solution in dry DMF. Therefore, the obtained solid after lyophilization was co-evaporated 3-times with MeCN (5 ml), dissolved in dry DMF (20 ml) and stored over activated molecular sieve (3 Å) under argon atmosphere.

Thio-DEACM-ATP

The synthesis of Thio-DEACM-ATP was adapted from a previously reported procedure by Alexandre Hofer [3]. With the developed method, ADP was coupled to thio-DEACM Fm phosphoramidite. Within 7 minutes, ADP was fully converted to a mixed P(III)-P(V) intermediate. Then mCPBA was used to oxidize P(III) to P(V), leading also to minor amounts of DEACM caged ATP, as mCPBA is able to convert the thio-carbonyl group into an oxo-carbonyl group.

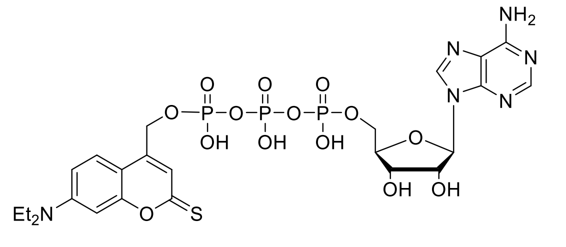

Thio-DEACM caged ATP

Compound 9 (648 mg, 1.1 mmol, 1.1 eq.) was added to a solution of ADP • 2 TBA (5 ml, 1 mmol, 1.0 eq.) in dry DMF. ETT (390 mg, 3 mmol, 3.0 eq.) was added to the reaction mixture, after it was co-evaporated with dry CH_3CN (3 x 1 ml). The mixture was stirred for 7 minutes at r.t. Afterwards, the solution was cooled to −10 °C, using a NaCl/ice mixture, and *m*CPBA (170 mg, 1 mmol, 1.0 eq.) was added. Afterwards, piperidine (500 µl, v/v=5%) was added and the mixture was stirred for 30 minutes. The product was then precipitated by adding the reaction mixture dropwise to a $NaClO_4$ (0.5 M) acetone solution (30 ml). The precipitate was isolated via centrifugation (7000 rpm, 5 min), the solid was washed with acetone (3 x 5 ml) and then dried under high vacuum. The crude product was purified by strong ion-exchange chromatography using an Äkta – system (NH_4HCO_3 – buffer, VIS detection at 700 nm). The product containing fractions, were combined and lyophilized. The obtained solid was further purified by RP-C18-chromatography using a MPLC system. It was eluted with increasing concentration of CH_3CN in H_2O with 10% TEAA buffer (0.1 M). Thio-DEACM caged ATP was obtained as: part 1: 55 mg, MW, 1083.69 (3.27 TEA^+), 0.051 mmol; part 2: 57.8 mg, MW, 922.22 (1.69 TEA^+), 0.063 mmol; part 3: 48 mg, MW, 804 (3 NH_4^+), 0.060 mmol. In total: thio-DEACM caged ATP (0.174 mmol, 18%), as orange solid. **^{1}H NMR** (400 MHz, D_2O) δ 8.33 (s, 1H), 8.12 (s, 1H), 7.14 (d, J = 9.1 Hz, 1H), 6.80 (s, 1H), 6.48 (dd, J = 9.2, 2.4 Hz, 1H), 6.32 (d, J = 2.4 Hz, 1H), 5.84 (d, J = 4.6 Hz, 1H), 5.13 – 4.92 (m, 2H), 4.43 (dt, J = 14.3, 4.9 Hz, 2H), 4.36 – 4.28 (m, 2H), 4.22 (ddd, J = 11.0, 5.8, 2.5 Hz, 1H), 3.28 (q, J = 7.1 Hz, 4H), 1.07 (t, J = 7.1 Hz, 6H). **^{13}C NMR** (101 MHz, D_2O) δ 196.41, 158.34, 151.67, 151.28, 148.08, 147.58, 147.47 (d, *J* = 9.7 Hz, 1C), 140.43, 124.80, 117.87, 117.22, 111.51, 107.54, 95.74, 87.73, 83.53 (d, *J* = 9.4 Hz, 1C), 75.24, 69.77, 65.09 (d, *J* = 5.2 Hz, 1C), 62.99 (d, *J* = 4.3 Hz, 1C), 44.75 (2C), 11.74 (2C). **^{31}P NMR** (162 MHz, D_2O) δ -11.26 (d, J = 17.0 Hz, 1P), -11.69 (d, J = 17.6 Hz, 1P), -22.65 (t, J = 16.8 Hz, 1P). **HRMS** (ESI): *m/z* for $C_{24}H_{31}N_6O_{14}P_3S$, $[M-H]^-$ calcd: 751.0759; found : 751.0764. $[M+H]^+$ calcd: 753.0910; found : 753.0915.

DEACM-ATP

As side product, DEACM caged ATP (15.5 mg, MW, 931.71 (1.94 TEA^+), 17 µmol, 2%) was also obtained.

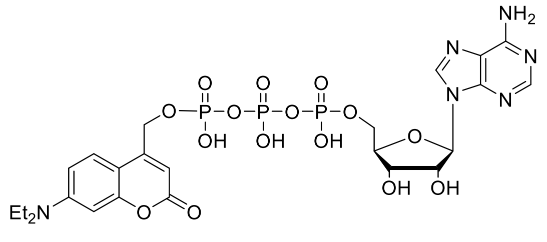

DEACM caged ATP

^{1}H NMR (400 MHz, D_2O) δ 8.20 (s, 1H), 7.98 (s, 1H), 7.05 (d, J = 9.1 Hz, 1H), 6.39 (dd, J = 9.1, 2.6 Hz, 1H), 6.20 (d, J = 2.5 Hz, 1H), 6.05 (s, 1H), 5.74 (d, J = 5.0 Hz, 1H), 4.98 (qdd, J = 16.0, 6.6, 1.3 Hz, 2H), 4.40 – 4.29 (m, 2H), 4.29 – 4.23 (m, 2H), 4.19 (ddd, J = 11.4, 5.8, 3.0 Hz, 1H), 3.25 (q, J = 7.3 Hz, 4H), 1.05 (t, J = 7.1 Hz, 5H). **^{13}C NMR** (101 MHz, D_2O) δ 165.41, 154.78, 154.18, 154.02 (d, *J* = 9.0 Hz, 1C), 151.21, 150.60, 147.89, 139.24, 124.35, 117.99, 109.47, 104.96, 103.01, 96.26, 87.16, 83.39(d, *J* = 9.4 Hz, 1C),

75.03, 69.90, 65.21(d, J = 5.5 Hz, 1C), 63.30(d, J = 4.6 Hz, 1C), 44.42 (2C), 11.75 (2C). **^{31}P NMR** (162 MHz, D_2O) δ -11.47 (d, J = 19.0 Hz, 1P), -11.72 (d, J = 19.9 Hz, 1P), -23.20 (t, J = 19.4 Hz, 1P). **HRMS** (ESI): *m/z* for $C_{24}H_{31}N_6O_{15}P_3$, $[M-H]^-$ calcd: 735.0987; found : 735.0992. $[M+H]^+$ calcd: 737.1138; found : 737.1143.

Thio-DEACM-ADP

The procedure is identical to the synthesis of thio-DEACM-ATP.

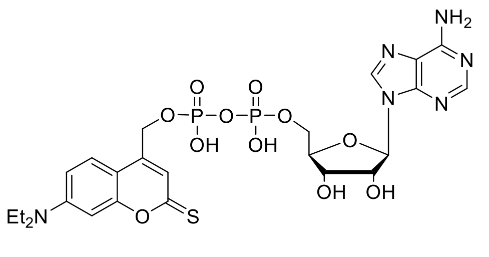

Thio-DEACM caged ADP

Start from: AMP • 2 TBA (2 ml, 0.2 mmol, 1.0 eq.), thio-DEACM caged ADP was obtained as: (27 mg, MW, 706.61 (2 NH_4^+), 38 µmol, 19%). **^{1}H NMR** (400 MHz, D_2O) δ 8.18 (s, 1H), 7.95 (s, 1H), 6.97 (d, J = 9.2 Hz, 1H), 6.89 (s, 1H), 6.43 (d, J = 9.2 Hz, 1H), 6.13 (d, J = 2.1 Hz, 1H), 5.84 (d, J = 4.2 Hz, 1H), 5.03 – 4.91 (m, 2H), 4.42 (dd, J = 11.6, 4.8 Hz, 3H), 4.47 – 4.33 (m, 4H), 4.24 (d, J = 12.5 Hz, 1H), 3.30 (q, J = 7.1 Hz, 4H), 1.14 (t, J = 7.1 Hz, 6H). **^{13}C NMR** (101 MHz, D_2O) δ 198.68, 160.47, 156.83, 154.18, 153.81, 150.19, 149.62 (d, J = 9.0 Hz, 1C), 141.27, 126.57, 120.42, 119.36, 113.69, 109.35, 97.68, 89.88, 85.34 (d, J = 8.8 Hz, 1C), 77.41, 71.77, 67.08 (d, J = 4.5 Hz, 1C), 65.22(d, J = 3.4 Hz, 1C), 47.06 (2C), 14.40 (2C). **^{31}P NMR** (122 MHz, D_2O) δ -11.65 (s, 2P). **HRMS** (ESI): *m/z* for $C_{24}H_{30}N_6O_{11}P_2S$, $[M-H]^-$ calcd: 671.1096; found : 671.1097.

DEACM-ADP

As side product, DEACM caged ADP (6 mg, MW, 865.00 (2.06 TEA^+), 0.007 mmol, 3%) was also obtained.

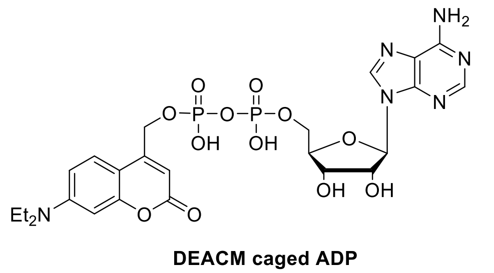

DEACM caged ADP

^{1}H NMR (300 MHz, D_2O) δ 8.05 (s, 1H), 7.81 (s, 1H), 6.78 (d, J = 9.1 Hz, 1H), 6.21 (dd, J = 9.1, 2.5 Hz, 1H), 5.94 (d, J = 2.5 Hz, 2H), 5.69 (d, J = 4.2 Hz, 1H), 4.83 (s, 2H), 4.31 – 4.24 (m, 4H), 4.13 – 4.06 (m, 1H), 3.15 (q, J = 7.3 Hz, 4H), 0.99 (t, J = 7.1 Hz, 6H). **^{13}C NMR** (101 MHz, D_2O) δ 165.43, 154.86, 154.62, 153.72 (dd, J=1.9 Hz, 3.5Hz, 1C), 152.10, 150.65, 147.85, 138.67, 123.87, 117.98, 109.21, 104.50, 102.66, 96.01, 87.07, 82.96 (dd, J=1.7 Hz, 4.1Hz, 1C), 74.81, 69.45, 64.66 (br s, 1C), 63.08 (br s, 1C), 44.28 (2C), 11.77

(2C). **^{31}P NMR** (122 MHz, D_2O) δ -11.70 (s, 2P). **HRMS** (ESI): *m/z* for $C_{24}H_{30}N_6O_{12}P_2$, $[M-H]^-$ calcd: 655.1324; found : 655.1327. $[M+H]^+$ calcd: 657.1475; found : 657.1469.

Thio-DEACM-AP$_4$

The procedure is identical to the synthesis of thio-DEACM-ATP.

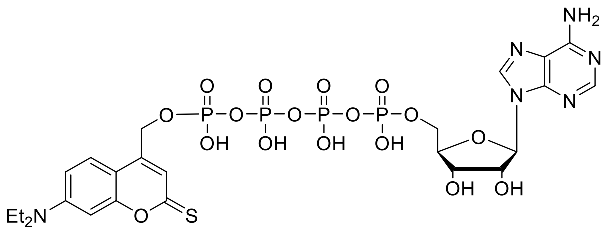

Thio-DEACM caged AP$_4$

Start from: ATP • 2 TBA (2 ml, 0.4 mmol, 1.0 eq.), thio-DEACM caged AP$_4$ was obtained as: (90 mg, MW, 1170.72 (3.36 TEA^+), 77 µmol, 20%). **^{1}H NMR** (300 MHz, D_2O) δ 8.30 (s, 1H), 8.00 (s, 1H), 7.24 (d, *J* = 9.2 Hz, 1H), 6.83 (s, 1H), 6.48 (dd, *J* = 9.3, 2.5 Hz, 1H), 6.24 (d, *J* = 2.5 Hz, 1H), 5.83 (d, *J* = 5.5 Hz, 1H), 5.07 (d, *J* = 6.9 Hz, 2H), 4.55 (t, *J* = 5.3 Hz, 1H), 4.45 – 4.39 (m, 1H), 4.30 – 4.20 (m, 3H), 3.22 (q, *J* = 6.9 Hz, 4H), 1.02 (t, *J* = 7.1 Hz, 6H). **^{13}C NMR** (101 MHz, D_2O) δ 196.26, 158.46, 153.74, 151.42, 150.90, 148.05, 147.98, 139.64, 125.12, 117.90, 117.21, 111.63, 107.54, 95.45, 86.88, 83.64 (d, *J* = 9.2 Hz), 74.74, 70.25, 65.12 (d, *J* = 5.5 Hz), 63.23 (d, *J* = 4.7 Hz), 44.60 (2C), 11.80 (2C). **^{31}P NMR** (122 MHz, D_2O) δ -11.13 – -11.52 (m, 1P), -11.53 – -11.94 (m, 1P), -22.98 – -23.56 (m, 2P). **HRMS** (ESI): *m/z* for $C_{24}H_{32}N_6O_{17}P_4S$, $[M-H]^-$ calcd: 831.0422; found : 831.0427. $[M+H]^+$ calcd: 833.0573; found : 833.0561.

DEACM-AP$_4$

As side product, DEACM caged AP$_4$ (20 mg, MW, 1172.39 (3.5 TEA^+), 0.017 mmol, 4%) was also obtained.

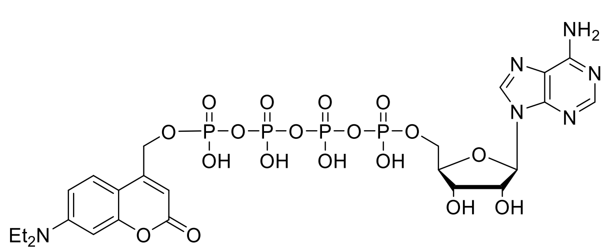

DEACM caged AP$_4$

^{1}H NMR (300 MHz, D_2O) δ 8.21 (s, 1H), 7.87 (s, 1H), 7.11 (d, J = 9.1 Hz, 1H), 6.37 (dd, J = 9.2, 2.5 Hz, 1H), 6.10 (d, J = 2.4 Hz, 1H), 6.03 (s, 1H), 5.73 (d, J = 5.8 Hz, 1H), 4.99 (d, J = 7.2 Hz, 2H), 4.49 (t, J = 5.5 Hz, 1H), 4.39 – 4.34 (m, 1H), 4.21 – 4.11 (m, 3H), 3.16 (q, J = 7.0 Hz, 4H), 0.97 (t, J = 7.0 Hz, 6H). **^{13}C NMR** (101 MHz, D_2O) δ 165.25, 154.95, 154.47 (d, *J* = 8.6 Hz, 1C), 153.47, 150.68, 150.37, 147.97, 139.85,

124.82, 117.85, 109.60, 105.15, 102.81, 96.31, 86.91, 83.77 (d, *J* = 9.3 Hz, 1C), 74.90, 70.38, 65.15 (d, *J* = 5.4 Hz, 1C), 63.59 (d, *J* = 4.8 Hz, 1C)., 44.39 (2C), 11.77 (2C). **^{31}P NMR** (122 MHz, D_2O) δ -11.31 – -11.69 (m, 1P), -11.69 – -12.14 (m, 1P), -23.07 – -23.75 (m, 2P). **HRMS** (ESI): *m/z* for $C_{24}H_{32}N_6O_{18}P_4$, $[M-H]^-$ calcd: 815.0651; found : 815.0657. $[M+H]^+$ calcd: 817.0802; found : 817.0787.

4. Spectroscopic Characterization

4.1 Absorption and Fluorescence Spectra

Absorption and fluorescence spectra of compounds were measured by Spark® multimode microplate reader. Thermo Scientific™ 96-Well plates were used for measurements. UV-absorption: transparent plates, 50 µM. Fluorescence: black plates, concentration depends on fluorescence quantum yield for different samples.

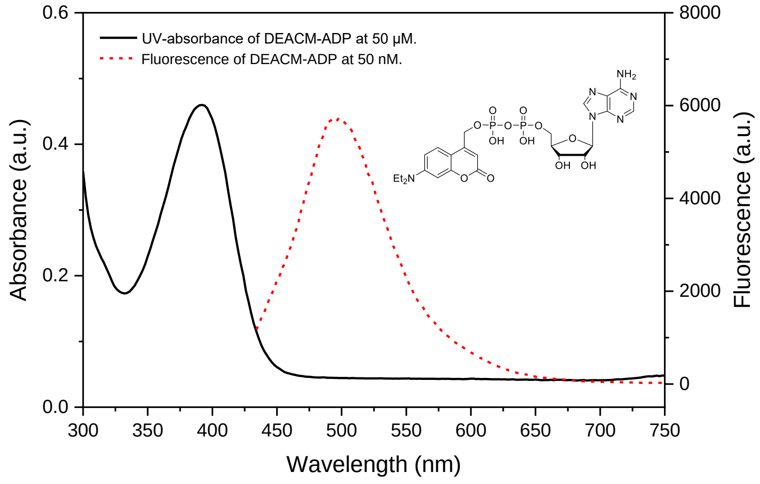

Figure S1. UV-absorbance and fluorescence spectra of DEACM caged ADP in H_2O.

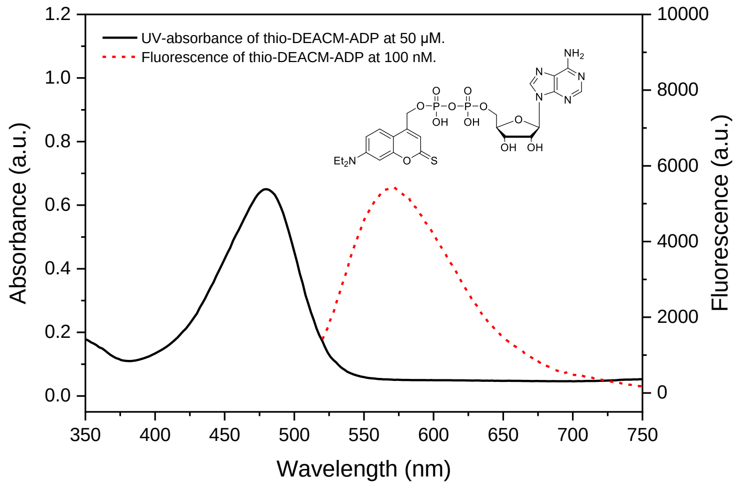

Figure S2. UV-absorbance and fluorescence spectra of thio-DEACM caged ADP in H_2O.

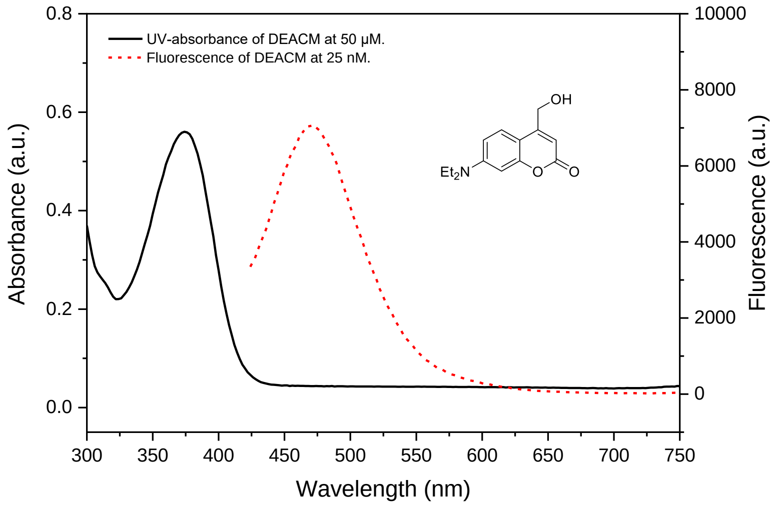

Figure S3. UV-absorbance and fluorescence spectra of DEACM in methanol.

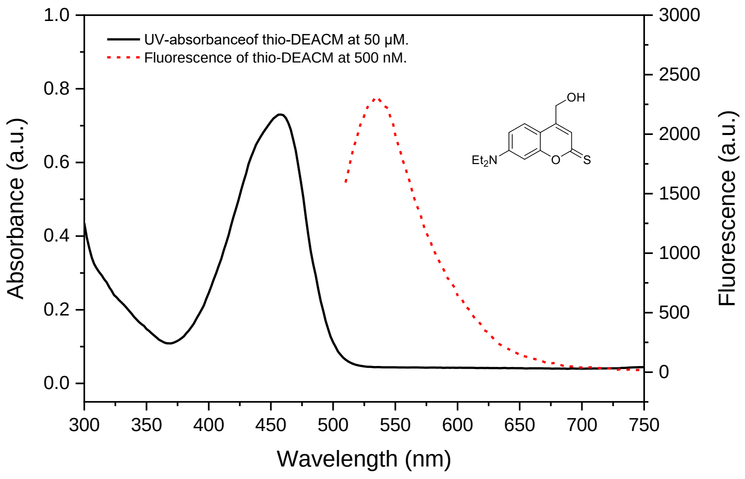

Figure S4. UV-absorbance and fluorescence spectra of thio-DEACM in methanol.

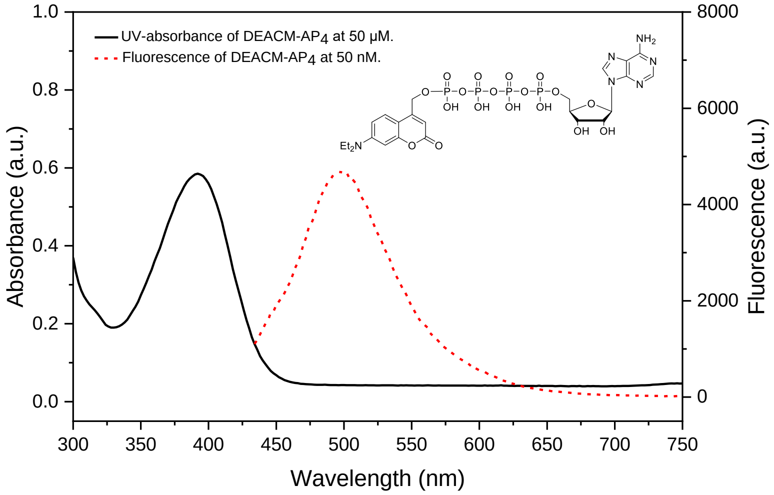

Figure S5. UV-absorbance and fluorescence spectra of DEACM caged AP_4 in H_2O.

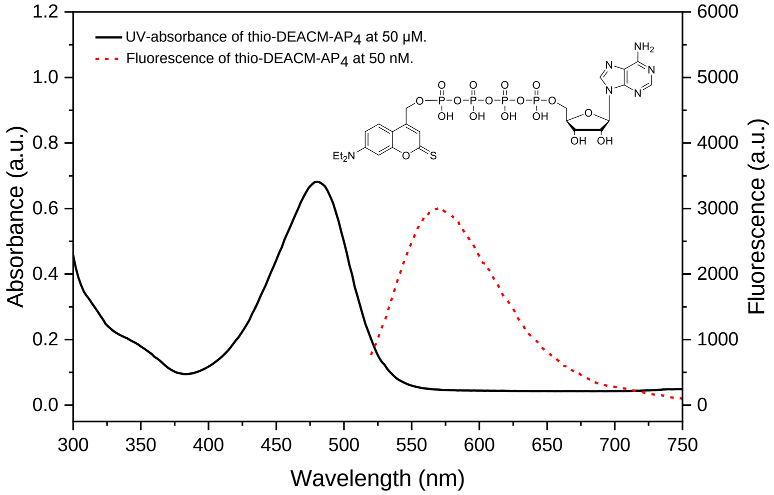

Figure S6. UV-absorbance and fluorescence spectra of thio-DEACM caged AP_4 in H_2O.

4.2 Molar absorption coefficient

The absorption was measured on Thermo Scientific™ Genesys 10s UV-VIS Spectrophotometer. And then molar absorption coefficient was calculated according to Lambert–Beer law: $\varepsilon = \frac{A}{c*l}$.

4.3 Fluorescence quantum yield

Fluorescence Quantum Yield was measured with the JASCO Spectrofluorometer FP-8300 with liquid sample cell (1 mm thickness, 1 x 10 x 25 mm for ILF-533/IL) for samples. In this program, the quantum yield is determined using the excitation light spectrum (incident light spectrum) and the sample emission spectrum measured under identical conditions. (From [Quantum Yield Calculation] Program Software Manual.)

$$\text{Internal quantum efficiency} = \frac{\text{The number of photons in emission}}{\text{The number of photons in of the excitation light absorbed by the sample}}$$

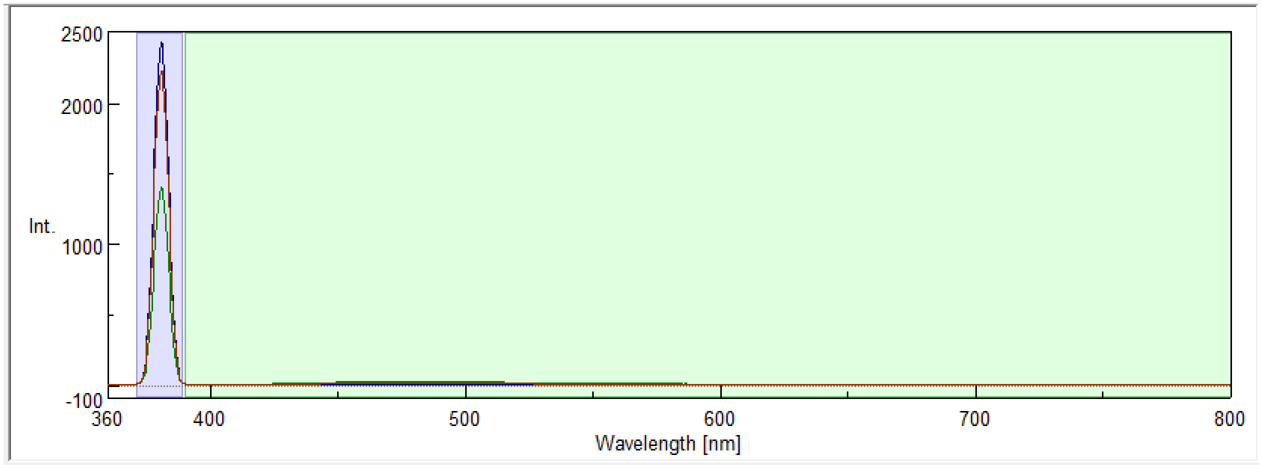

	File Name	Absorptance (%)	Ext. Quantum Efficiency (%)	Int. Quantum Efficiency (%)
1	DEACM.qydt	37.380	9.555	25.563
2	Thio-DEACM.qydt	44.093	0.237	0.538
3	DEACM-ADP.qydt	29.938	5.303	17.714
4	Thio-DEACM-ADP.qydt	30.507	0.831	2.724
5	DEACM-ATP.qydt	40.192	4.968	12.361
6	Thio-DEACM-ATP.qydt	30.970	0.686	2.216
7	DEACM-AP4.qydt	31.628	4.206	13.300
8	Thio-DEACM-AP4.qydt	29.246	0.640	2.190

Figure S7. Fluorescence quantum yield of photocages and photocaged compounds.

4.4 DFT Calculations

DFT calculations were performed with the Gaussian 16 program package [9]. The geometries of all molecules were optimized without symmetry restrictions with the Becke–3–parameter–Lee–Yang–Parr (B3LYP [4,5]) functional and the def2–TZVPP [6,7] basis set. Stationary points were confirmed by vibrational frequency analysis. The polarizable continuum model [8] (PCM) for water as implemented in Gaussian 16 was used in all calculations. Time–dependent DFT calculations were performed to calculate the first 15 singlet excitations of each molecule. The spectra were plotted using SpecDis Version 1.70 [10] applying a gaussian bandshape.

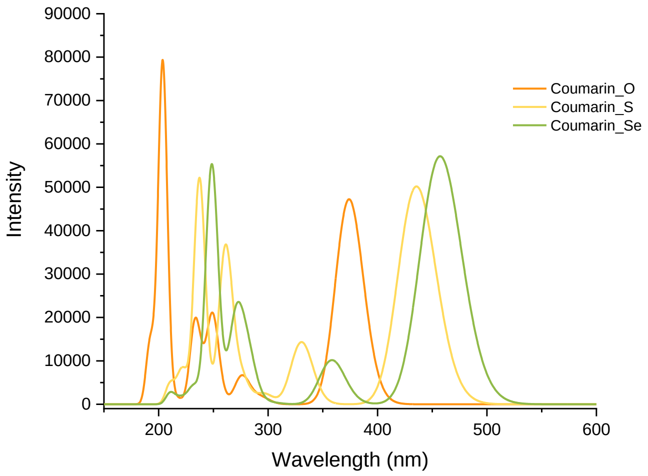

Figure S8. Plotted spectra.

4.5 Calculated Structures

(Electronic Energy = -824.496341662 E_h, ZPVE = 0.289585 E_h)			
**C**	0.72002	0.75467	-0.13148
**C**	-0.64599	0.95314	-0.0895
**H**	-1.00455	1.96932	-0.10193
**C**	-1.52516	-0.14756	-0.01397
**N**	-2.87831	0.01998	0.025
**C**	-0.93504	-1.44439	0.01605
**H**	-1.55802	-2.32296	0.05552
**C**	0.42671	-1.61189	-0.02283
**H**	0.82776	-2.61443	0.00994
**C**	1.31221	-0.51946	-0.09814
**C**	2.74123	-0.60481	-0.15646
**C**	3.47782	0.53642	-0.23891
**C**	3.4428	-1.94312	-0.12573
**H**	4.50829	-1.79717	-0.31397
**H**	3.05094	-2.58899	-0.9116
**O**	3.23563	-2.65043	1.09718
**C**	2.87189	1.84017	-0.26654
**O**	1.49267	1.88322	-0.20654
**O**	3.45057	2.90734	-0.33483
**H**	4.55661	0.51196	-0.28661
**H**	3.63309	-2.14454	1.81341
**C**	-3.49765	1.33796	-0.10681
**C**	-3.79802	-1.10343	0.20521
**H**	-3.3525	-1.83375	0.87902
**C**	-4.22357	-1.76915	-1.10306
**H**	-4.67719	-0.71813	0.72198
**H**	-2.92565	1.93741	-0.81415
**C**	-3.65808	2.08053	1.21973
**H**	-4.47643	1.18968	-0.5631
**H**	-3.3683	-2.19022	-1.63155
**H**	-4.71149	-1.05237	-1.7646
**H**	-4.92948	-2.57572	-0.89967
**H**	-2.69357	2.2642	1.69289
**H**	-4.27304	1.50761	1.91474
**H**	-4.14435	3.04272	1.05278

Table S1. Calculated Cartesian Coordinates for Coumarin_O on the B3LYP/def2-TZVPP level of theory.

(Electronic Energy = -1147.44280786 E_h, ZPVE = 0.287565 E_h)			
**C**	0.56987	0.46886	-0.0933
**C**	-0.75276	0.85886	-0.05831
**H**	-0.9652	1.91512	-0.05215
**C**	-1.7762	-0.11125	-0.01326
**N**	-3.09071	0.23817	0.019
**C**	-1.37093	-1.48075	-0.00703
**H**	-2.11198	-2.26302	0.00849
**C**	-0.04884	-1.83859	-0.03839
**H**	0.20877	-2.88733	-0.02404
**C**	0.982	-0.87655	-0.0829
**C**	2.37918	-1.14903	-0.1315
**C**	3.26276	-0.10254	-0.1826
**C**	2.90168	-2.56674	-0.12725
**H**	3.9816	-2.55521	-0.28642
**H**	2.45102	-3.13172	-0.94353
**O**	2.57079	-3.27668	1.06591
**C**	2.83409	1.24742	-0.18607
**O**	1.49467	1.4785	-0.1378
**S**	3.83299	2.58269	-0.2413
**H**	4.32664	-0.27892	-0.22135
**H**	3.0168	-2.85412	1.80723
**C**	-3.5225	1.63256	-0.08445
**C**	-4.16034	-0.75115	0.16448
**H**	-3.82833	-1.55207	0.82289
**C**	-4.65946	-1.31692	-1.16418
**H**	-4.98158	-0.25826	0.68463
**H**	-2.86659	2.16376	-0.77291
**C**	-3.59226	2.35612	1.25995
**H**	-4.50765	1.62952	-0.55062
**H**	-3.86614	-1.84155	-1.69658
**H**	-5.03376	-0.52231	-1.81056
**H**	-5.47425	-2.02028	-0.98715
**H**	-2.61596	2.39639	1.74266
**H**	-4.28495	1.85455	1.93662
**H**	-3.94275	3.37877	1.11454

Table S2. Calculated Cartesian Coordinates for Coumarin_S on the B3LYP/def2-TZVPP level of theory.

(Electronic Energy = -3150.80749241 E_h, ZPVE = 0.286869 E_h)			
**C**	-0.13844	-0.12549	-0.06089
**C**	1.08557	-0.75853	-0.02955
**C**	2.2739	0.0022	-0.01117
**N**	3.49809	-0.58694	0.01498
**C**	2.134	1.42487	-0.02619
**C**	0.9047	2.02617	-0.05342
**C**	-0.29086	1.27489	-0.07175
**C**	-1.60873	1.80413	-0.11241
**C**	-2.67656	0.93778	-0.13736
**C**	-1.85737	3.29459	-0.12661
**O**	-1.38131	3.94722	1.0497
**C**	-2.50213	-0.45864	-0.12027
**O**	-1.23981	-0.9408	-0.07958
**Se**	-3.85781	-1.69333	-0.14471
**C**	3.65786	-2.04005	-0.06339
**C**	4.73759	0.18565	0.1278
**C**	5.30979	0.62819	-1.21785
**C**	3.60333	-2.73772	1.29522
**H**	1.09505	-1.83553	-0.00577
**H**	3.00998	2.05244	-0.03028
**H**	0.84973	3.10464	-0.05615
**H**	-3.68801	1.31171	-0.16926
**H**	-2.92272	3.48268	-0.27244
**H**	-1.32171	3.7527	-0.95834
**H**	-1.89296	3.63392	1.8028
**H**	4.62112	-2.23109	-0.53565
**H**	2.90652	-2.44998	-0.73702
**H**	5.45999	-0.44638	0.64375
**H**	4.57371	1.04361	0.77743
**H**	5.51593	-0.23171	-1.85601
**H**	6.2454	1.16767	-1.06485
**H**	4.62005	1.28554	-1.7471
**H**	4.38492	-2.36315	1.95704
**H**	3.7533	-3.81059	1.16823
**H**	2.64185	-2.58407	1.78481

Table S3. Calculated Cartesian Coordinates for Coumarin_Se on the B3LYP/def2-TZVPP level of theory.

5. Photolysis

5.1 Aqueous stability of thio-DEACM caged compounds

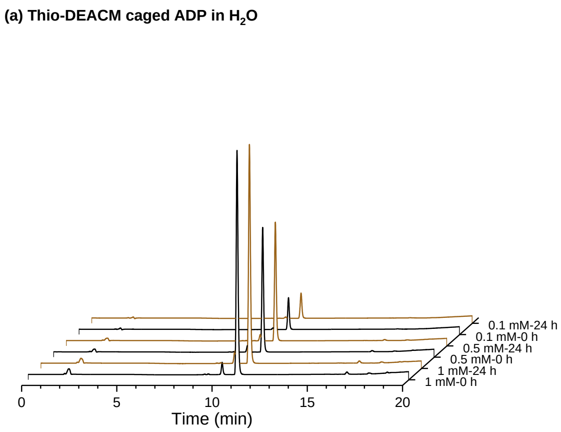

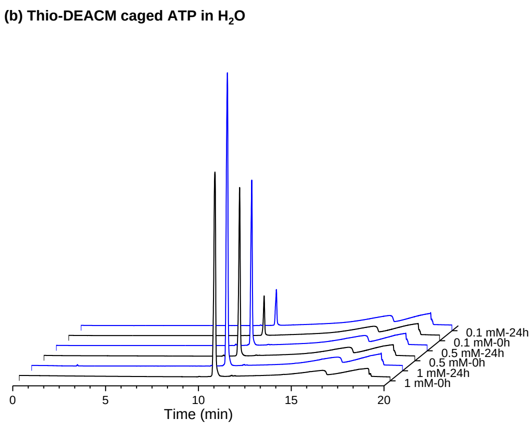

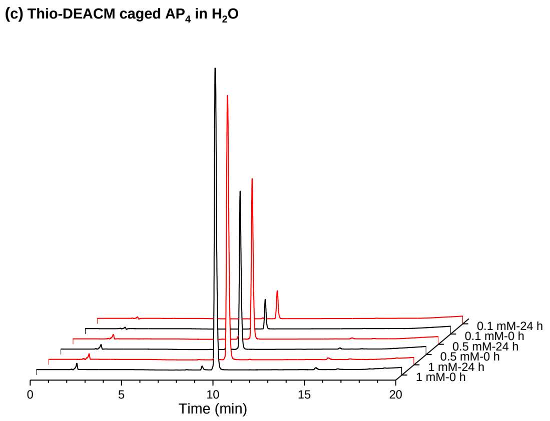

Figure S9. Stability of thio-DEACM caged nucleotides in the dark within 24 hours.

5.2 Experimental details

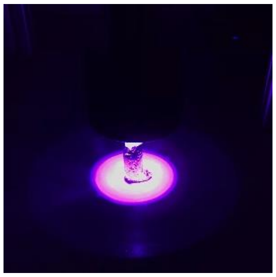

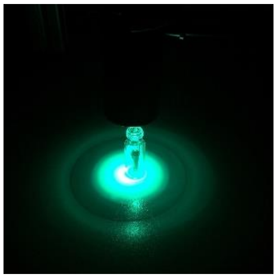

Figure S10. LED irradiation for uncaging experiments, 400 nm and 490 nm.

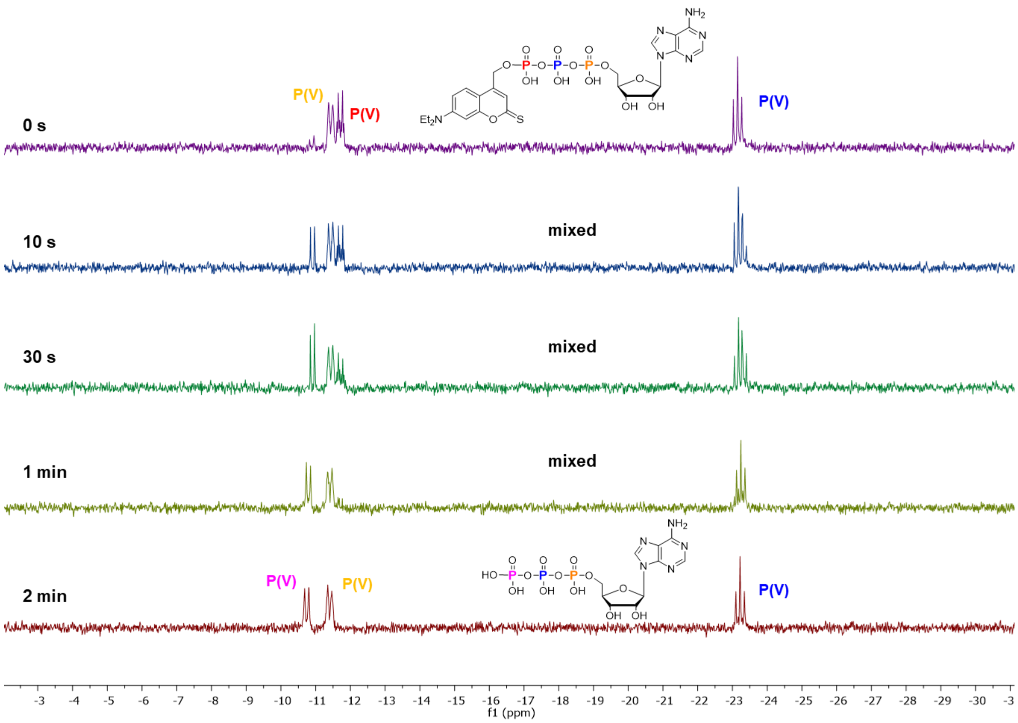

Figure S11. Uncaging process of thio-DEACM caged ATP monitored by ^{31}P proton coupled NMR. (2 mg/ml, in D_2O, 490 nm, 50% light intensity.)

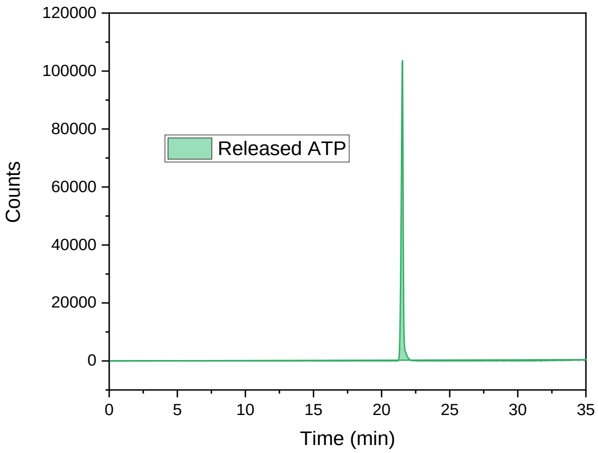

Figure S12. CE-MS identification of uncaged ATP.

5.3 Uncaging of thio-DEACM caged ATP at 490 nm

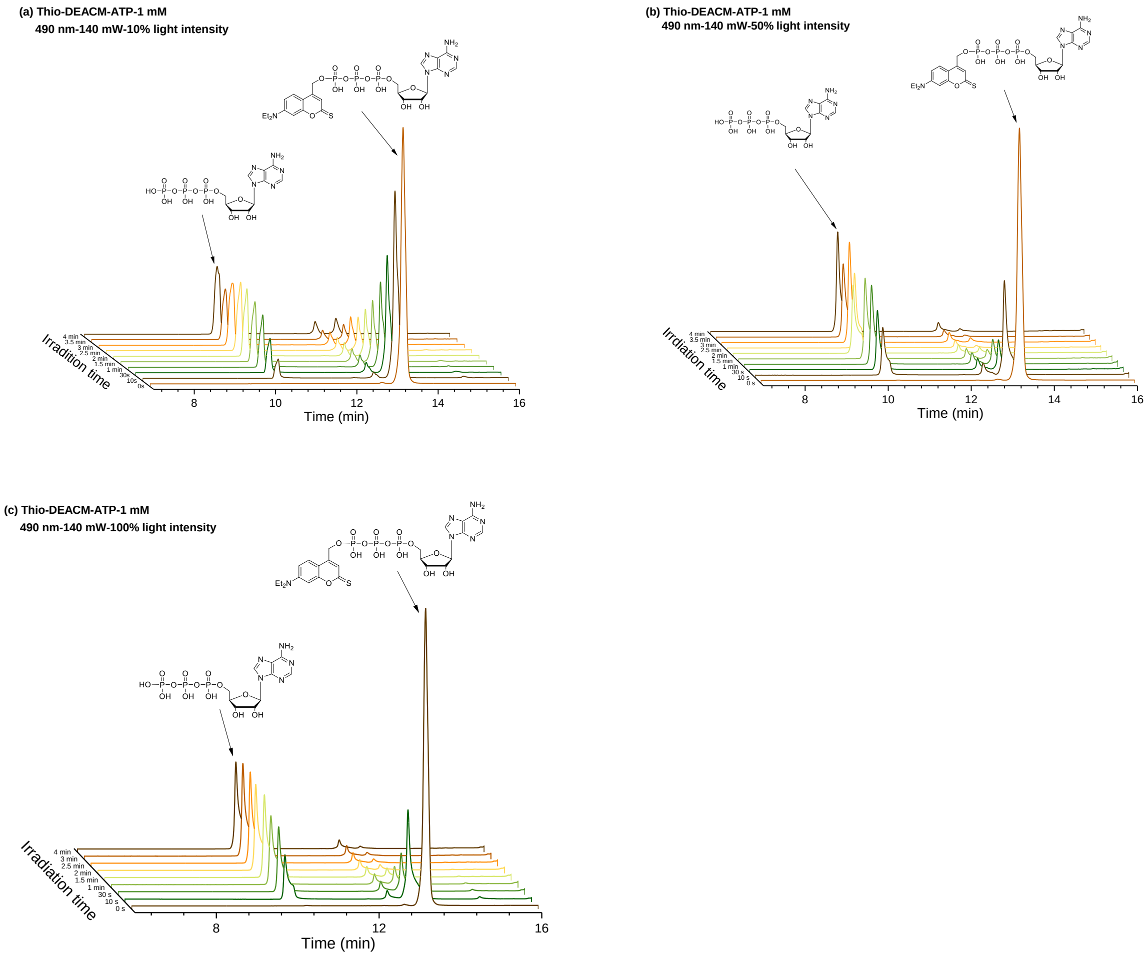

Figure S13. HPLC analysis of the uncaging of thio-DEACM caged ATP at 490 nm.

Thio-DEACM-ATP	Time (x 10^2 S)	Peak Area (mAU)	Percentage (left) (%)	Uncaging ratio (%)
	0	*255.335*	*100*	*0*
	0.1	181.900	71.24	28.76
	0.6	74.977	29.36	70.64
	0.9	51.193	20.05	79.95
	1.2	40.219	15.75	84.25
**10% light intensity**	1.5	29.289	11.47	88.53
	1.8	23.793	9.32	90.68
	2.1	12.227	4.79	95.21
	2.4	12.774	5.00	95.00
	0	*255.335*	*100*	*0*
	0.1	99.304	38.89	61.11
	0.6	24.406	9.56	90.44
	0.9	9.232	3.62	96.38
**50% light intensity**	1.2	4.41	1.73	98.27
	1.5	1.834	0.72	99.28
	1.8	4.064	1.59	98.41
	2.1	1.559	0.61	99.39
	2.4	2.157	0.84	99.16
	0	*255.335*	*100*	*0*
	0.1	83.739	32.80	67.20
	0.6	14.364	5.63	94.37
	0.9	6.573	2.57	97.43
**100% light intensity**	1.2	3.765	1.47	98.53
	1.5	2.746	1.08	98.92
	1.8	2.021	0.79	99.21
	2.1	1.603	0.63	99.37
	2.4	1.373	0.54	99.46

Table S4. Data extracted from HPLC analysis of the uncaging of thio-DEACM caged ATP at 490 nm.

5.4 Uncaging of thio-DEACM caged ADP at 490 nm

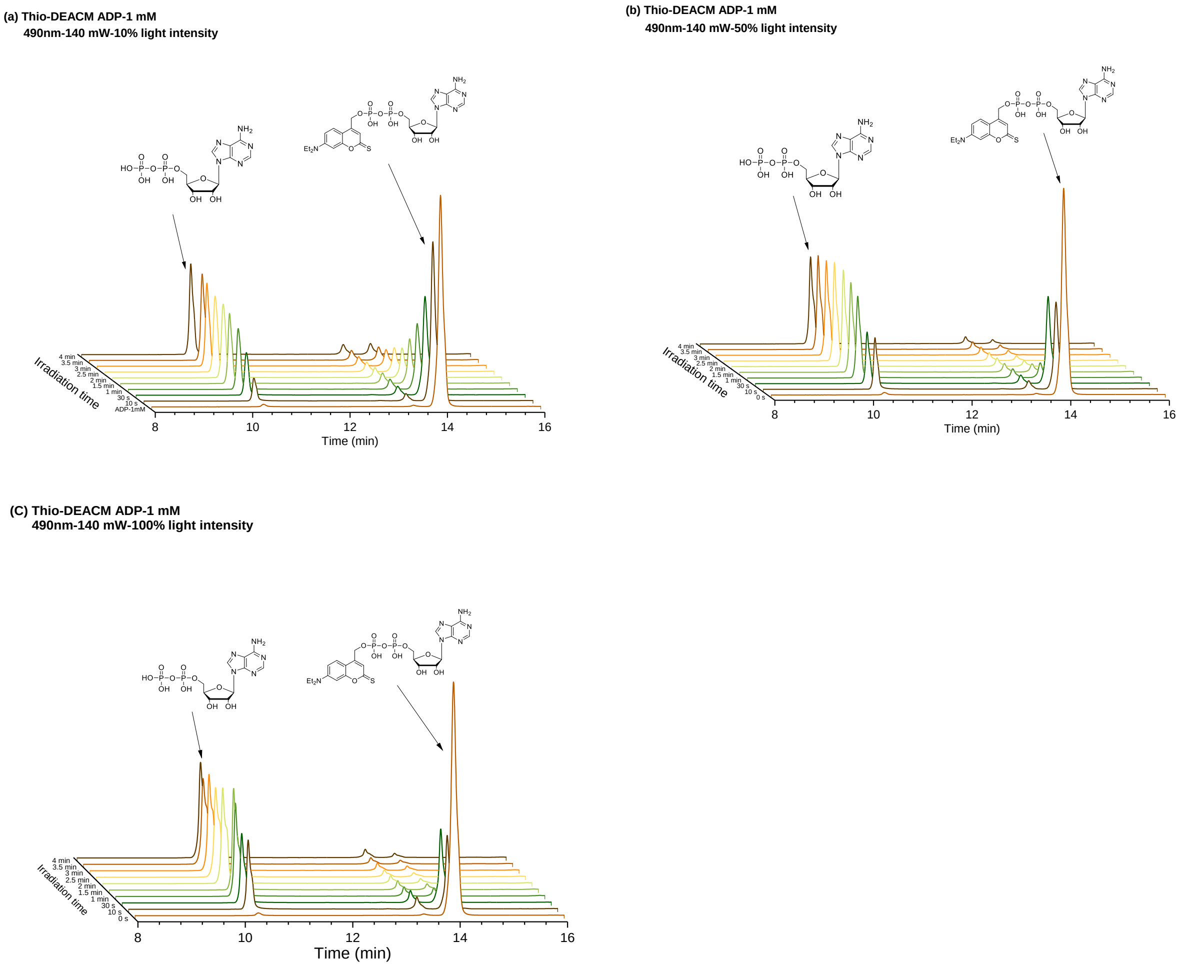

Figure S14. HPLC analysis of the uncaging of thio-DEACM caged ADP at 490 nm.

Thio-DEACM-ADP	Time (x 10^2 S)	Peak Area (mAU)	Percentage (left) (%)	Uncaging ratio (%)
	0	*208.419*	*100*	*0*
	0.1	154.835	74.29	25.71
	0.3	101.773	48.83	51.17
	0.6	66.373	31.85	68.15
	0.9	42.414	20.35	79.65
**10% light intensity**	1.2	29.192	14.01	85.99
	1.5	24.386	11.70	88.3
	1.8	15.595	7.48	92.52
	2.1	11.857	5.69	94.31
	2.4	9.754	4.68	95.32
	0	*208.419*	*100*	*0*
	0.1	86.339	41.43	58.57
	0.3	35.709	17.13	82.87
	0.6	14.283	6.85	93.15
**50% light intensity**	0.9	7.35	3.53	96.47
	1.2	4.965	2.38	97.62
	1.5	3.99	1.91	98.09
	1.8	3.306	1.59	98.41
	2.1	3.015	1.45	98.55
	2.4	2.765	1.33	98.67
	0	*208.419*	*100*	*0*
	0.1	59.492	28.54	71.46
	0.3	18.216	8.74	91.26
	0.6	6.511	3.12	96.88
**100% light intensity**	0.9	4.354	2.09	97.91
	1.2	3.587	1.72	98.28
	1.5	3.389	1.63	98.37
	1.8	3.194	1.53	98.47
	2.1	2.834	1.36	98.64
	2.4	2.653	1.27	98.73

Table S5. Data extracted from HPLC analysis of the uncaging of thio-DEACM caged ADP at 490 nm.

5.5 Uncaging of thio-DEACM AP_4 at 490 nm

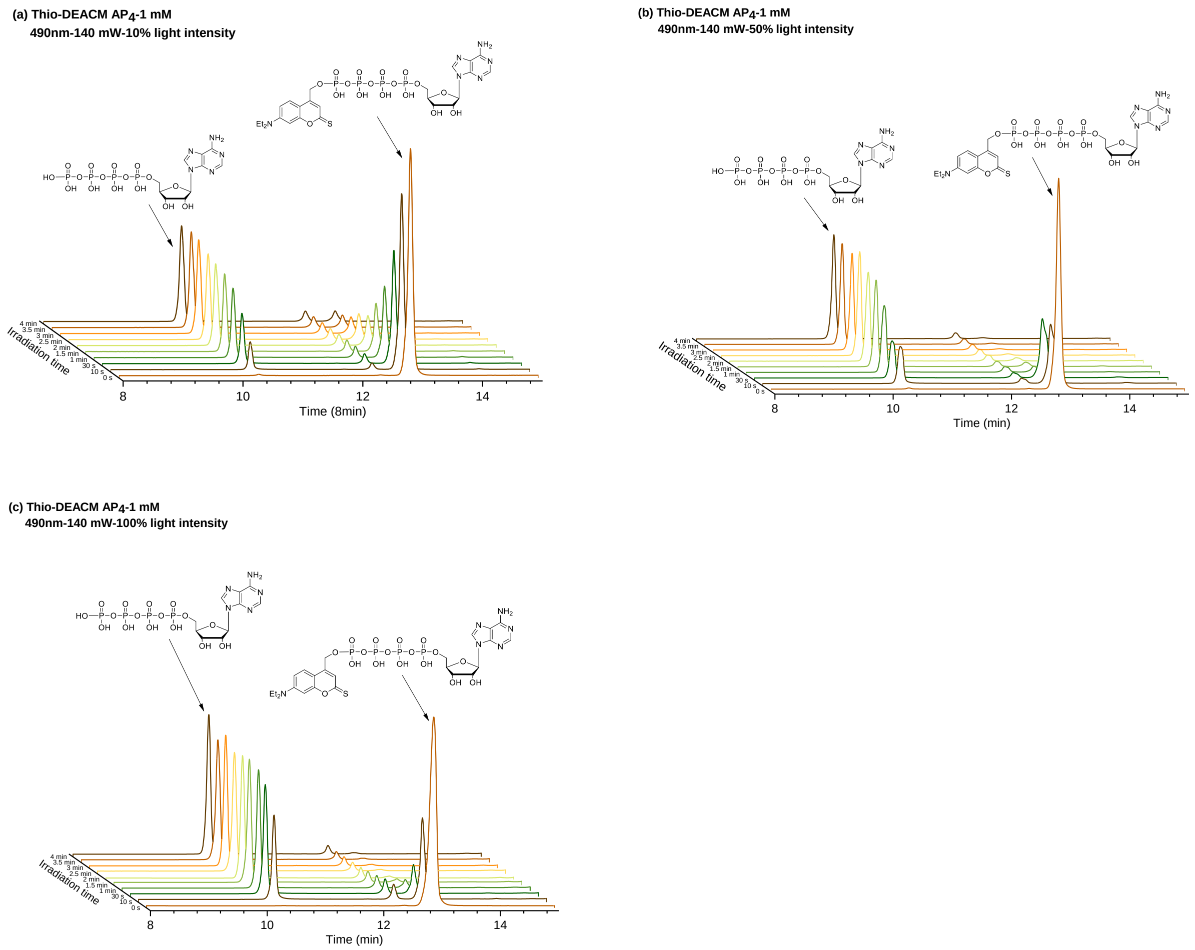

Figure S15. HPLC analysis of the uncaging of thio-DEACM caged AP_4 at 490 nm.

Thio-DEACM-AP_4	Time (x 10^2 S)	Peak Area (mAU)	Percentage (left) (%)	Uncaging ratio (%)
	0	*196.137*	*100*	*0*
	0.1	142.676	72.74	27.26
**10% light intensity**	0.3	90.458	46.12	53.88
	0.6	57.268	29.20	70.8
	0.9	40.169	20.48	79.52
	1.2	28.201	14.38	85.62
	1.5	23.254	11.86	88.14
	1.8	14.411	7.35	92.65
	2.1	10.886	5.55	94.45
	2.4	9.419	4.80	95.2
	0	*192.444*	*100*	*0*
**50% light intensity**	0.1	77.47	39.50	60.5
	0.3	42.683	21.76	78.24
	0.6	7.687	3.92	96.08
	0.9	4.851	2.47	97.53
	1.2	3.785	1.93	98.07
	1.5	1.336	0.68	99.32
	1.8	0.842	0.43	99.57
	2.1	0.821	0.42	99.58
	2.4	0	0	100
	0	*192.444*	*100*	*0*
**100% light intensity**	0.1	58.708	30.51	69.49
	0.3	20.191	10.49	89.51
	0.6	5.959	3.10	96.9
	0.9	2.219	1.15	98.85
	1.2	1.389	0.72	99.28
	1.5	0.96	0.50	99.50
	1.8	0.745	0.39	99.61
	2.1	0.595	0.31	99.69
	2.4	0	0	100

Table S6. Data extracted from HPLC analysis of the uncaging of thio-DEACM caged AP_4 at 490 nm.

5.6 Uncaging of DEACM caged ADP, ATP, AP_4 at 490 nm

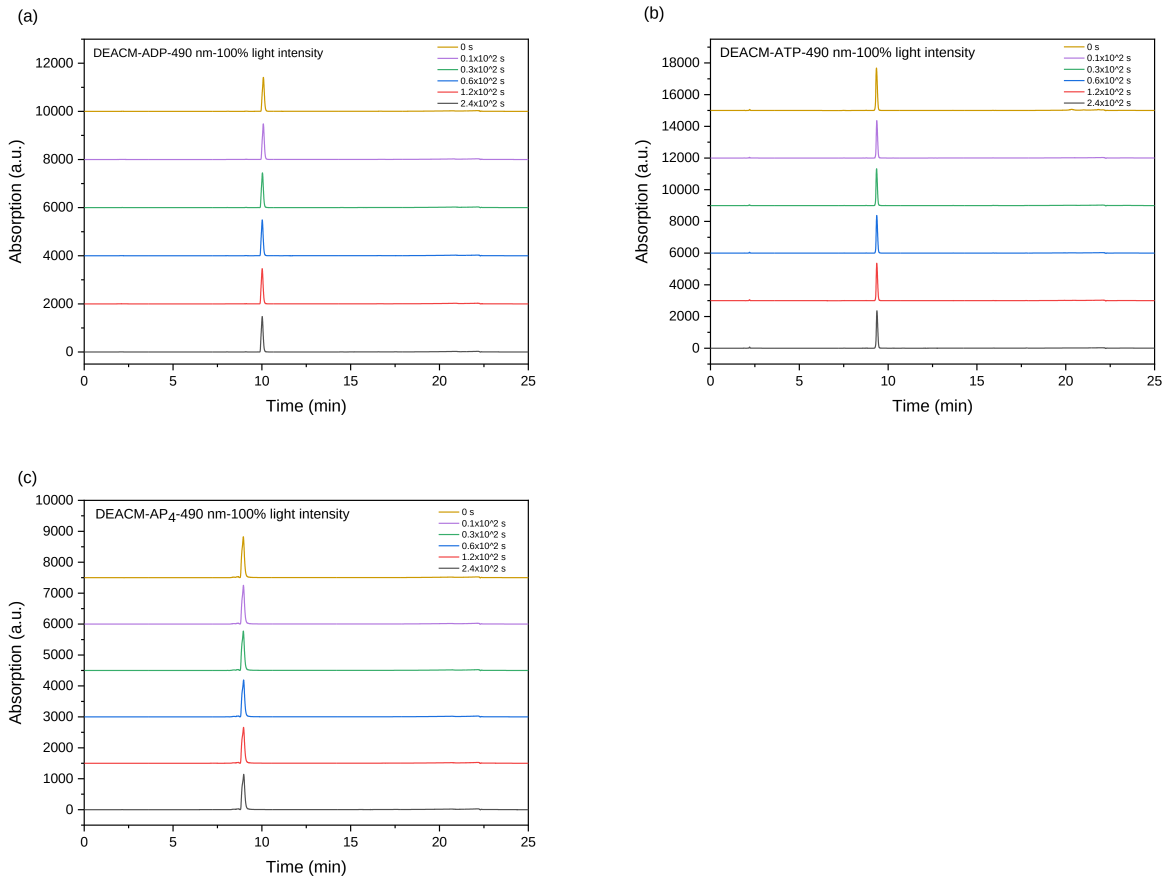

Figure S16. DEACM caged nucleotides photolysis at 490 nm within 4 minutes.

5.7 Uncaging of DEACM caged ATP and thio-DEACM caged ATP at 400 nm

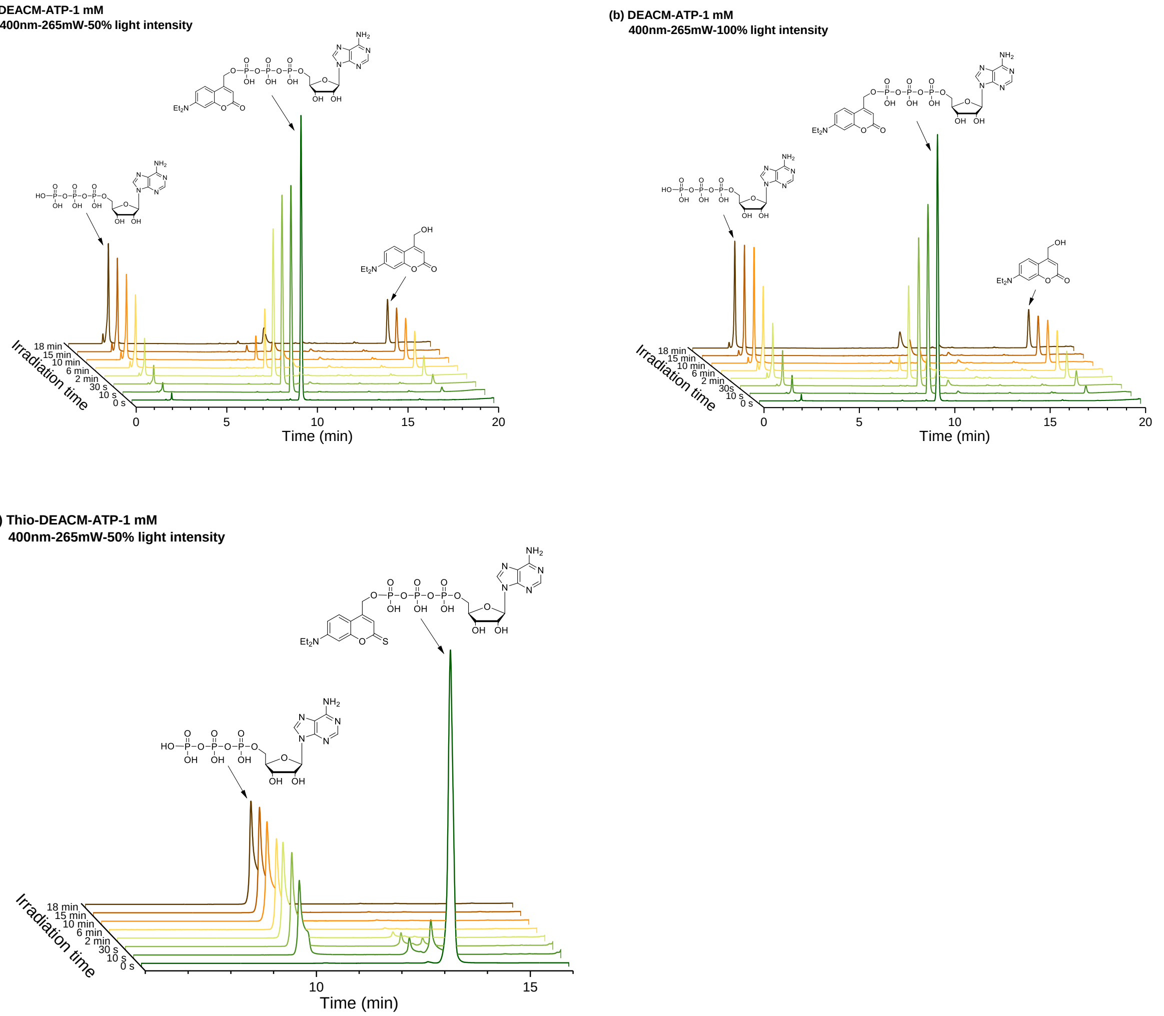

Figure S17. HPLC analysis of the uncaging of DEACM and thio-DEACM caged ATP at 400nm.

DEACM-ATP	Time (x10^2 S)	Peak Area (mAU)	Percentage (left) (%)	Uncaging ratio (%)
	0	*239.244*	*100*	*0*
**50% light intensity**	0.1	212.222	88.71	11.29
	0.3	188.912	78.96	21.04
	1.2	138.001	57.68	42.32
	3.6	49.578	20.72	79.28
	6	19.248	8.05	91.95
	9	4.744	1.98	98.02
	10.8	1.856	0.78	99.22
	0	*239.244*	*100*	*0*
**100% light intensity**	0.1	214.583	89.69	10.31
	0.3	155.501	65.00	35
	1.2	87.121	36.42	63.58
	3.6	11.567	4.83	95.17
	6	1.649	0.69	99.31
	9	0	0	100
	10.8	0	0	100
	0	*255.335*	*100*	*0*
**Thio-DEACM-ATP**	0.1	25.769	10.09	89.91
	0.3	4.853	1.90	98.1
	1.2	0	0	100
**50% light intensity**	3.6	0	0	100
	6	0	0	100
	9	0	0	100
	10.8	0	0	100

Table S7. Data extracted from HPLC analysis of the uncaging of DEACM and thio-DEACM caged ATP at 400nm.

5.8 Explanation of absence of thio-DEACM in HPLC analysis

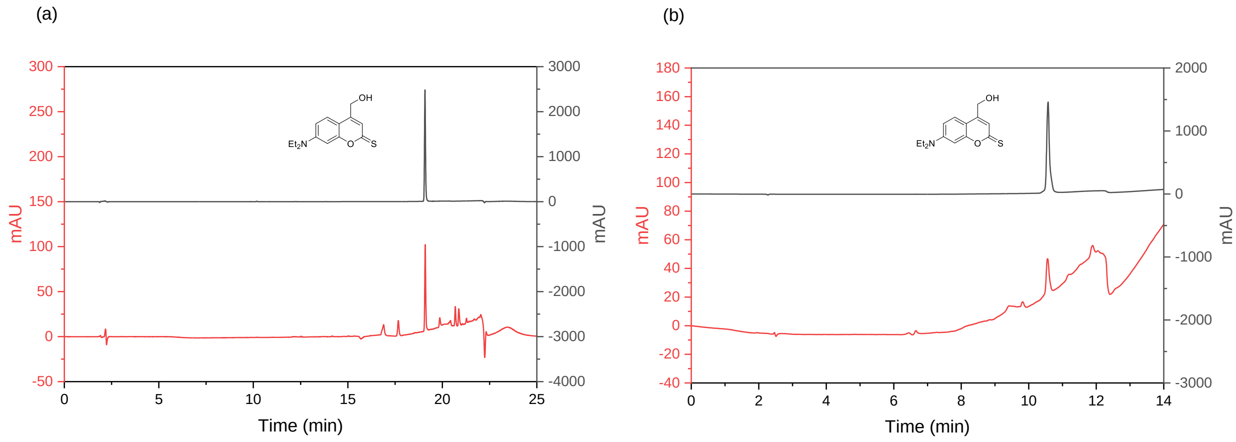

Figure S18. HPLC analysis of uncaged product. Black: thio-DEACM, Red: uncaged product from photolysis, extracted with DCM.

.

6. NMR spectra of the compounds

((*E*)-7-(Diethylamino)-4-[2-(dimethylamino)vinyl]-2*H*-chromen-2-one) (2)

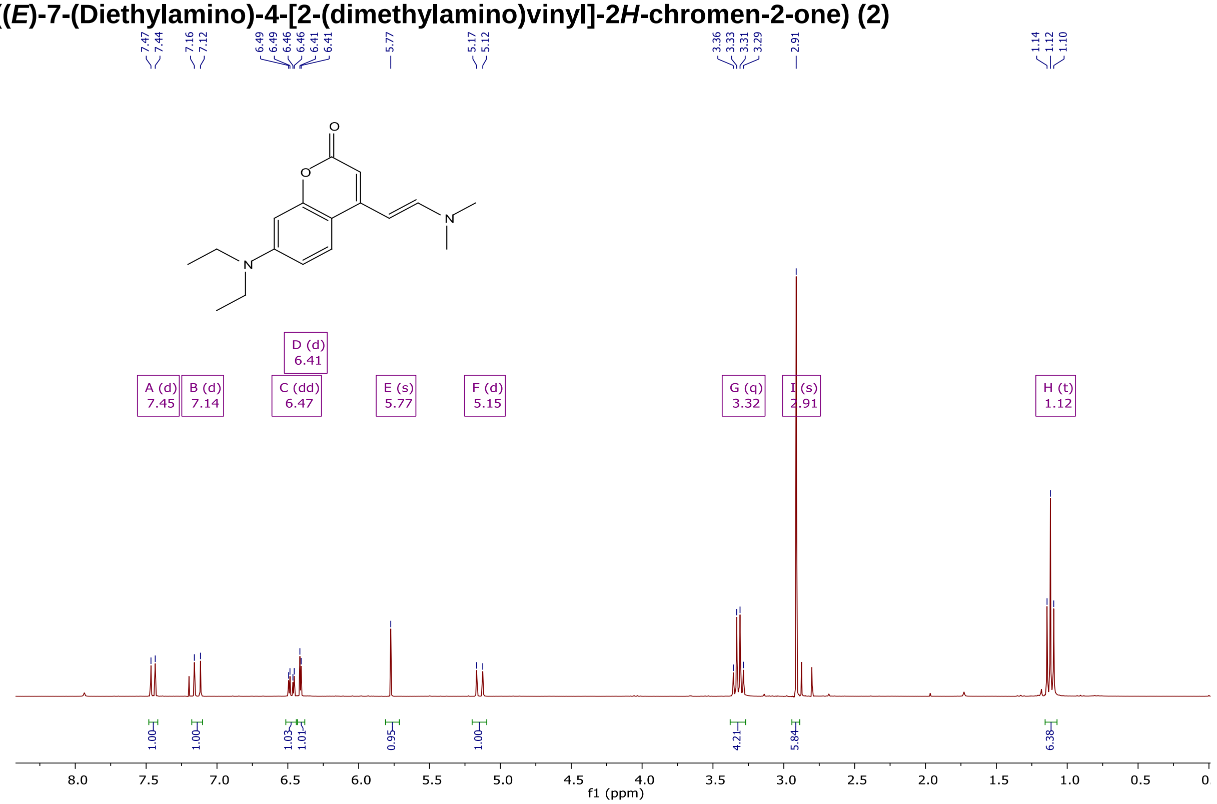

^{1}H NMR spectra of **compound 2**

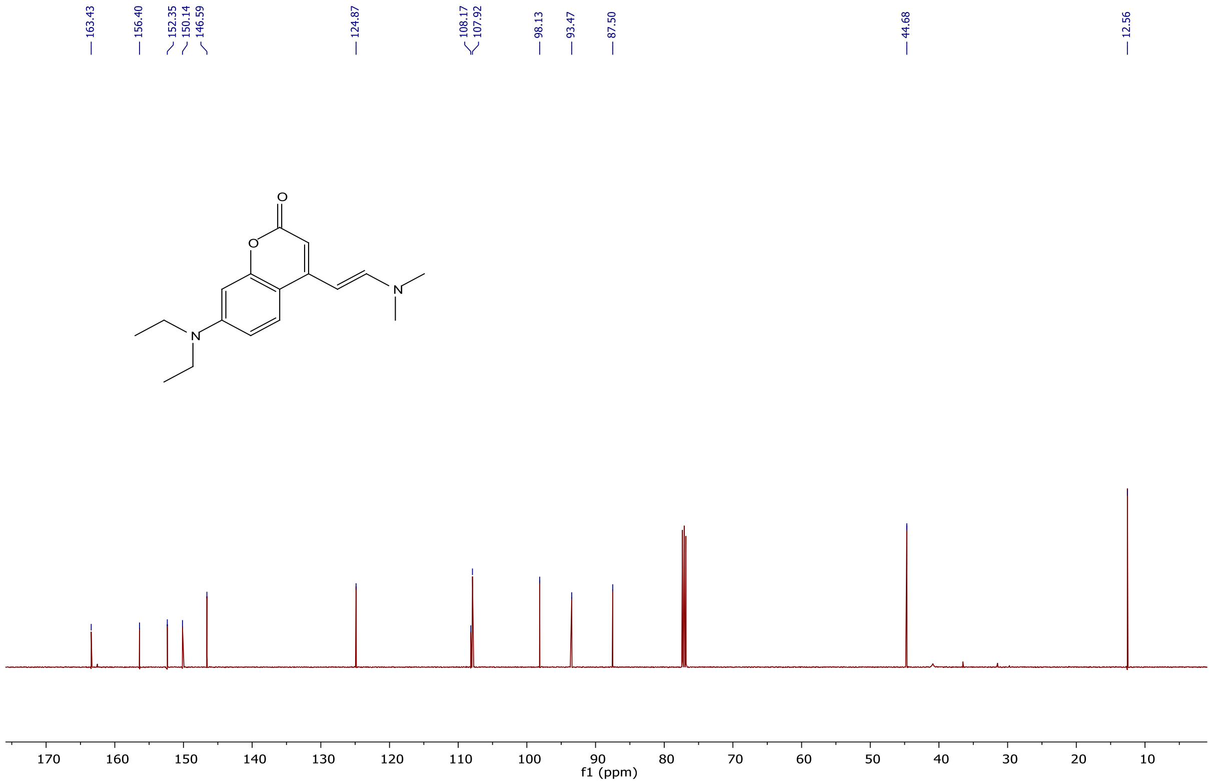

^{13}C NMR spectra of **compound 2**

(7-(Diethylamino)-2-oxo-2*H*-chromene-4-carbaldehyde) (3)

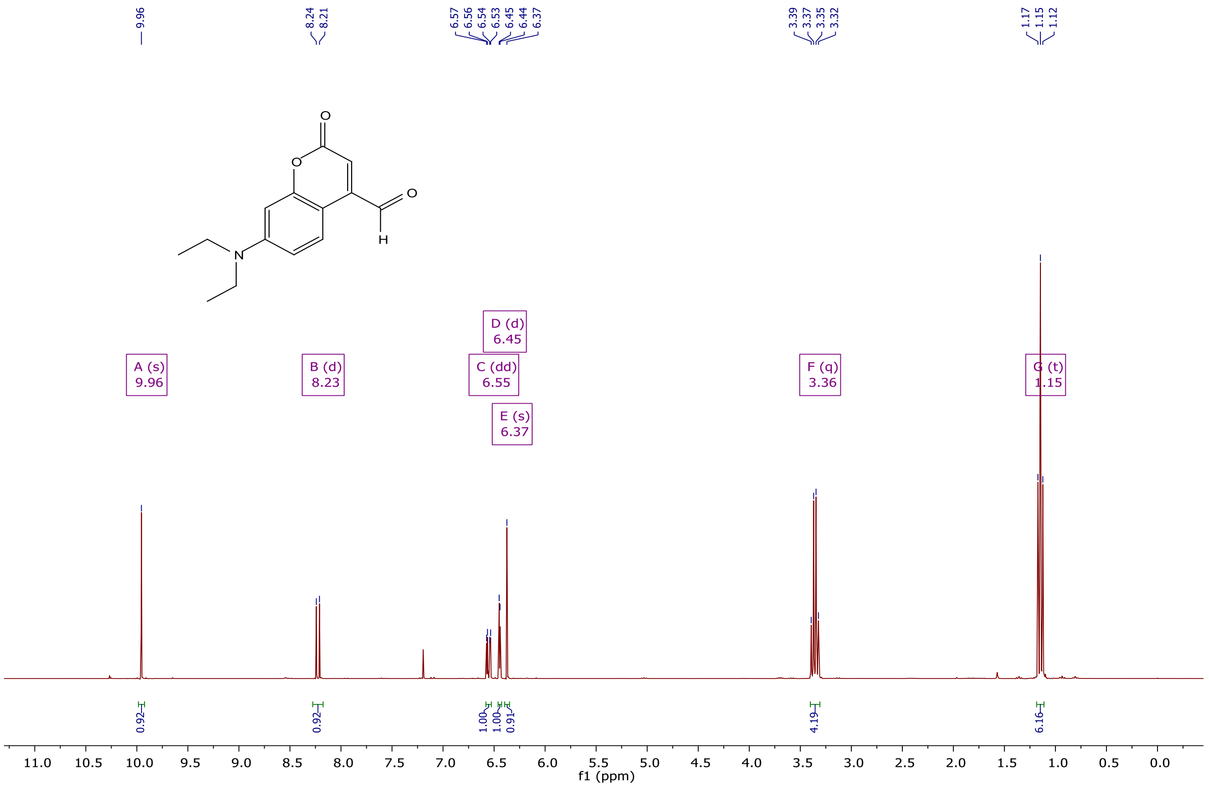

^{1}H NMR spectra of **compound 3**

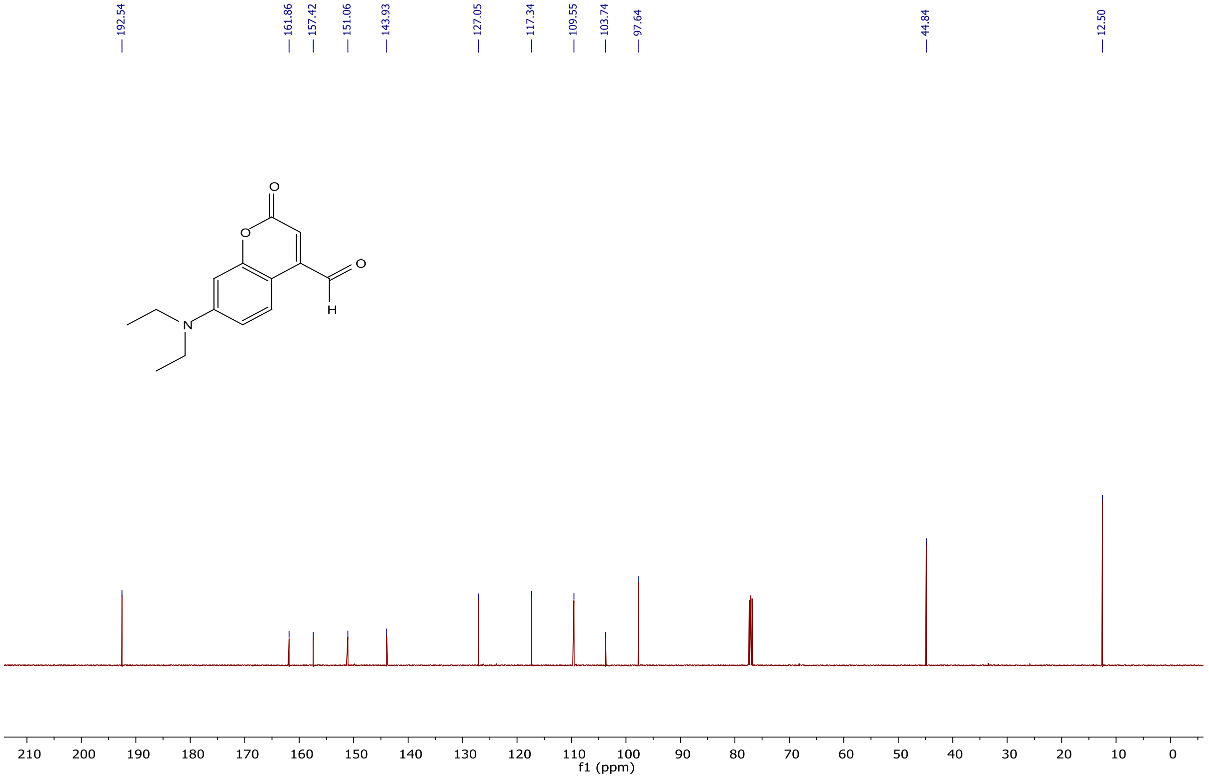

^{13}C NMR spectra of **compound 3**

(7-(Diethylamino)-4-(hydroxymethyl)-2*H*-chromen-2-one) (4)

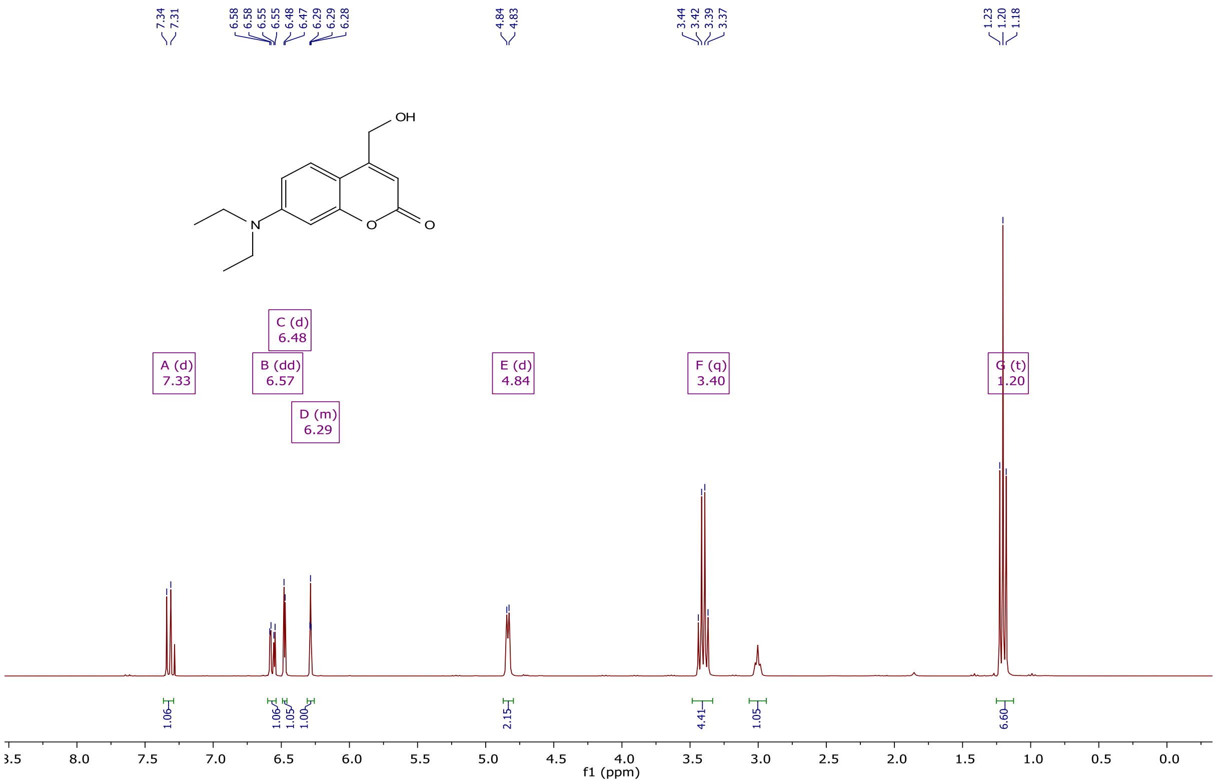

^{1}**H** NMR spectra of **compound 4**

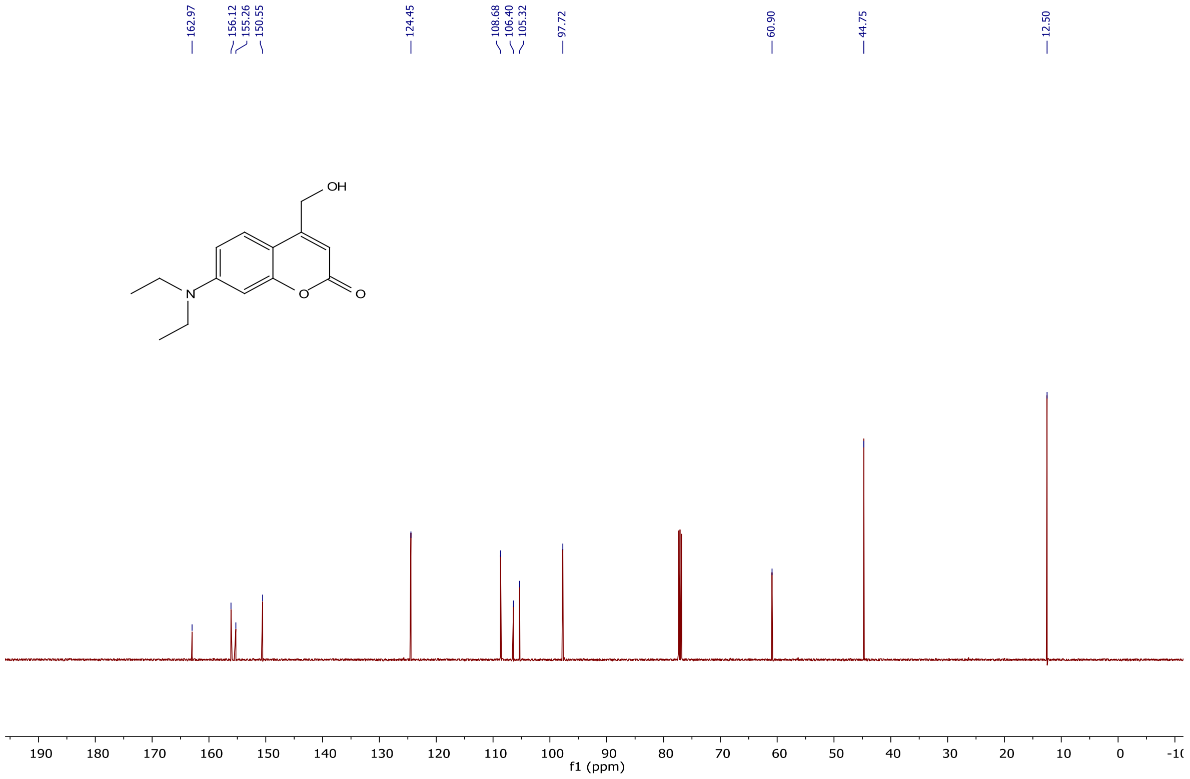

^{13}**C** NMR spectra of **compound 4**

(7-Diethylamino-4-methylacetoate-coumarin) (5)

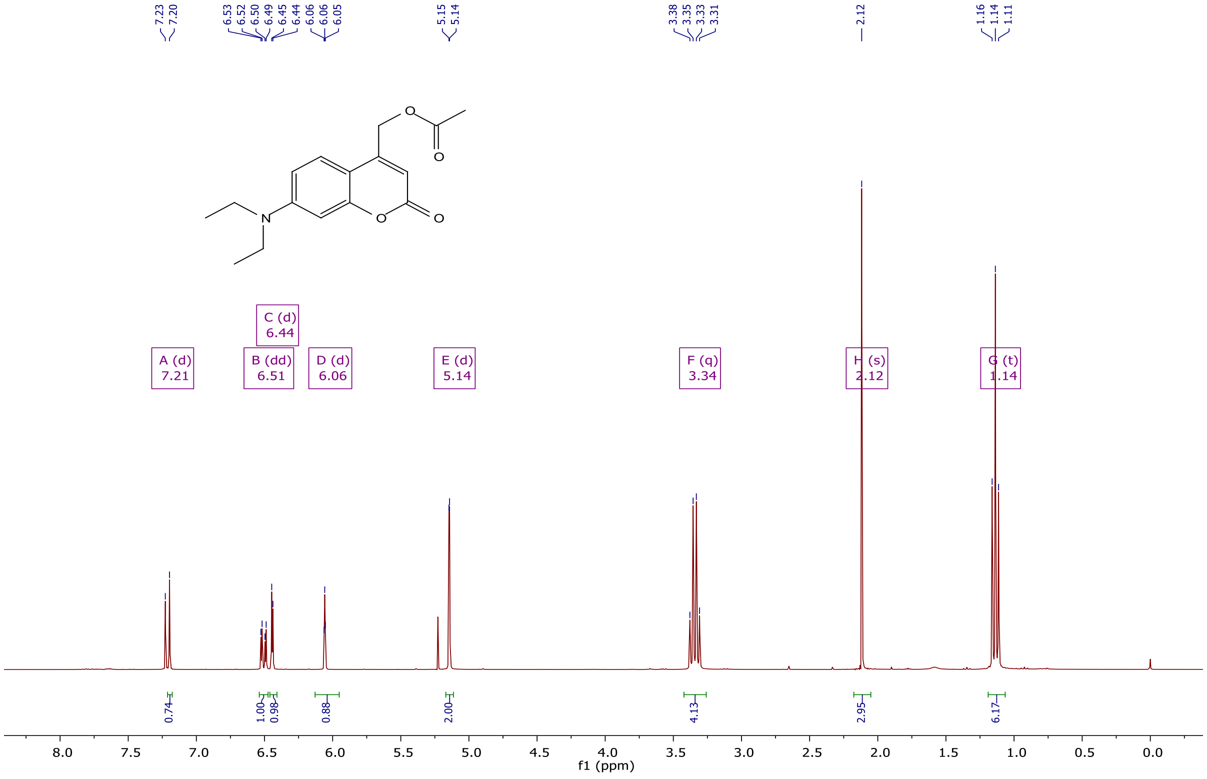

^{1}H NMR spectra of **compound 5**

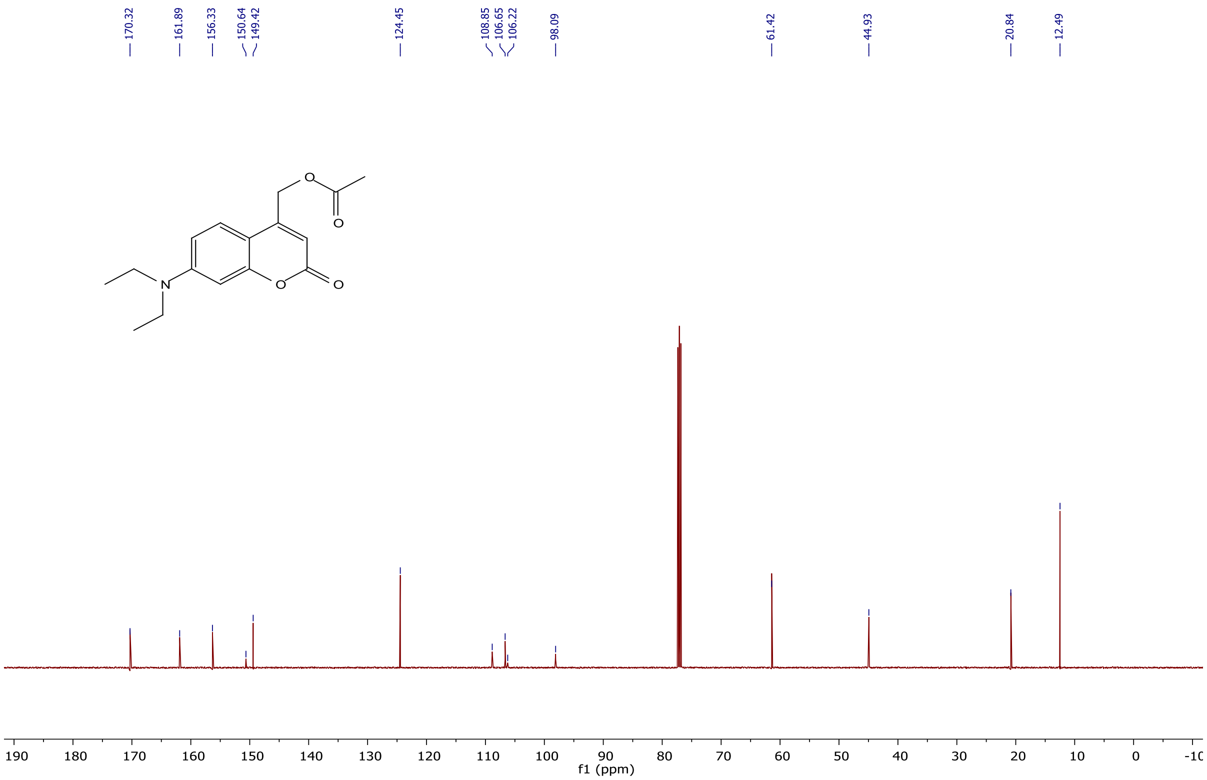

^{13}C NMR spectra of **compound 5**

(7-Diethylamino-4-methylacetoate-thiocoumarin) (6)

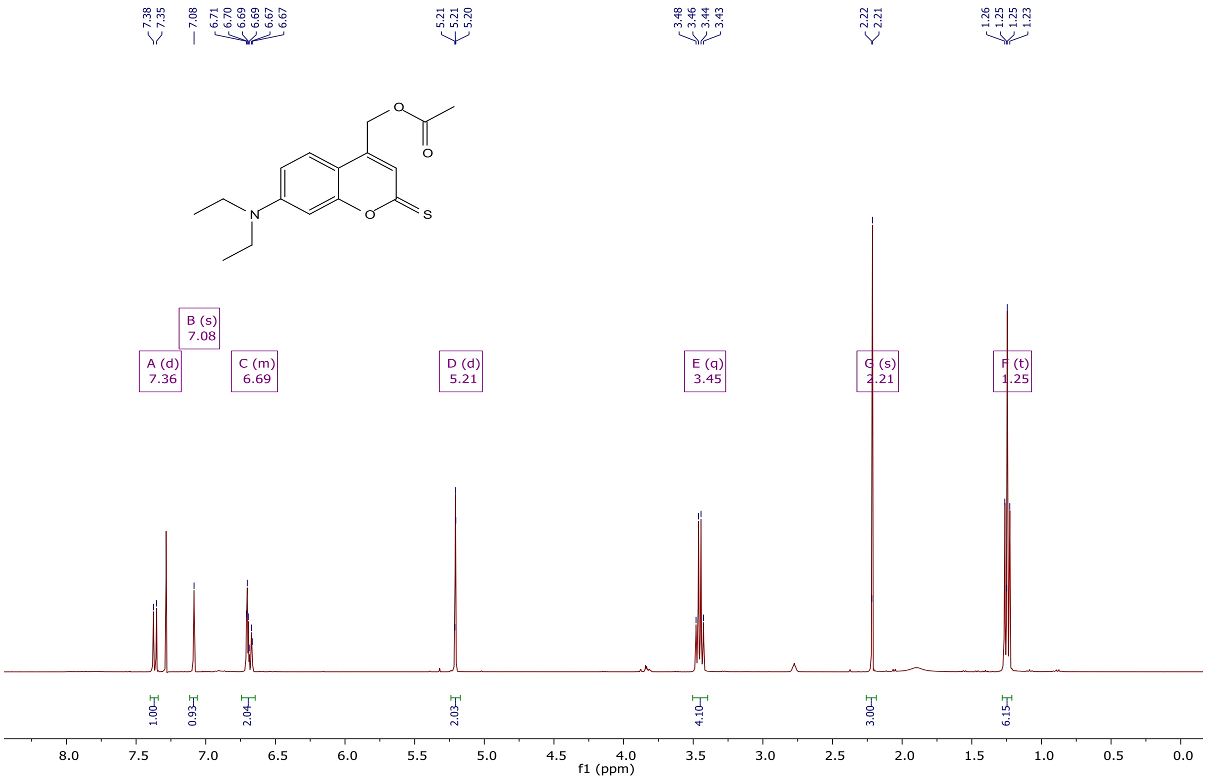

^{1}H NMR spectra of **compound 6**

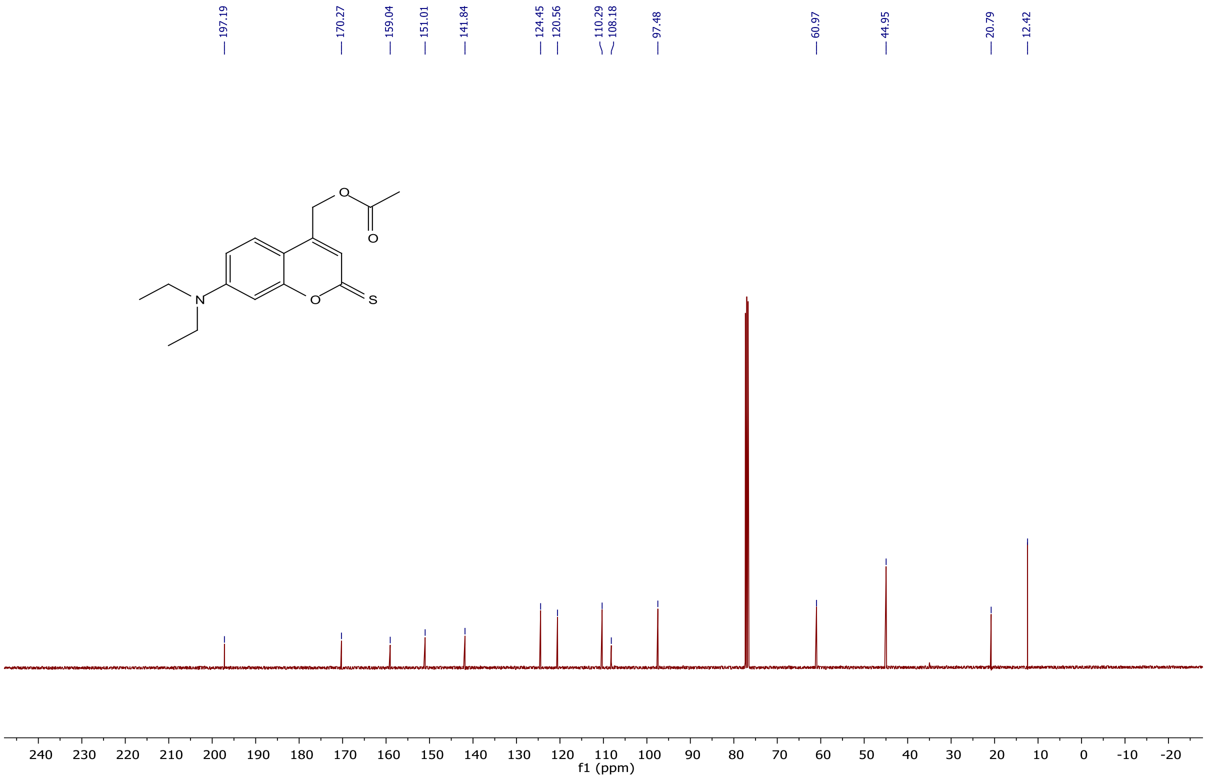

^{13}C NMR spectra of **compound 6**

(7-Diethylamino-4-hydroxymethyl-thiocoumarin) (7)

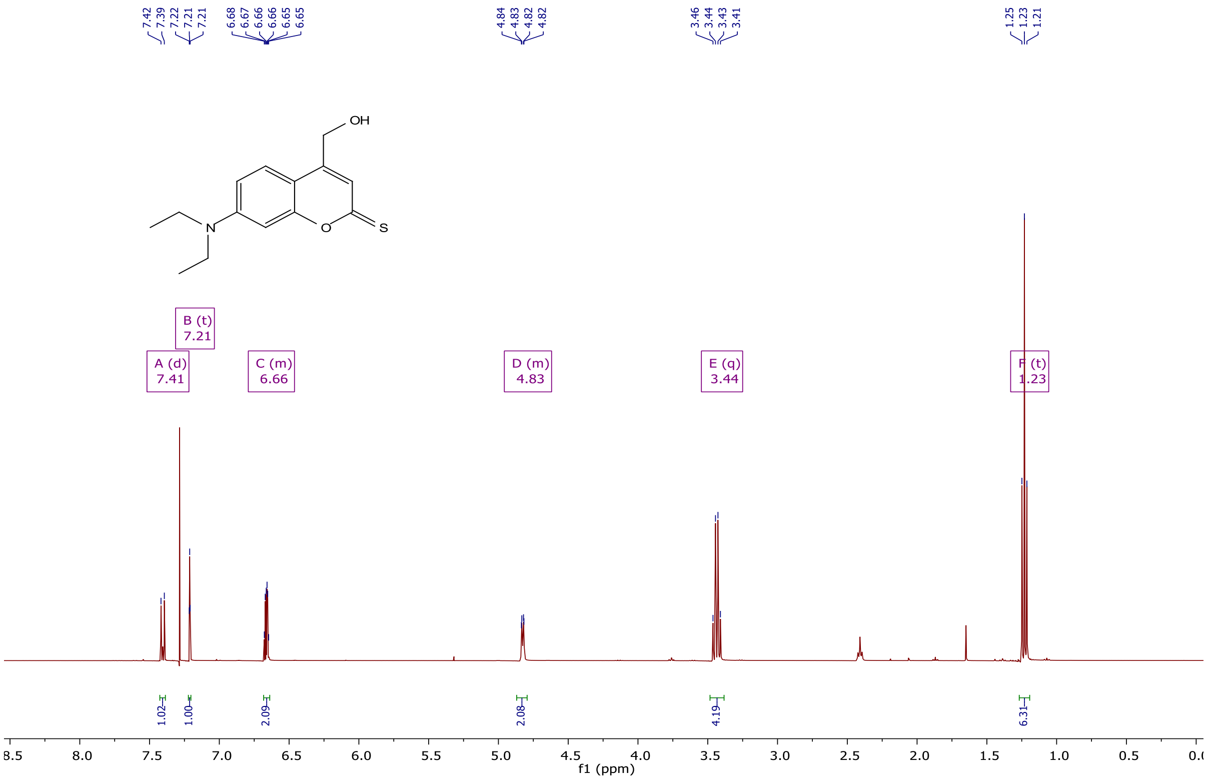

^{1}**H** NMR spectra of **compound 7**

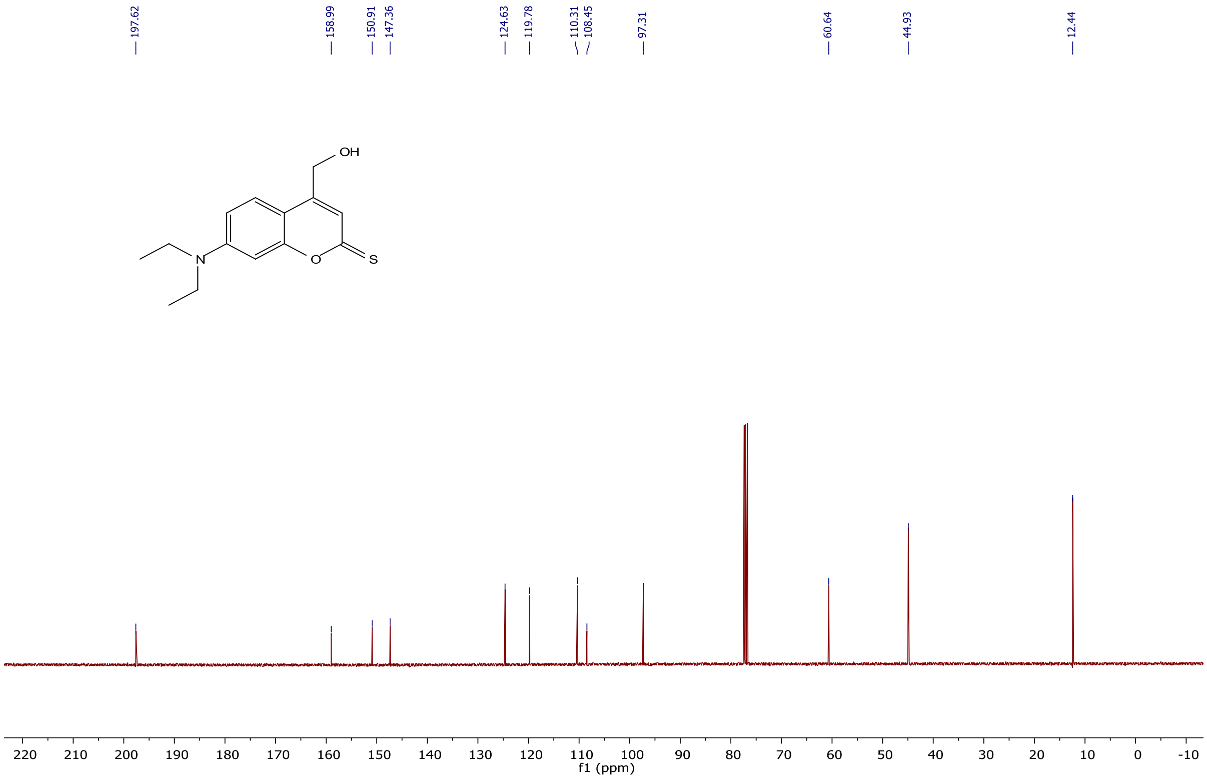

^{13}**C** NMR spectra of **compound 7**

(*i*Pr₂N)₂P(OFm)) (8)

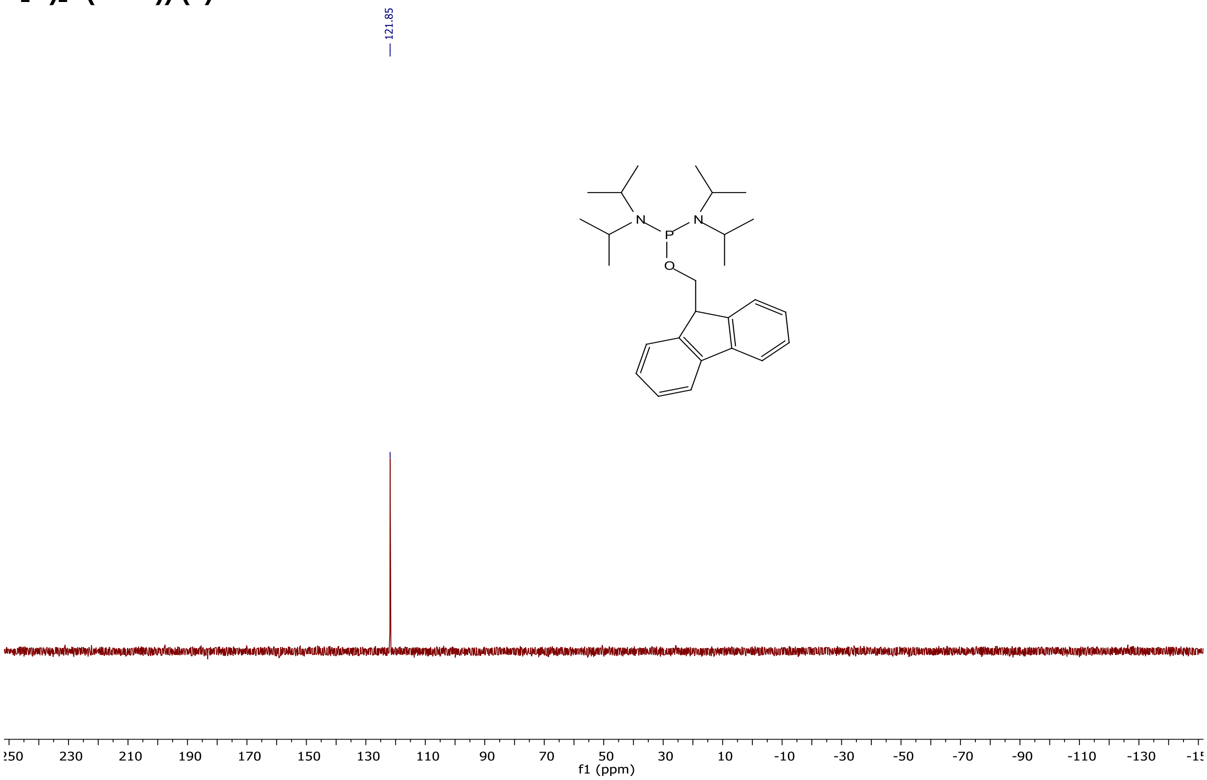

^{31}**P** NMR spectra of **compound 8**

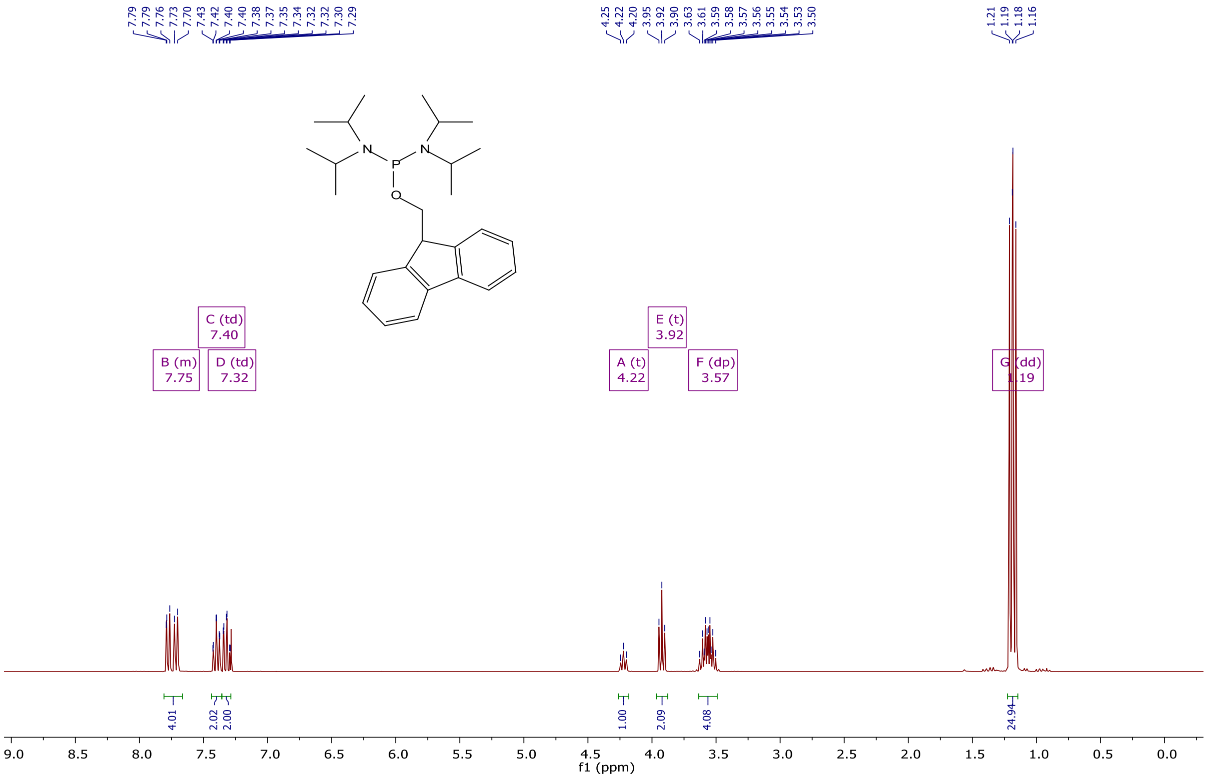

^{1}**H** NMR spectra of **compound 8**

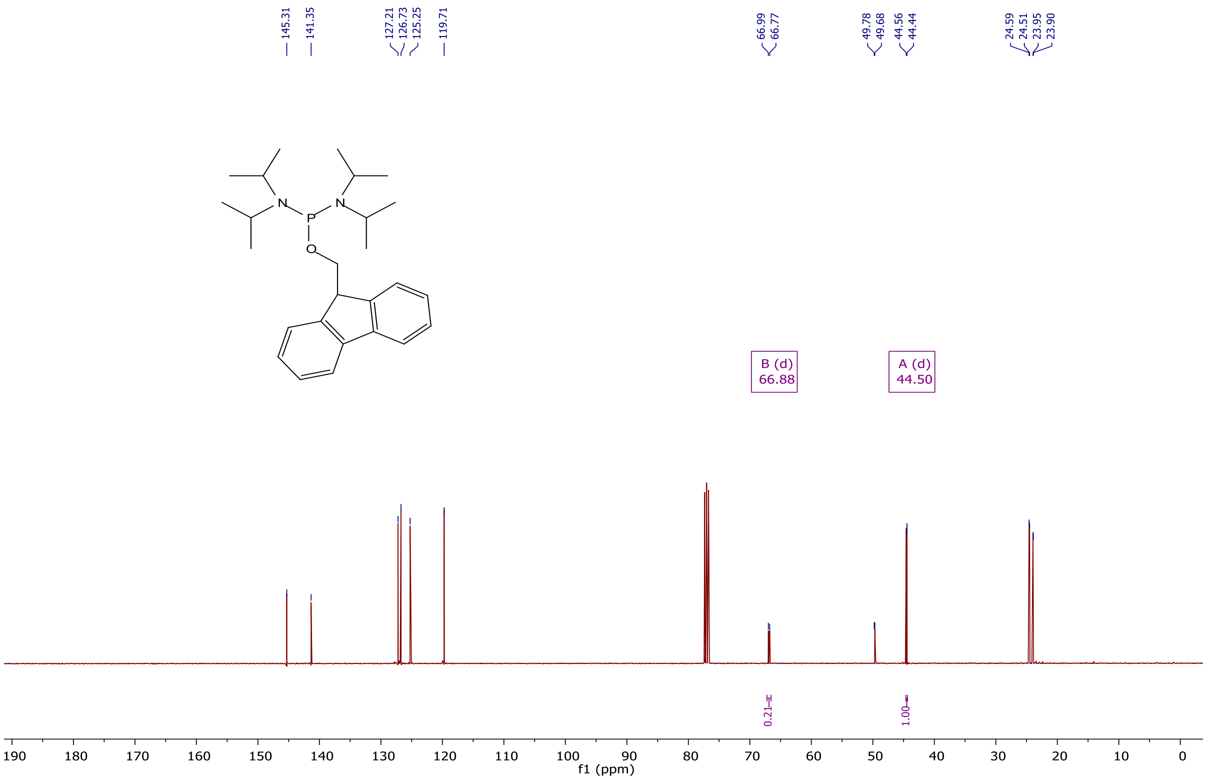

^{13}**C** NMR spectra of **Compound 8**

((*i*Pr$_2$N)(FmO)P-Othio-DEACM) (9)

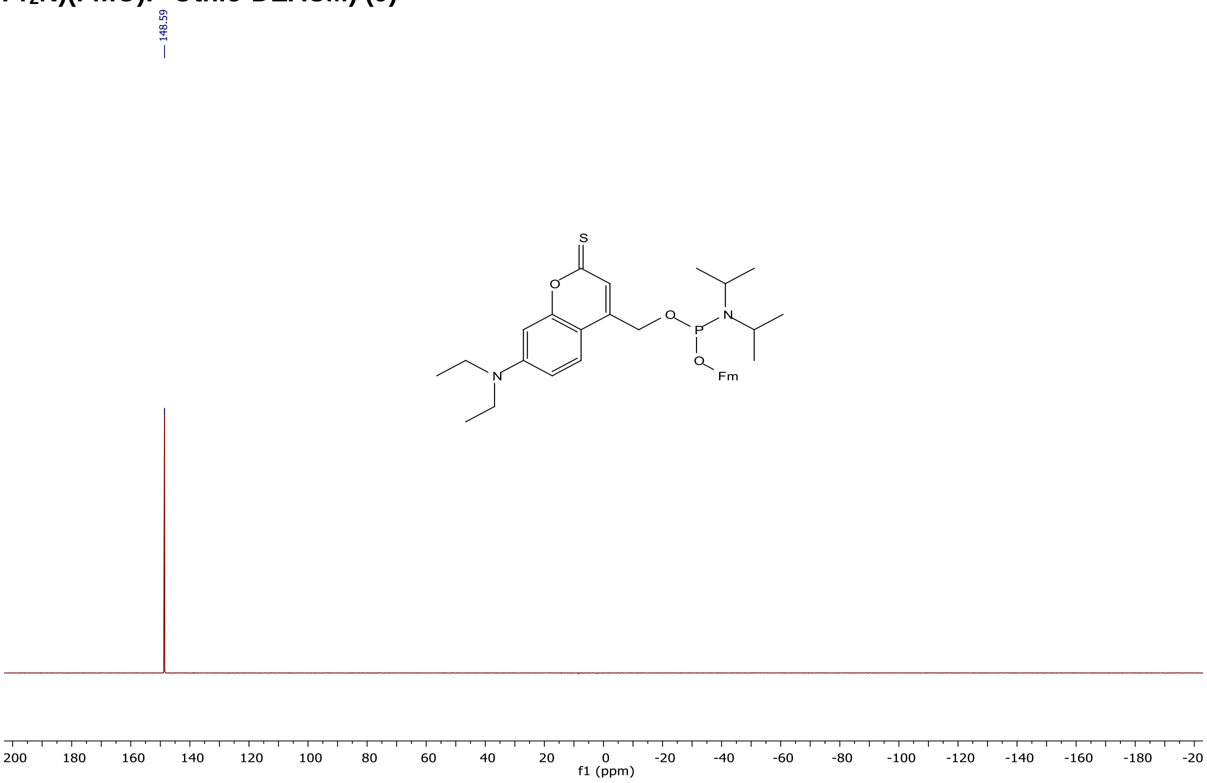

^{31}**P** NMR spectra of **compound 9**

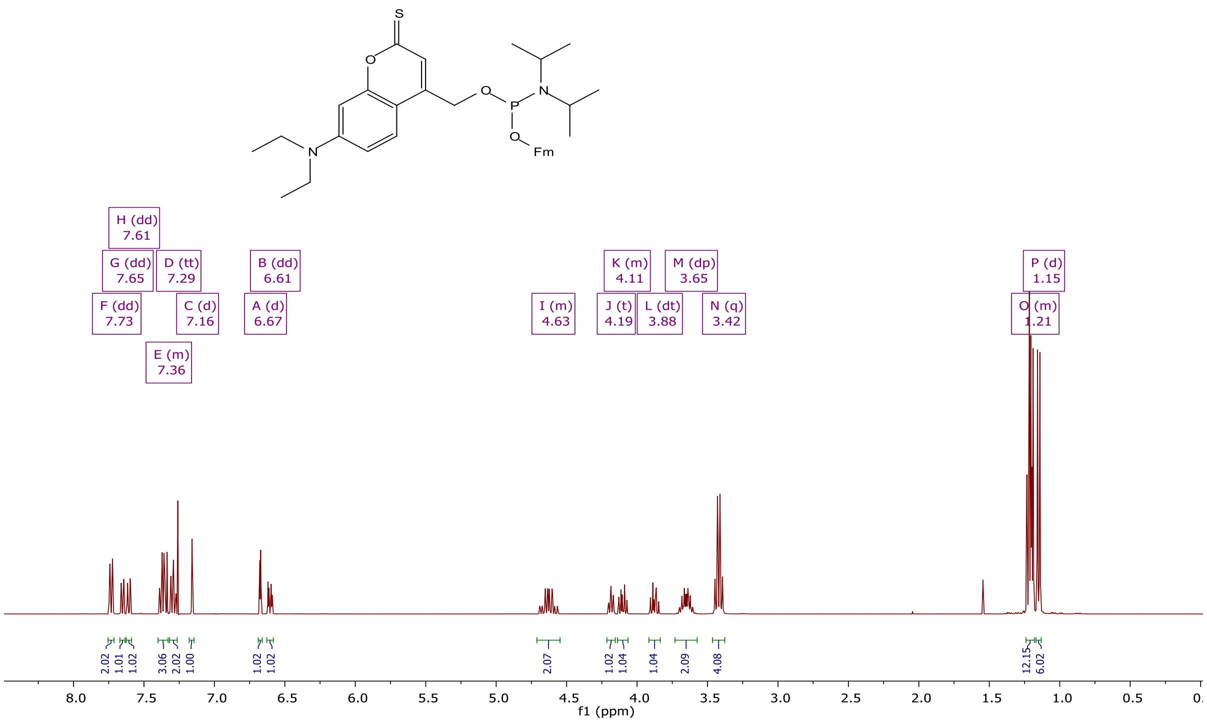

^{1}**H** NMR spectra of **compound 9**

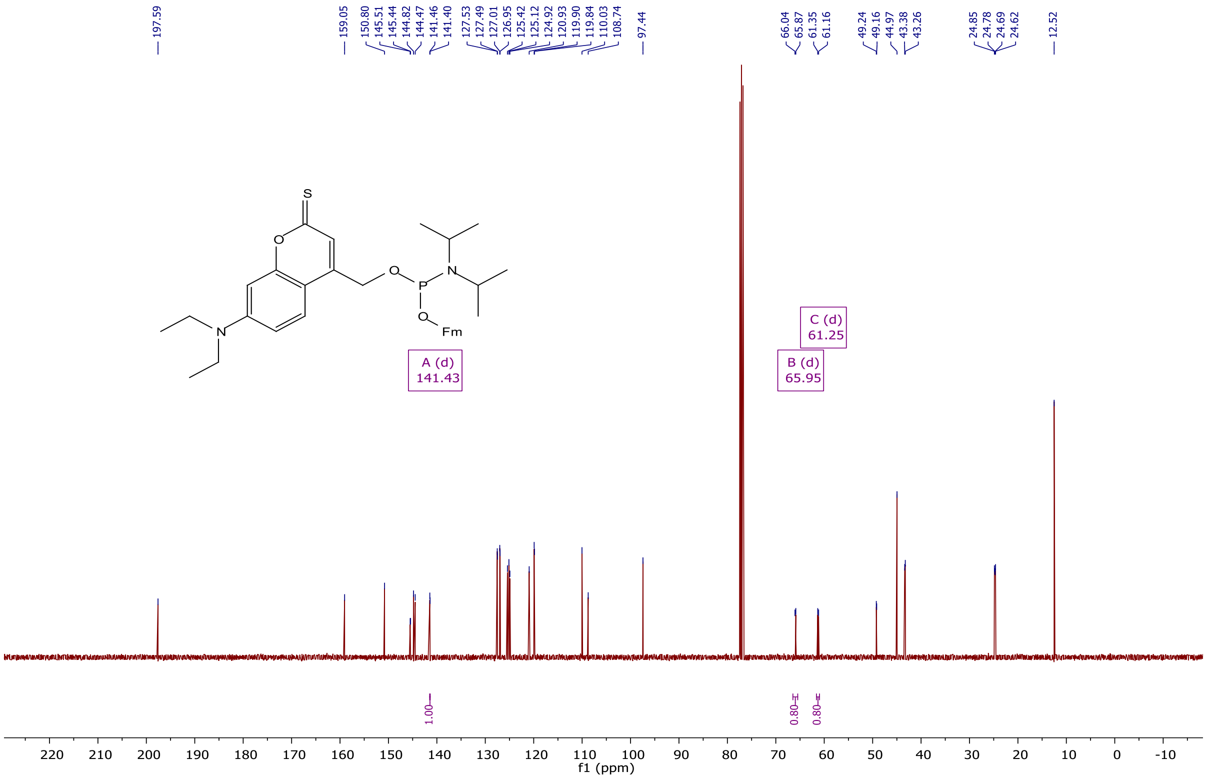

^{13}**C** NMR spectra of **compound 9**

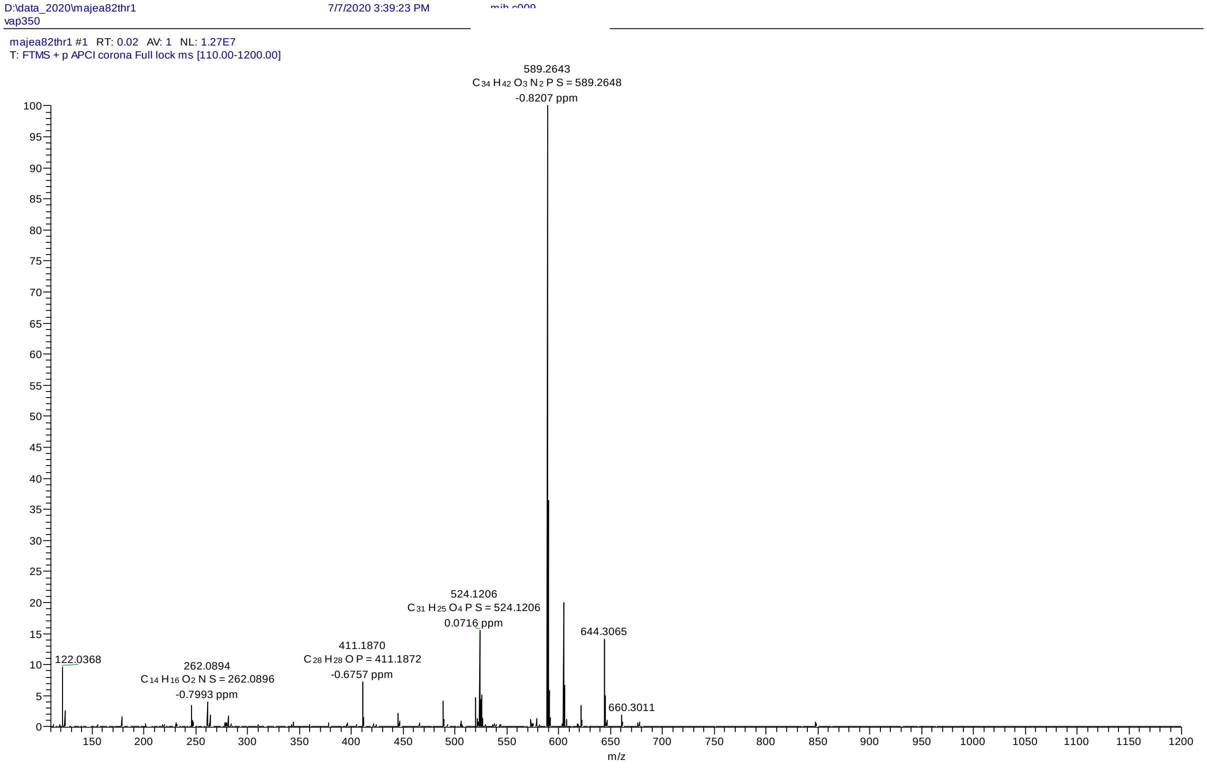

MASS spectra of **Compound 9**

DEACM-ADP

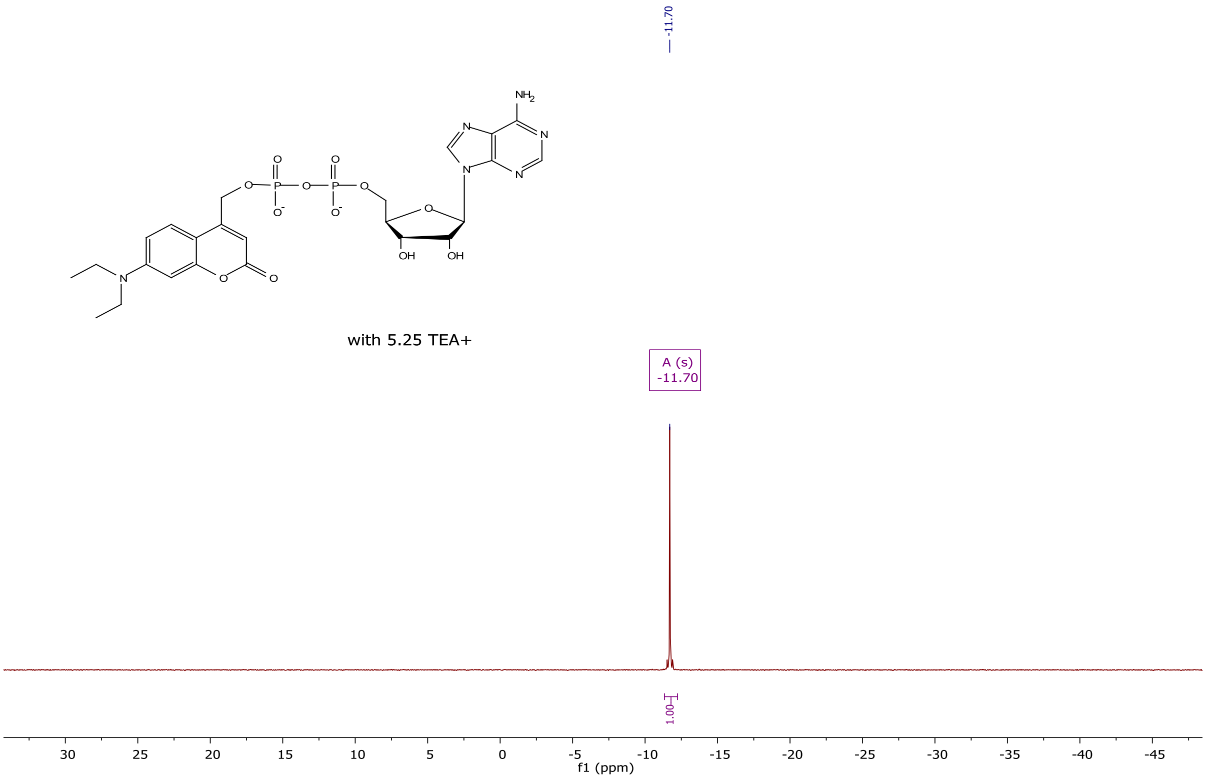

^{31}P NMR spectra of **DEACM-caged ADP**

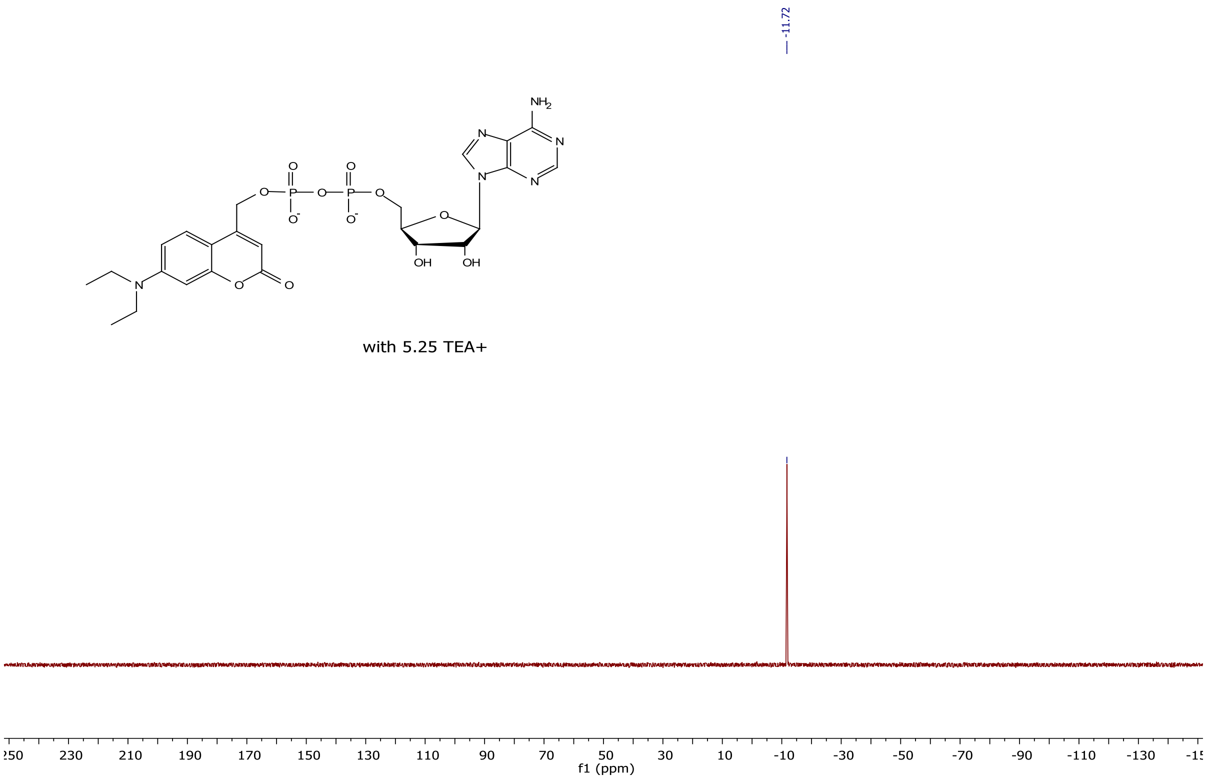

^{31}P coupled NMR spectra of **DEACM-caged ADP**

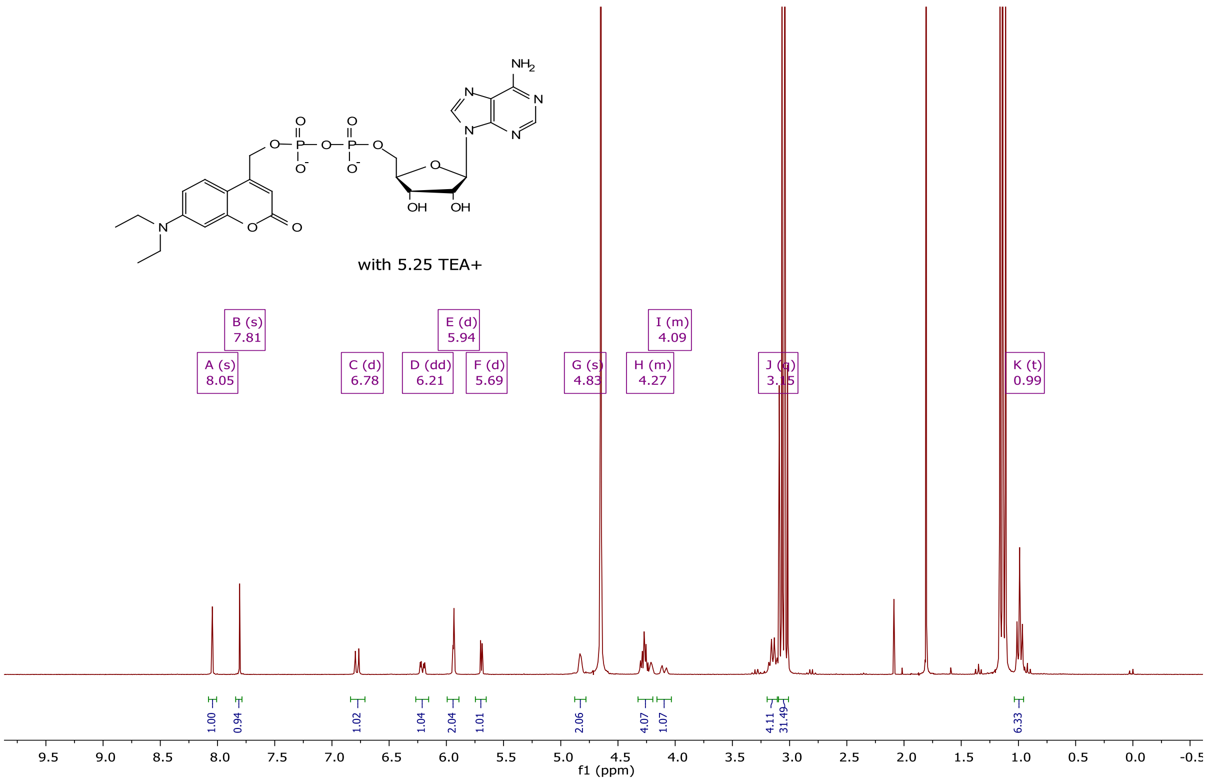

^{1}H NMR spectra of **DEACM-caged ADP**

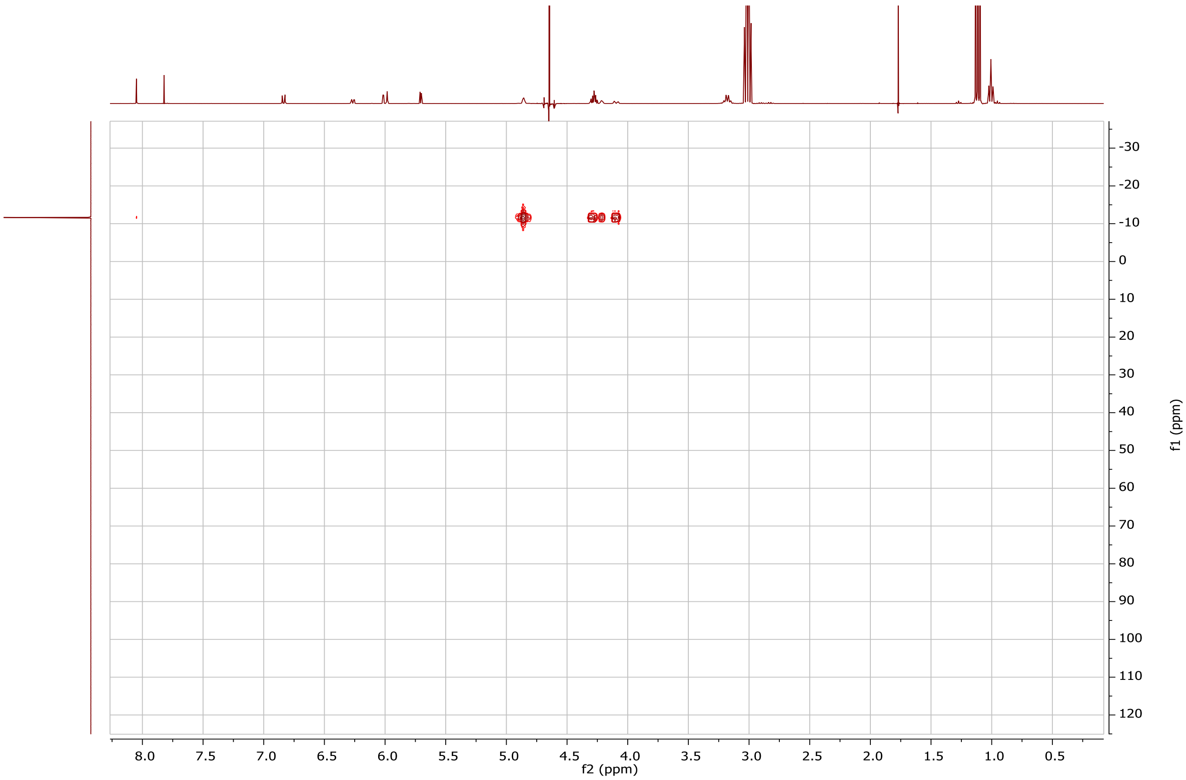

HMBC-^{31}P spectra of **DEACM-caged ADP**

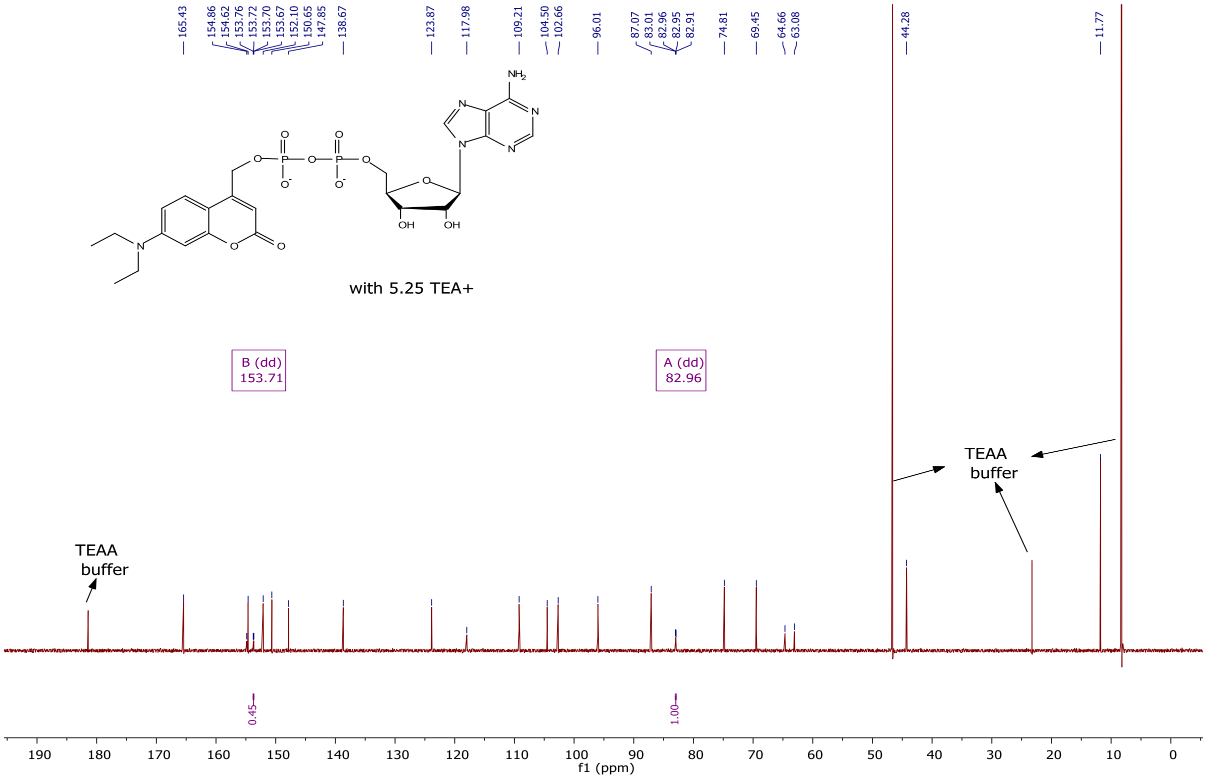

^{13}C NMR spectra of **DEACM-caged ADP**

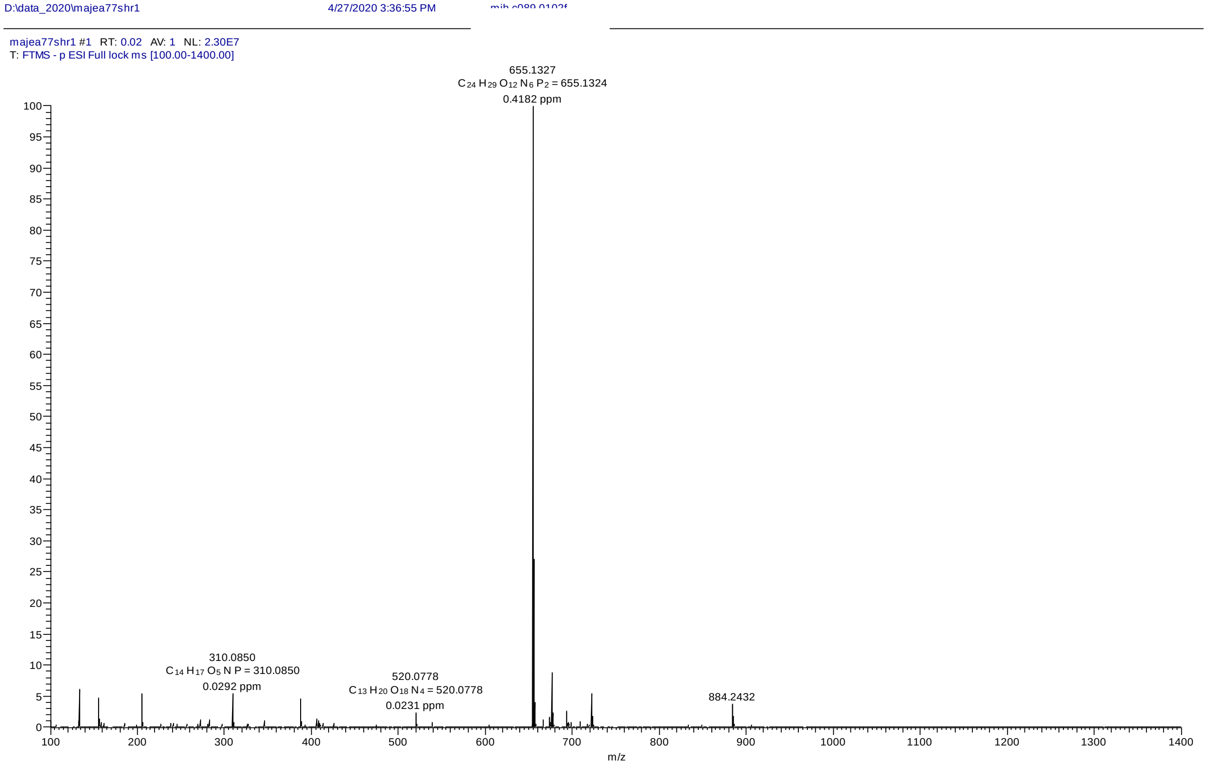

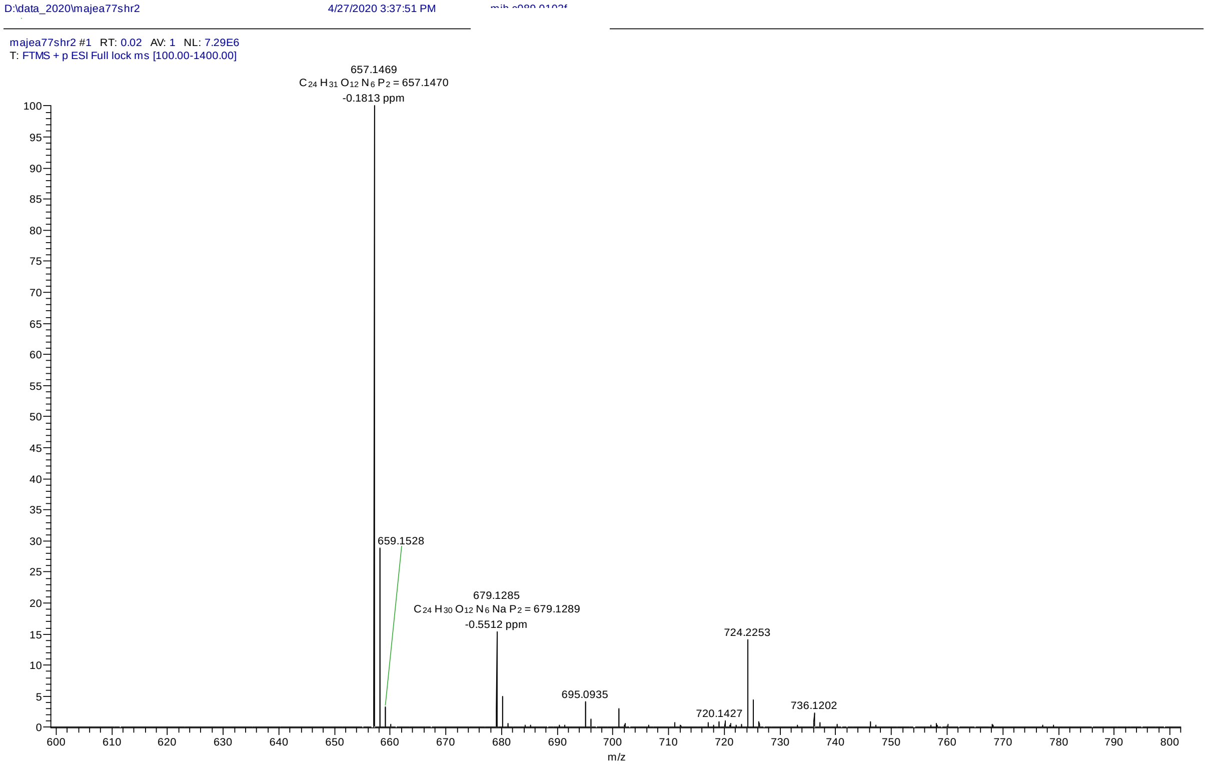

MASS spectra of **DEACM-caged ADP**

Thio-DEACM- ADP

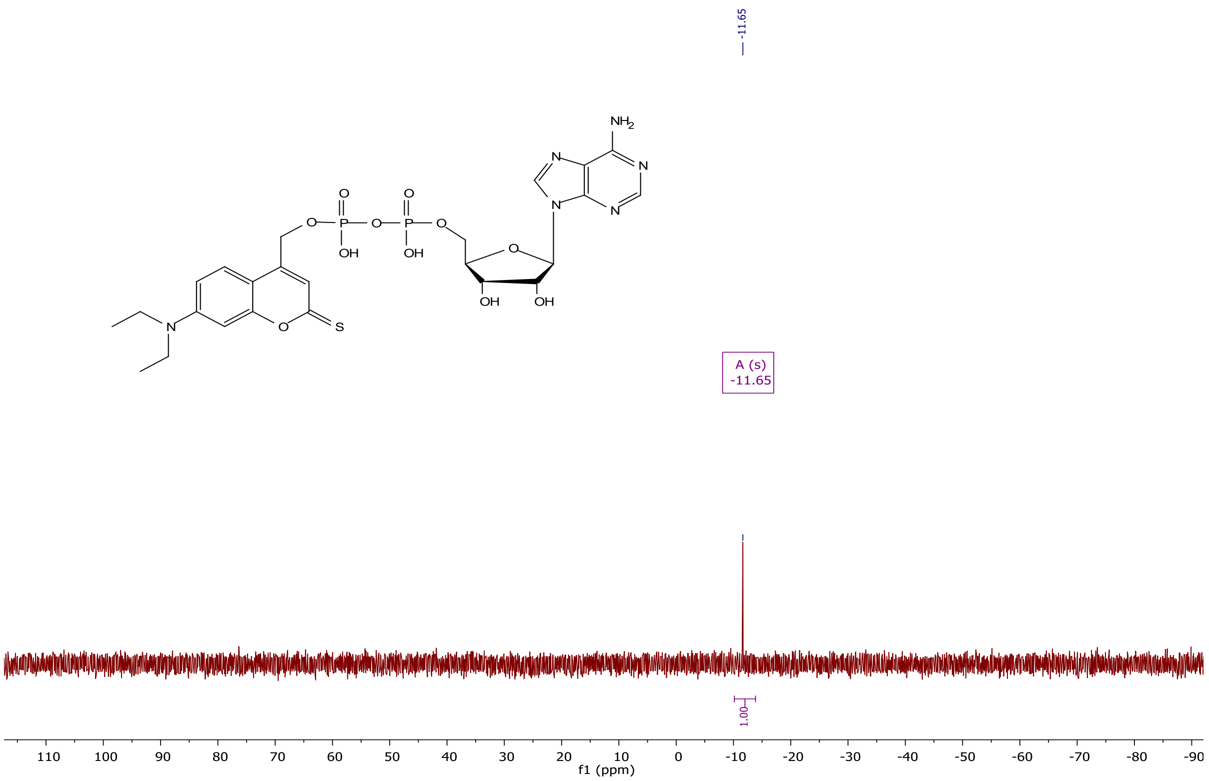

^{31}P NMR spectra of **Thio-DEACM-caged ADP**

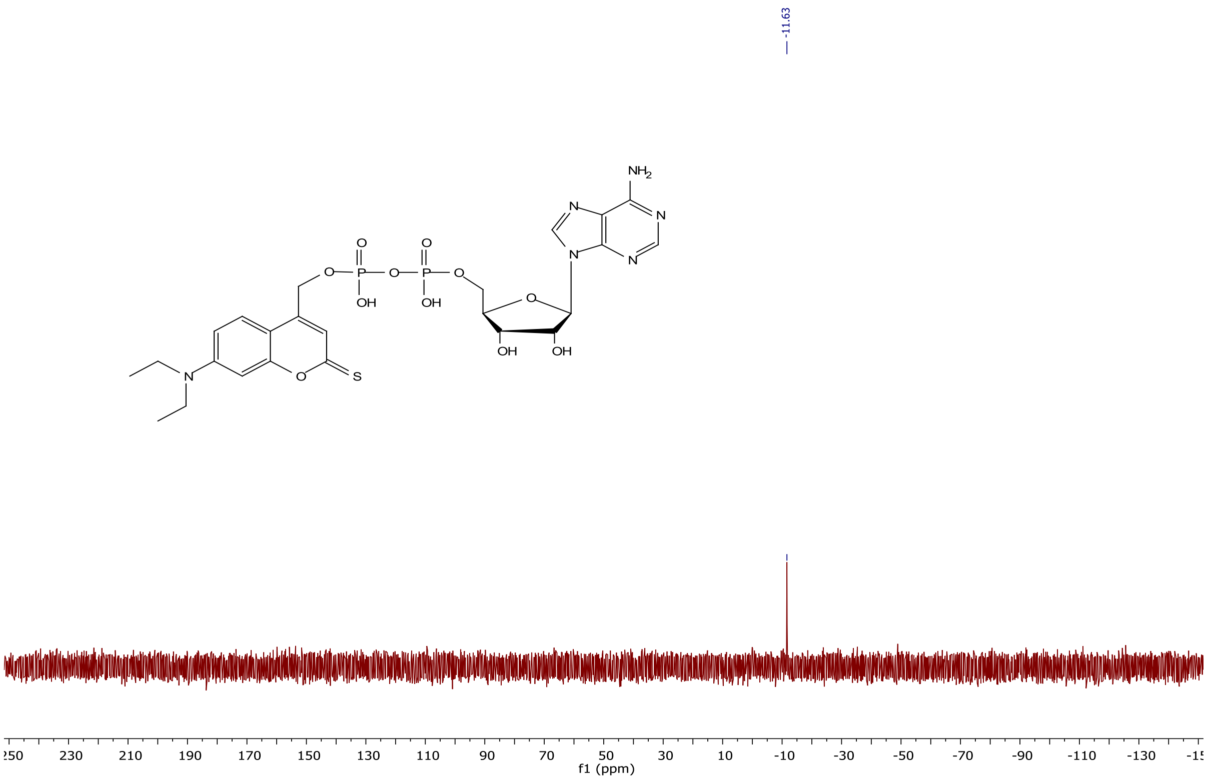

^{31}P coupled NMR spectra of **Thio-DEACM-caged ADP**

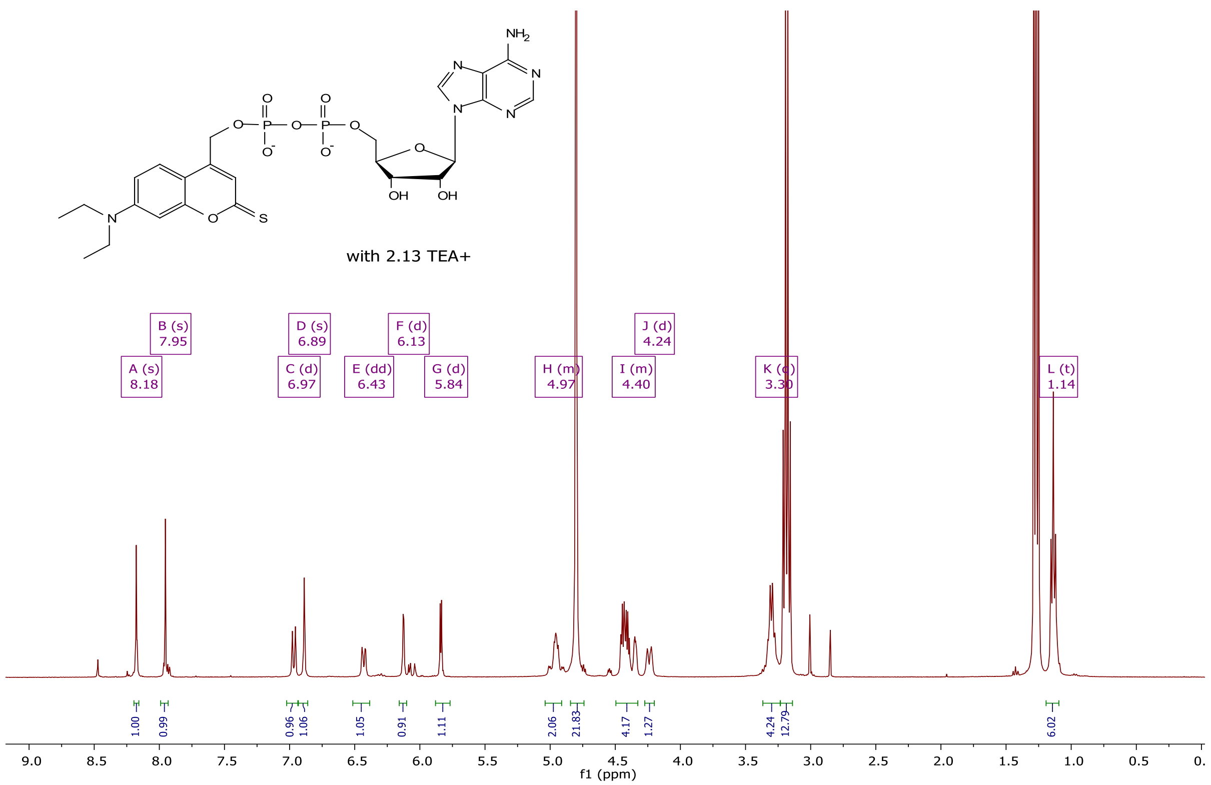

^{1}H NMR spectra of **Thio-DEACM-caged ADP**

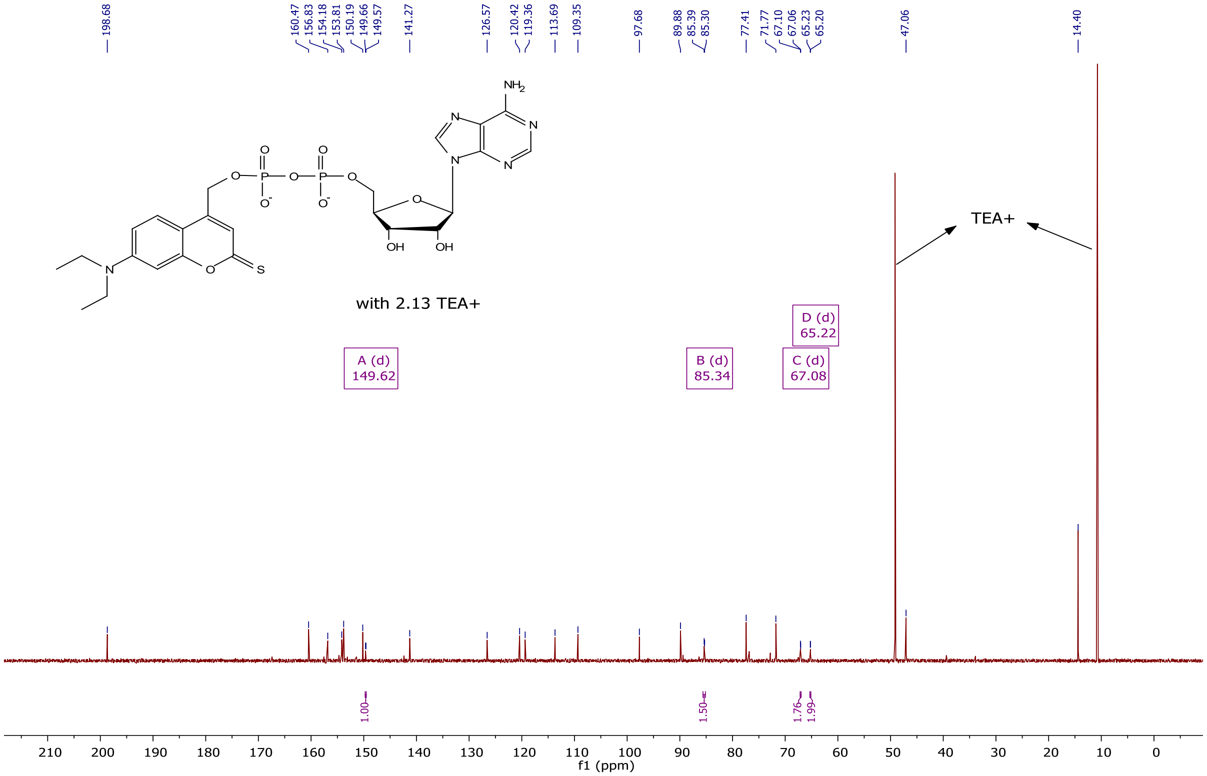

^{13}C NMR spectra of **Thio-DEACM-caged ADP**

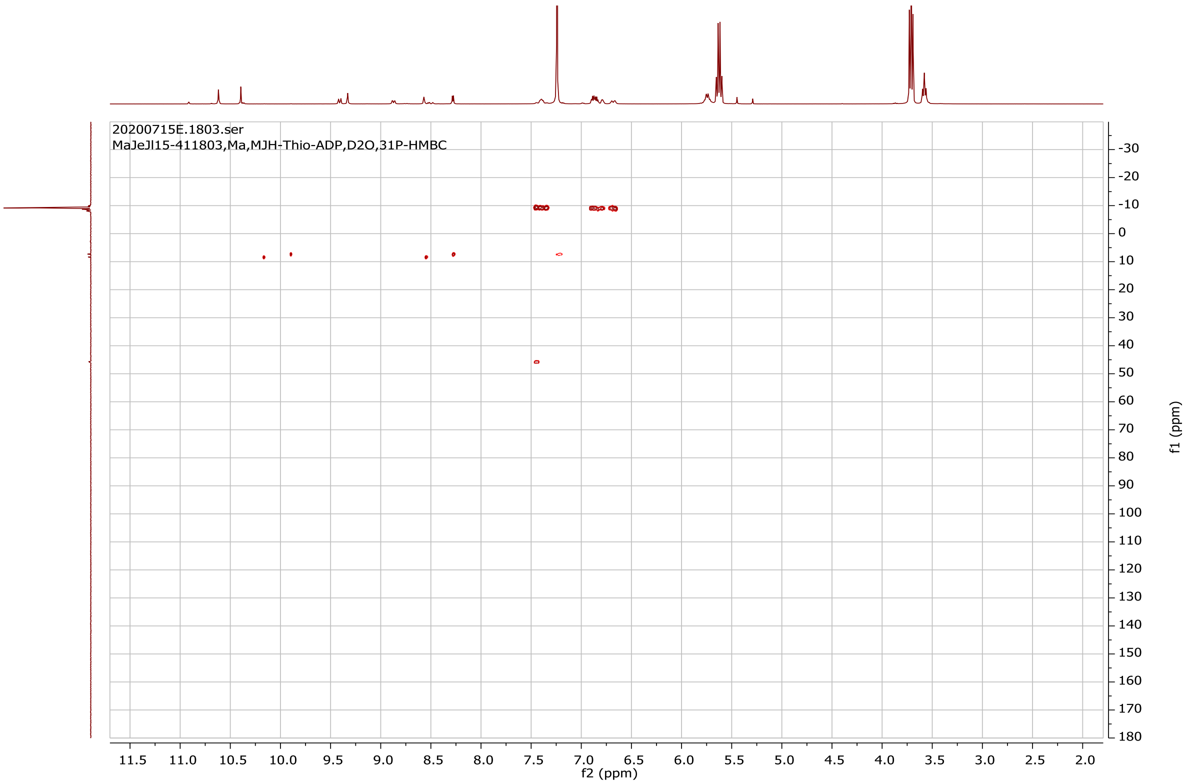

HMBC-^{31}P spectra of **Thio-DEACM-caged ADP**

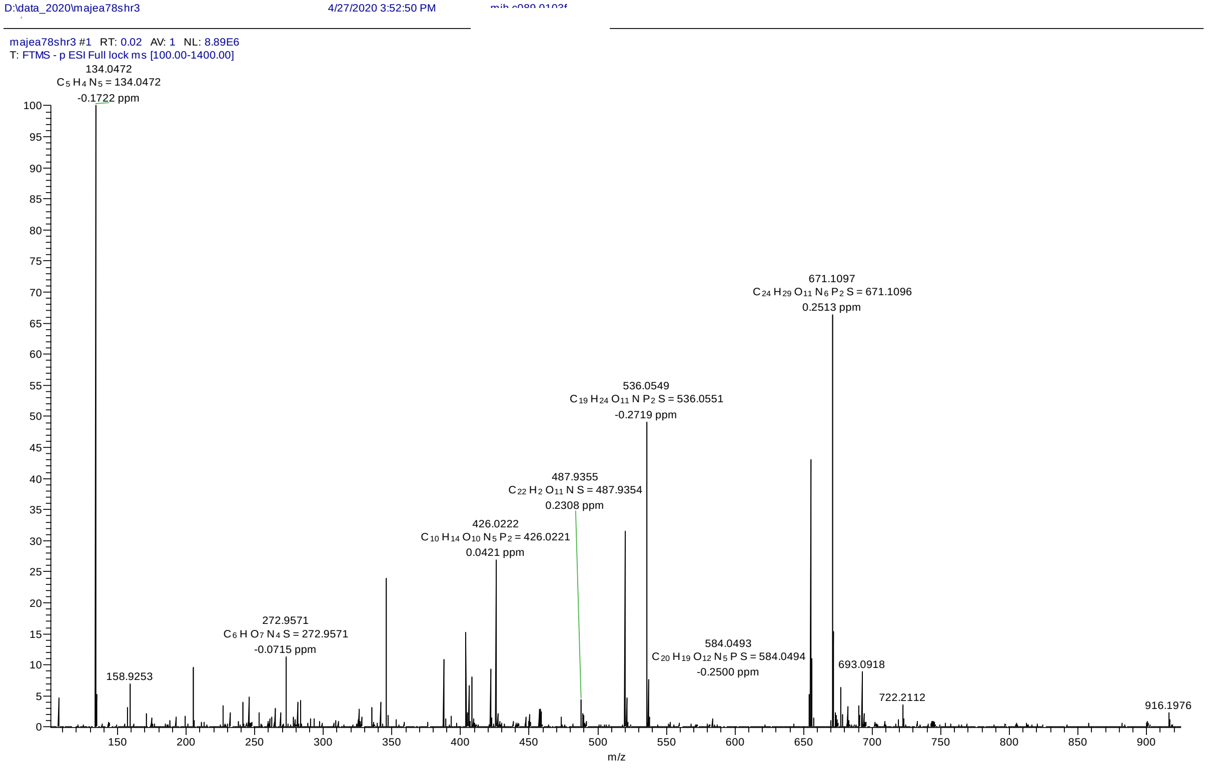

MASS spectra of **Thio-DEACM-caged ADP**

DEACM-ATP

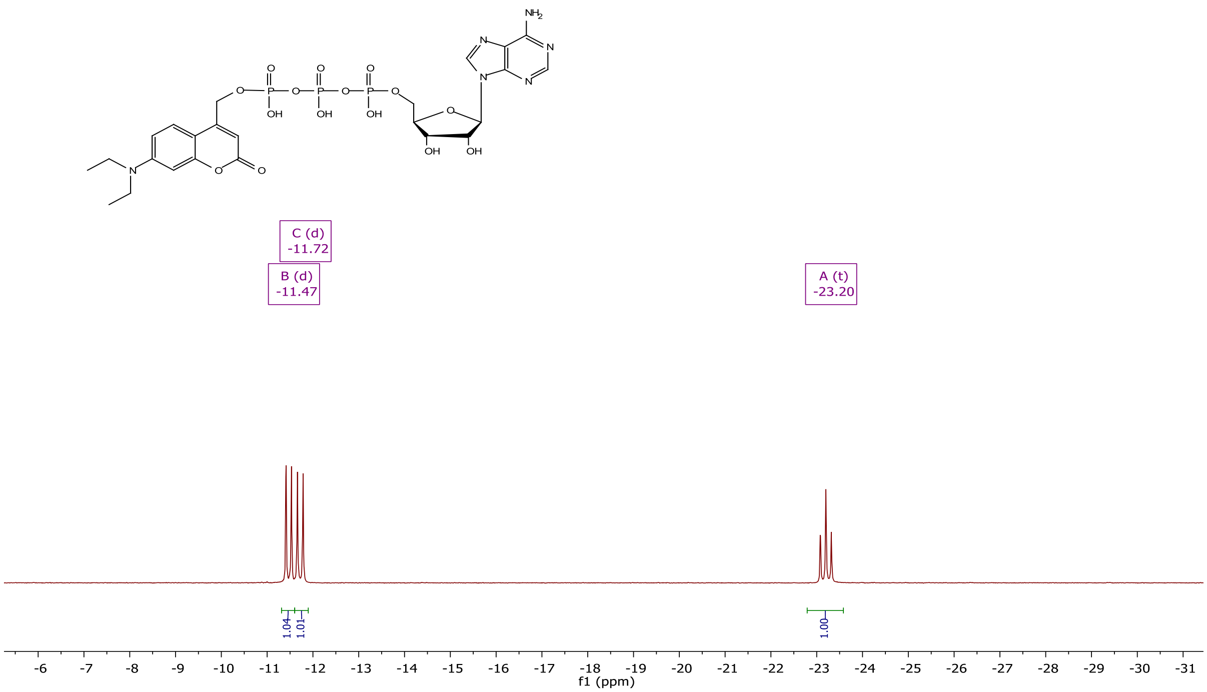

^{31}P NMR spectra of **DEACM-caged ATP**

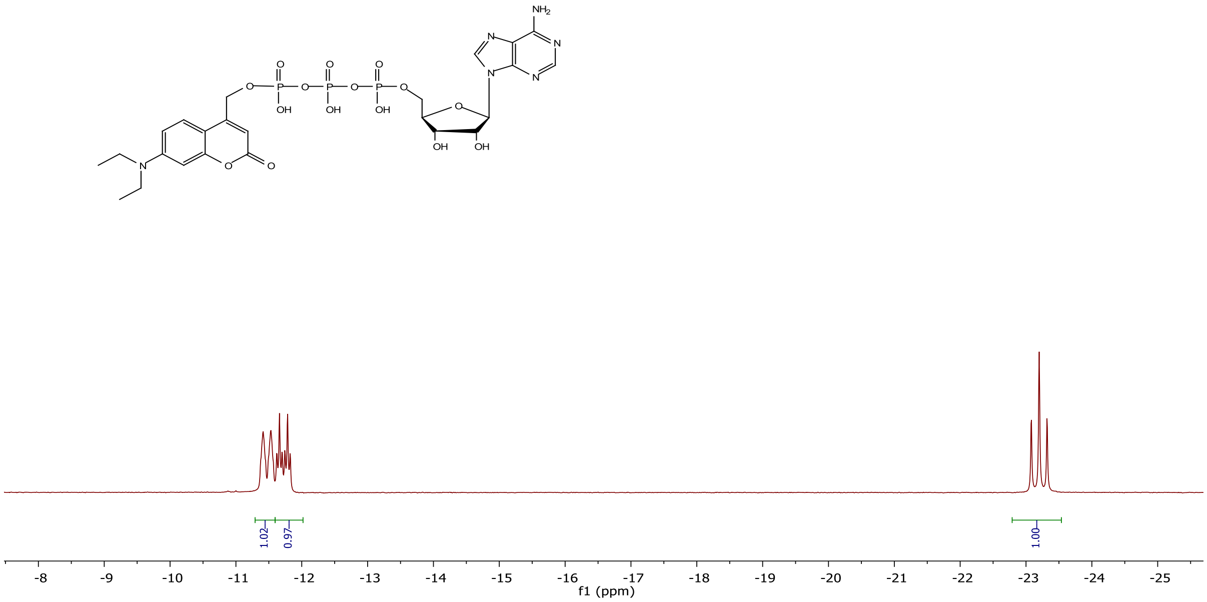

^{31}P coupled NMR spectra of **DEACM-caged ATP**

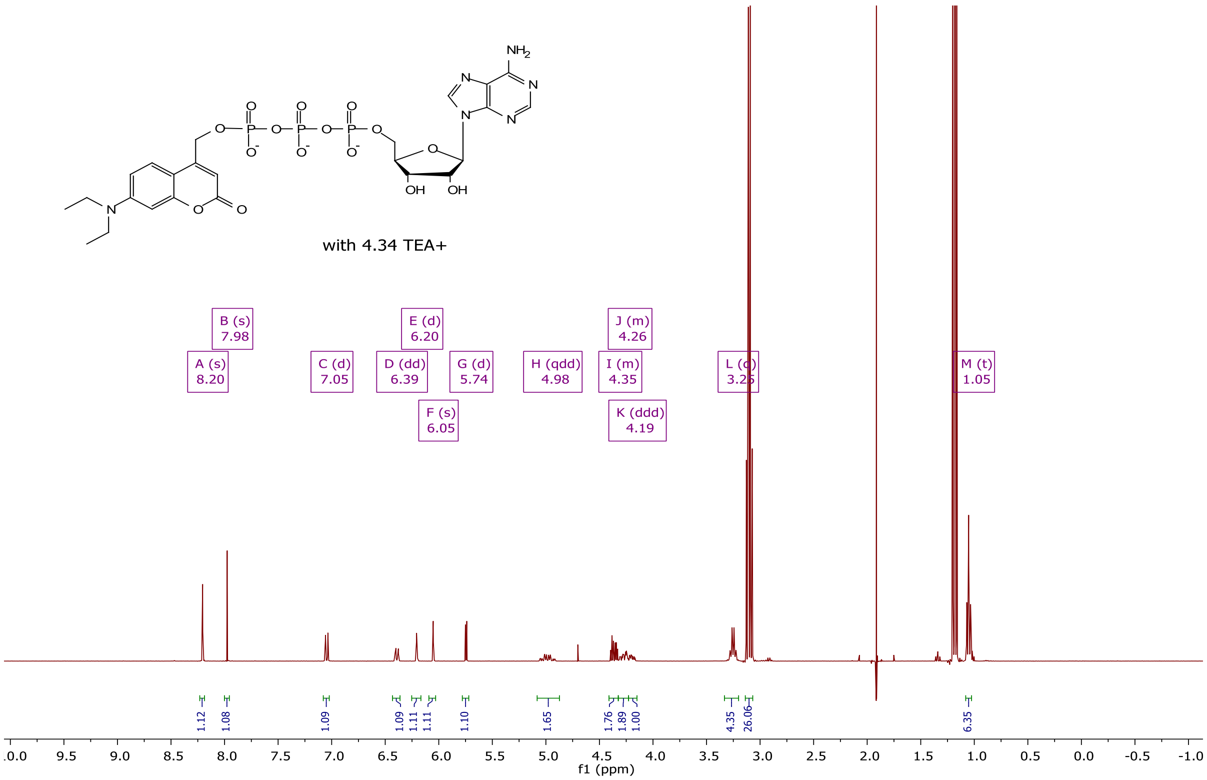

^{1}H NMR spectra of **DEACM-caged ATP**

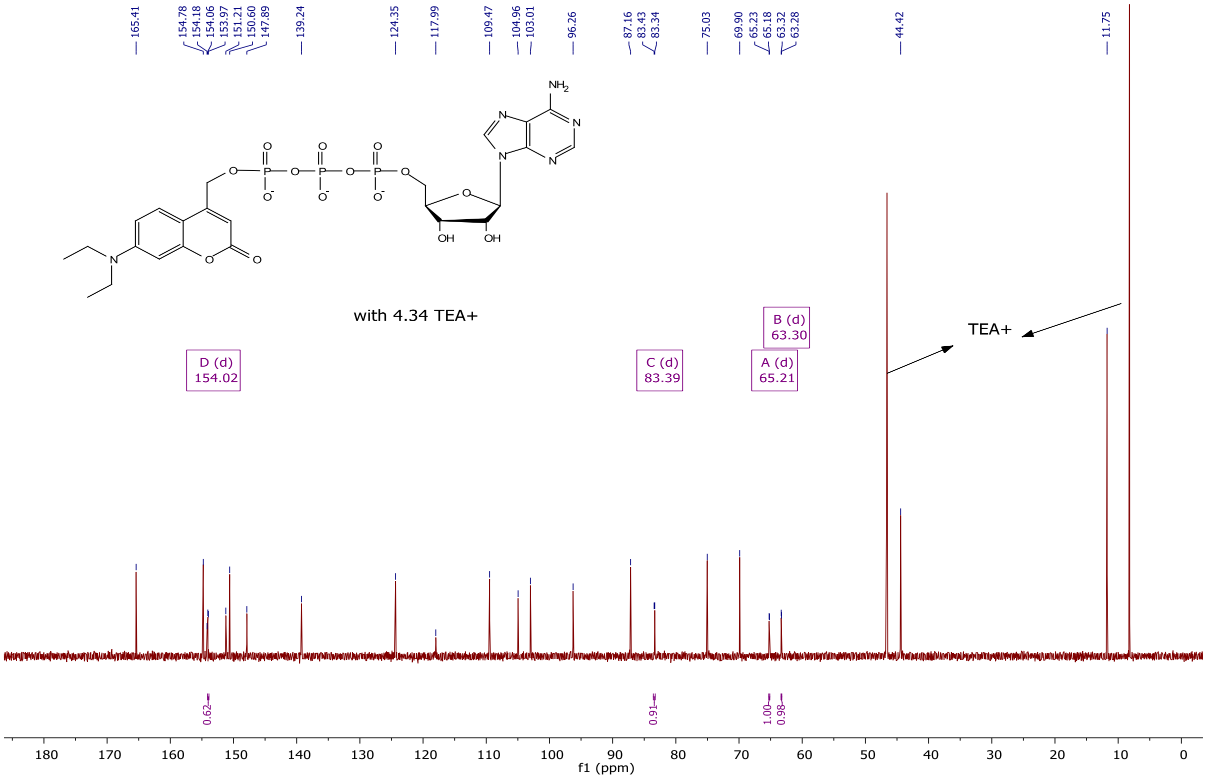

^{13}C NMR spectra of **DEACM-caged ATP**

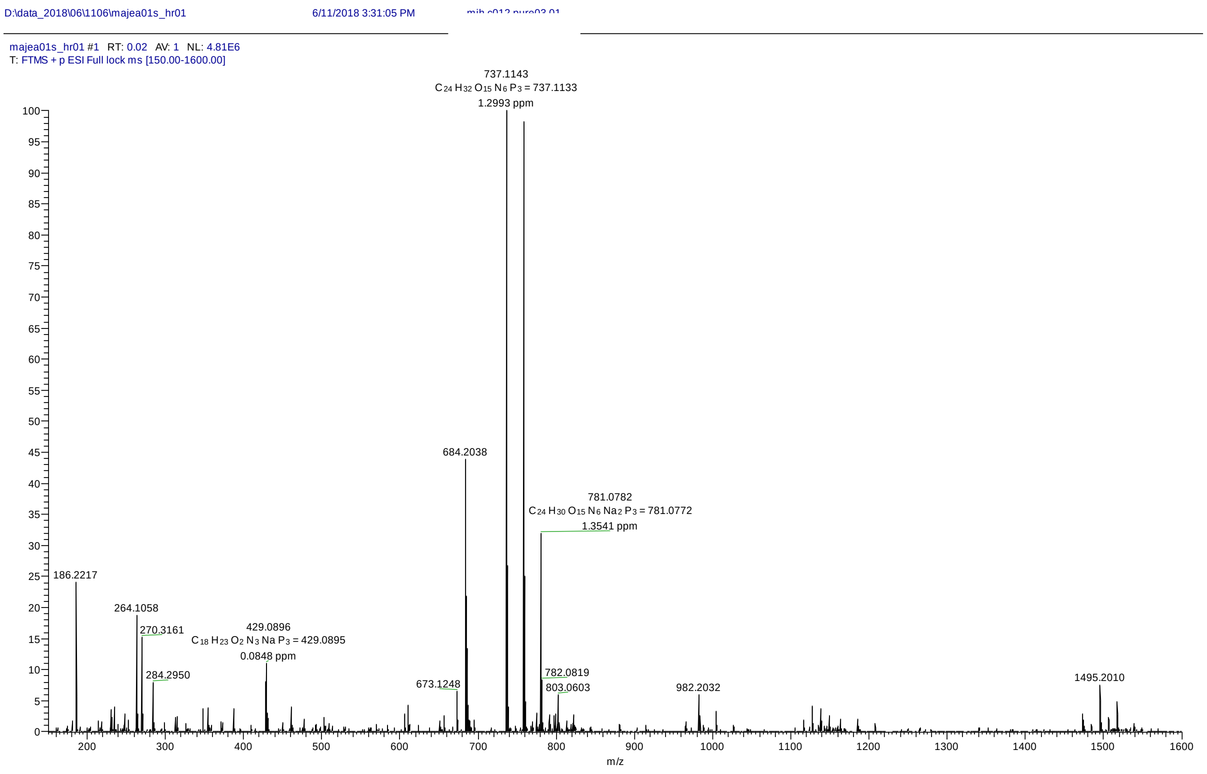

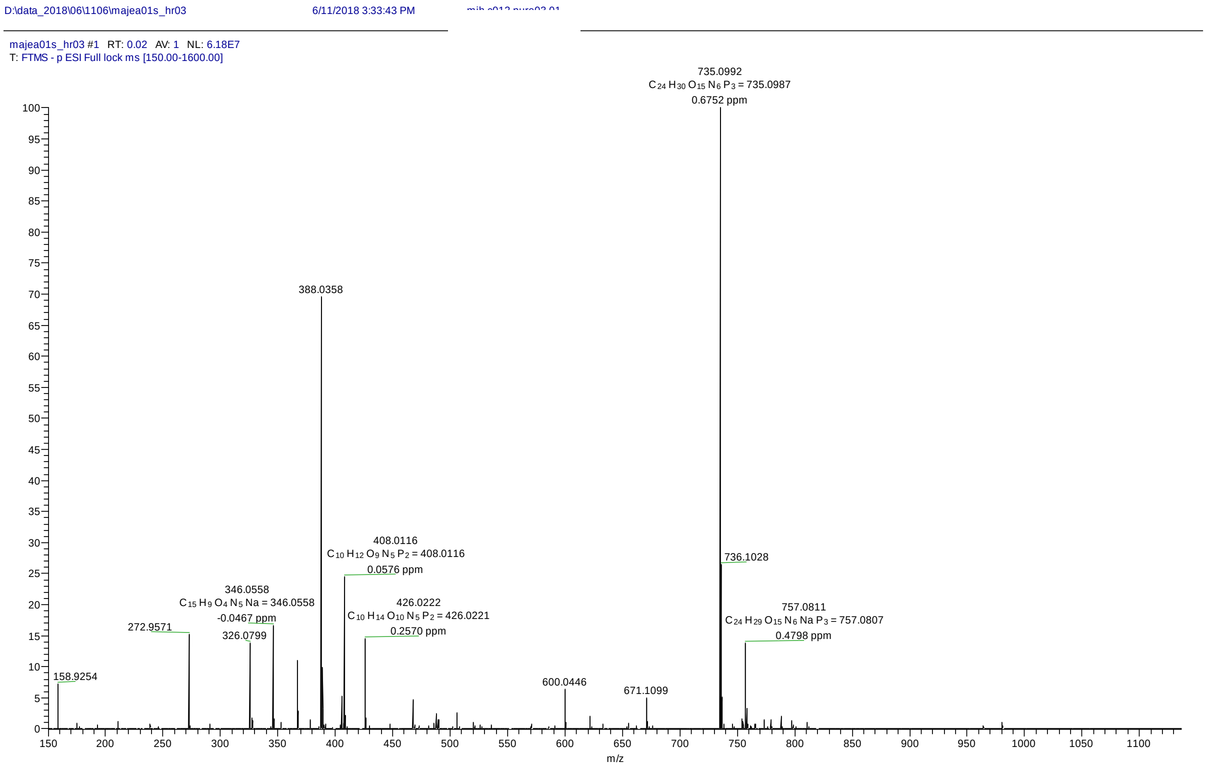

MASS spectra of **DEACM-caged ATP**

Thio-DEACM-ATP

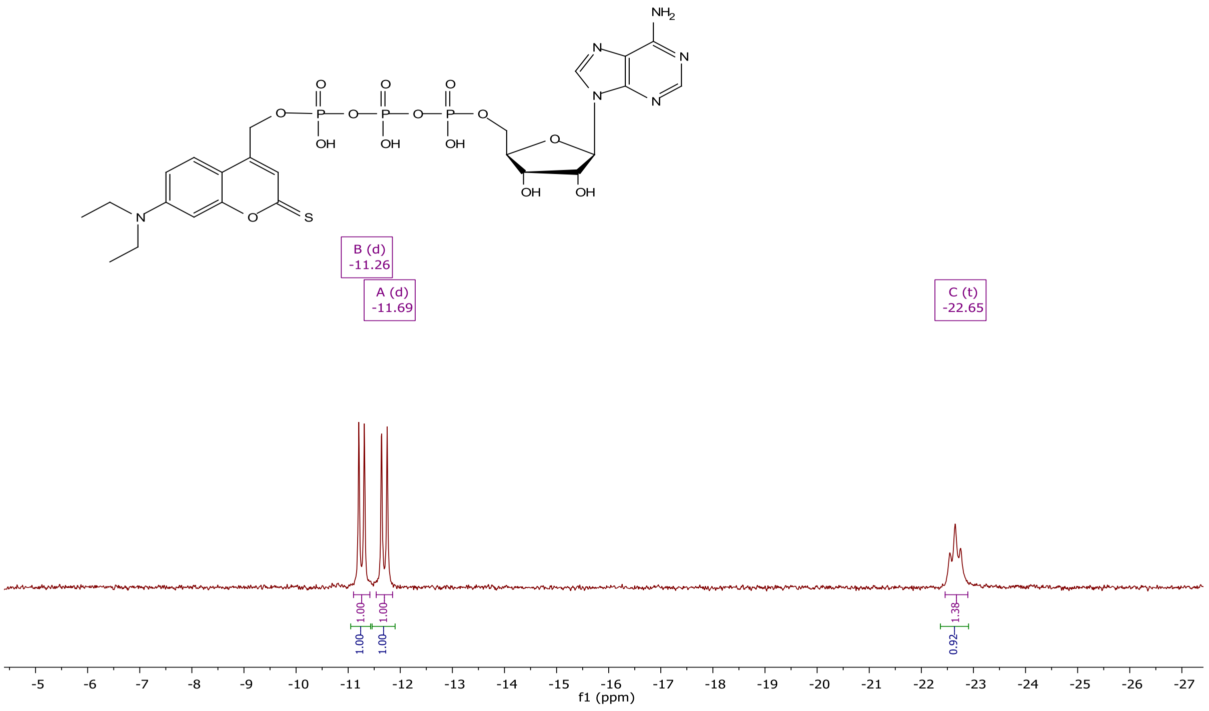

^{31}**P** NMR spectra of **Thio-DEACM-caged ATP**

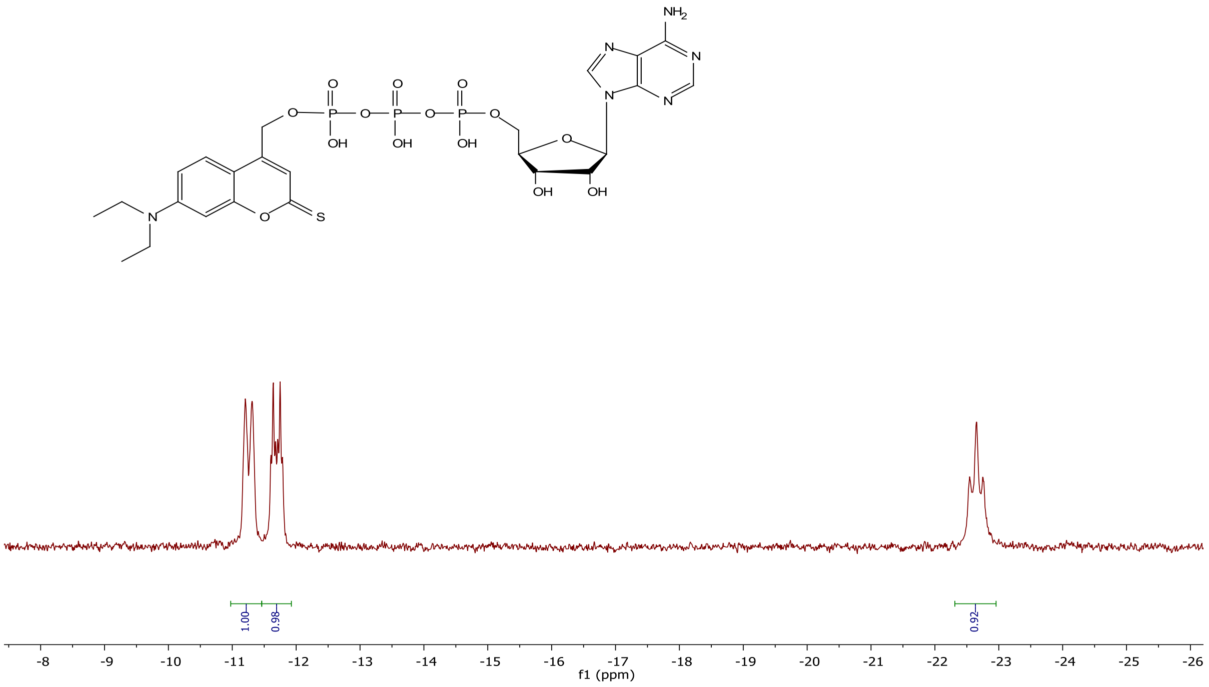

^{31}**P coupled** NMR spectra of **Thio-DEACM-caged ATP**

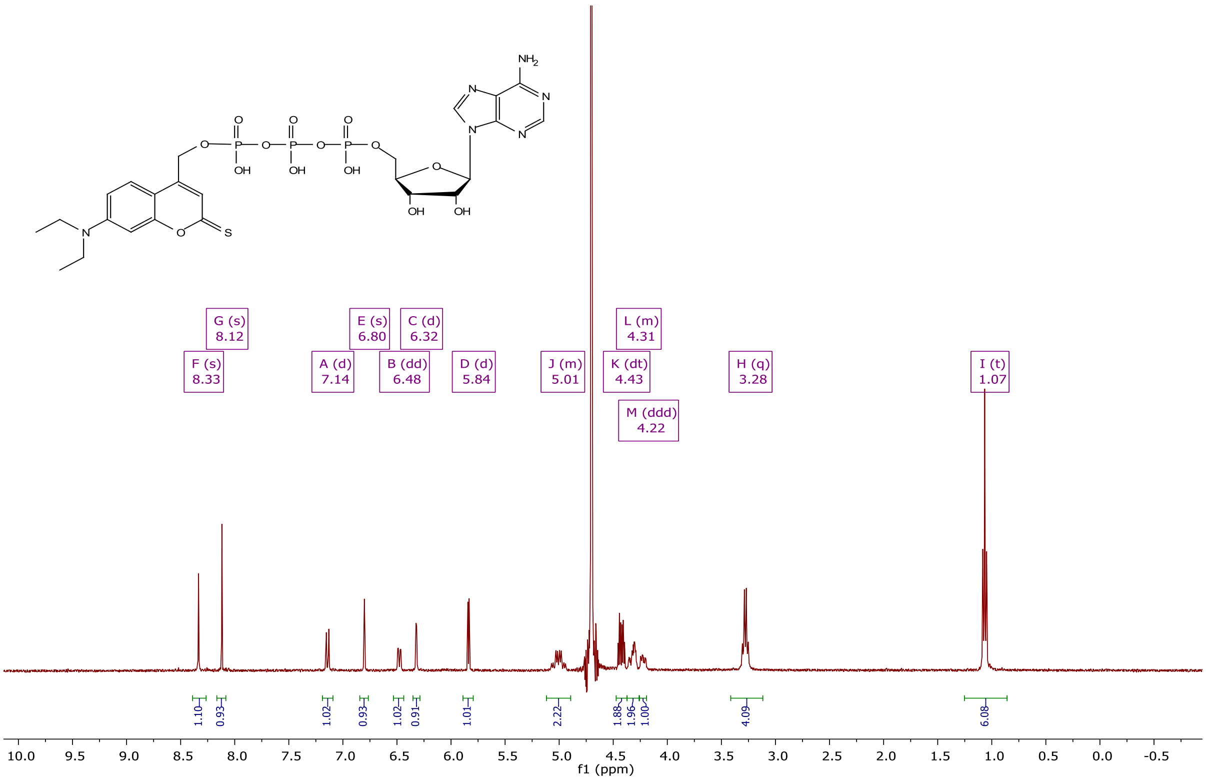

^{1}H NMR spectra of **Thio-DEACM-caged ATP**

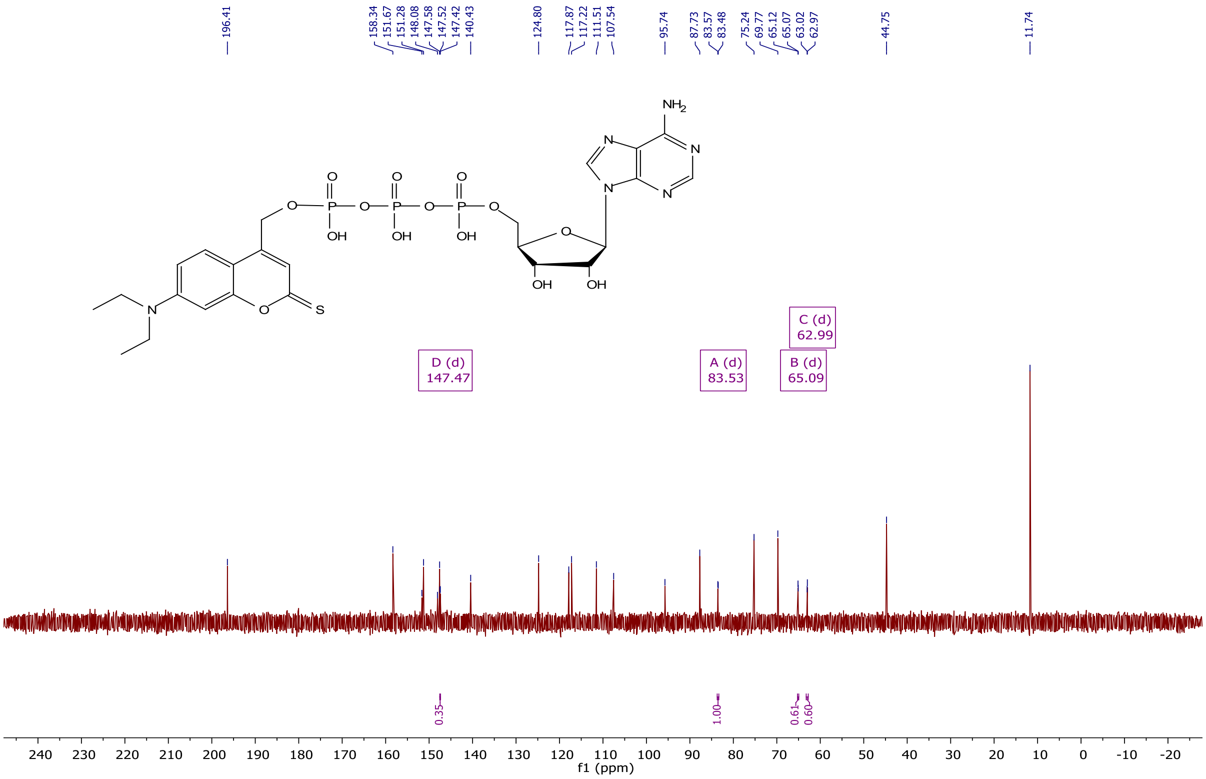

^{13}C NMR spectra of **Thio-DEACM-caged ATP**

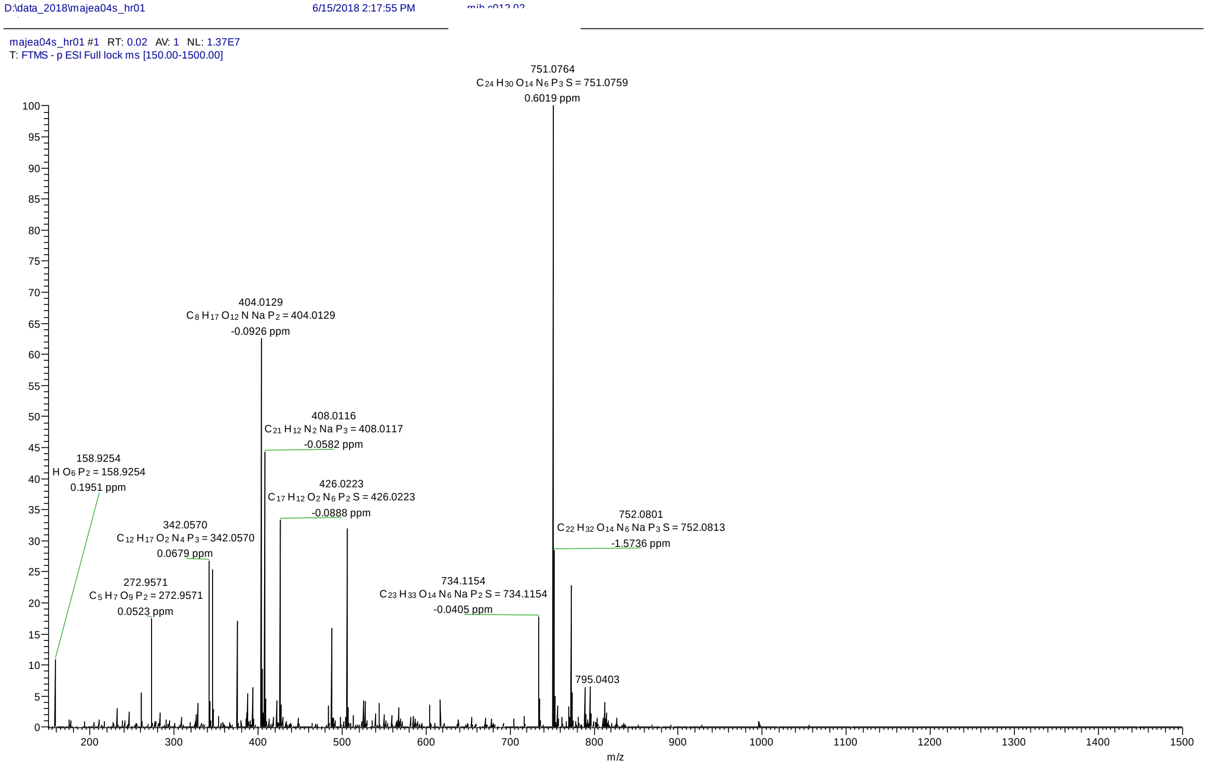

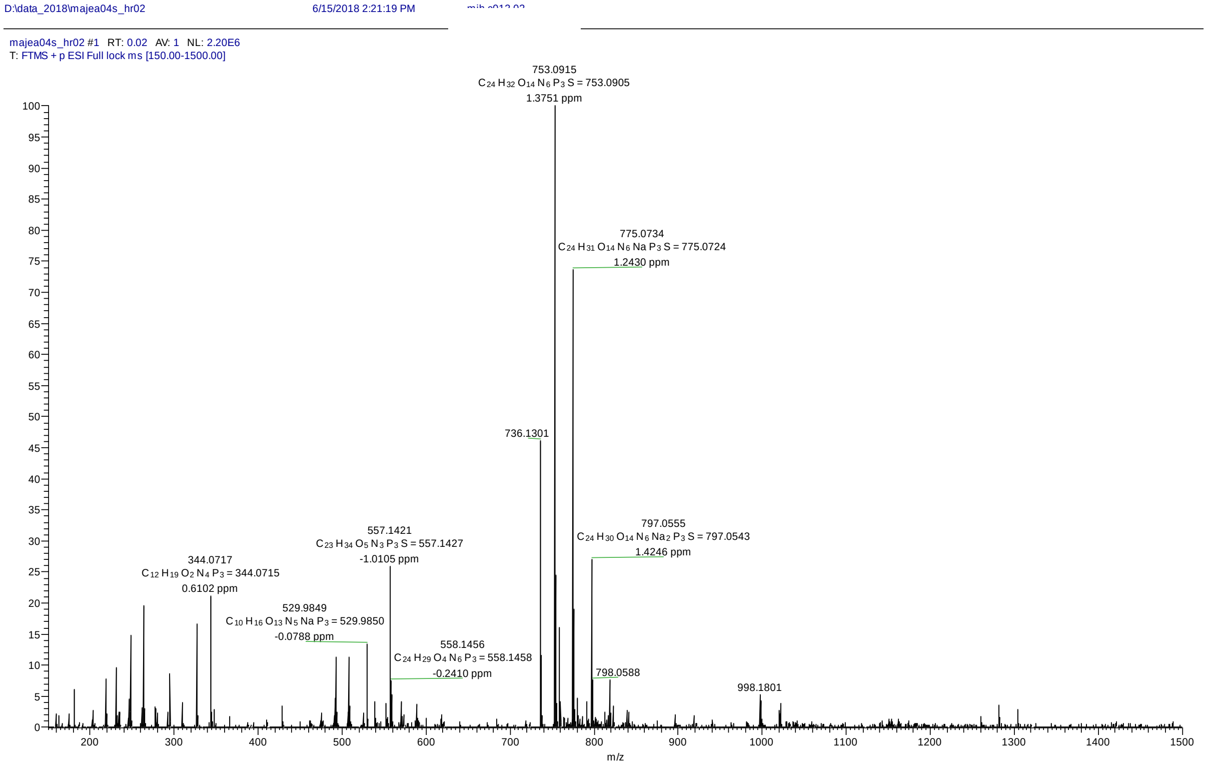

MASS spectra of **Thio-DEACM-caged ATP**

DEACM-AP$_4$

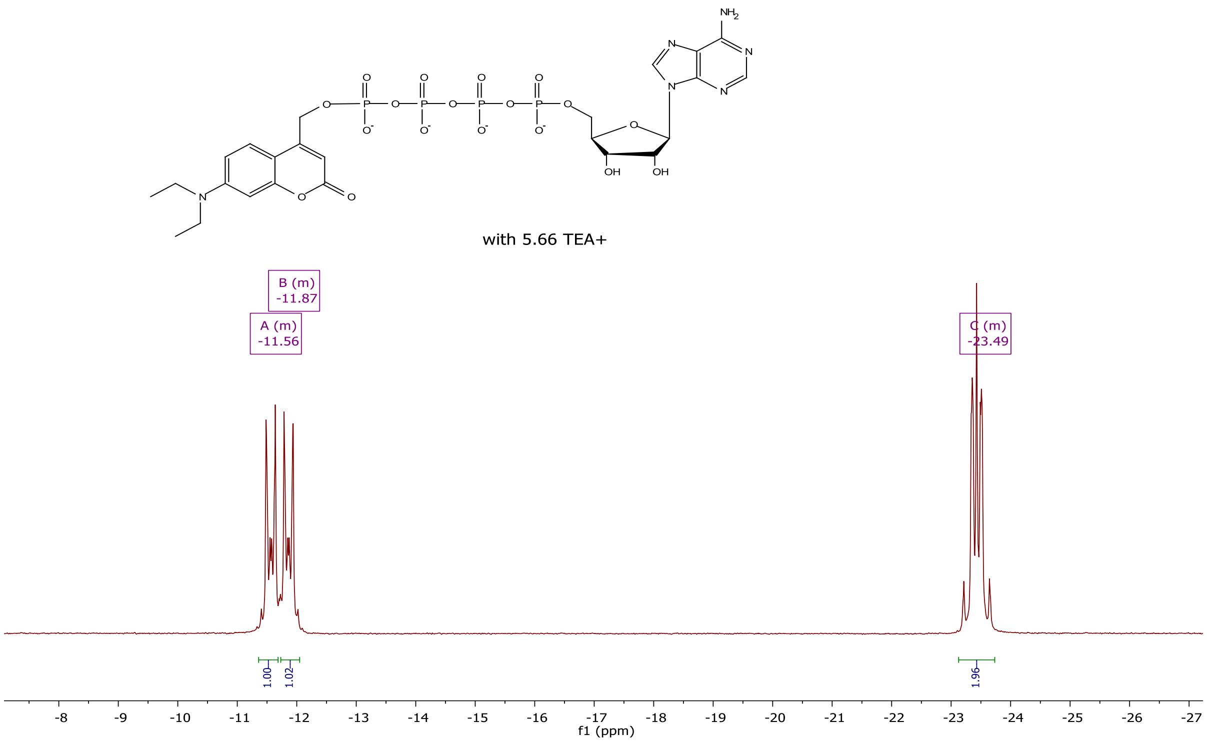

^{31}P NMR spectra of **DEACM-caged AP$_4$**

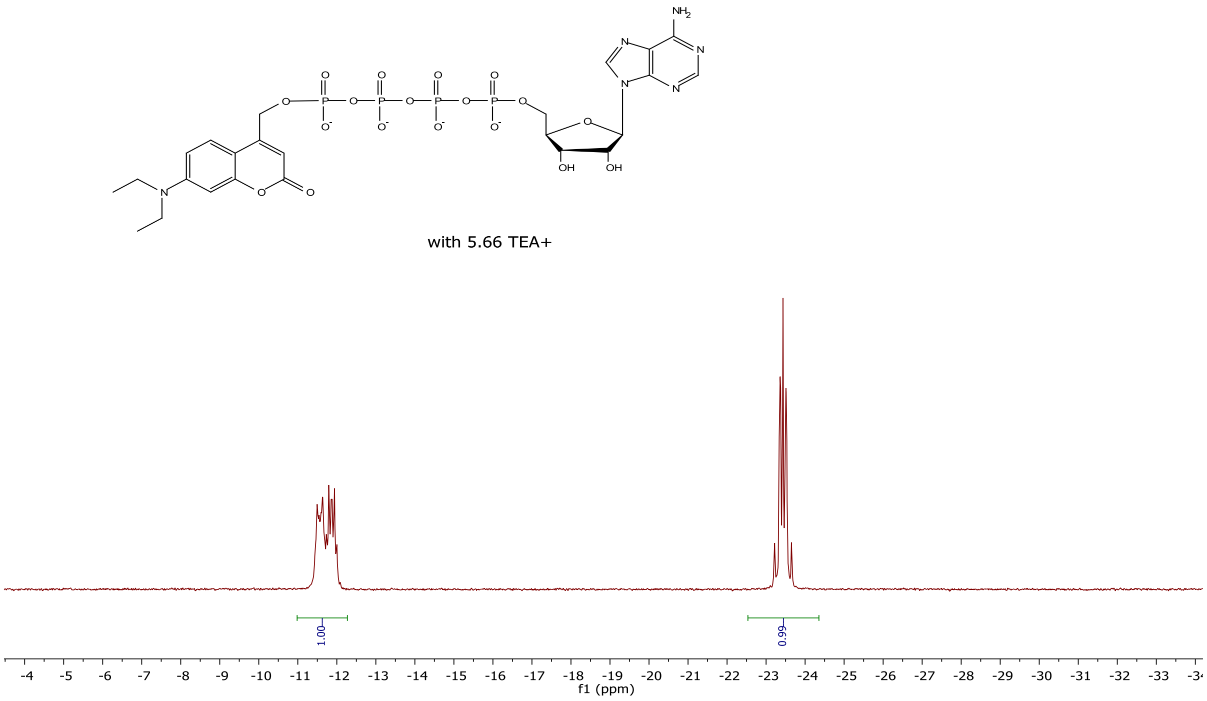

^{31}P coupled NMR spectra of **DEACM-caged AP$_4$**

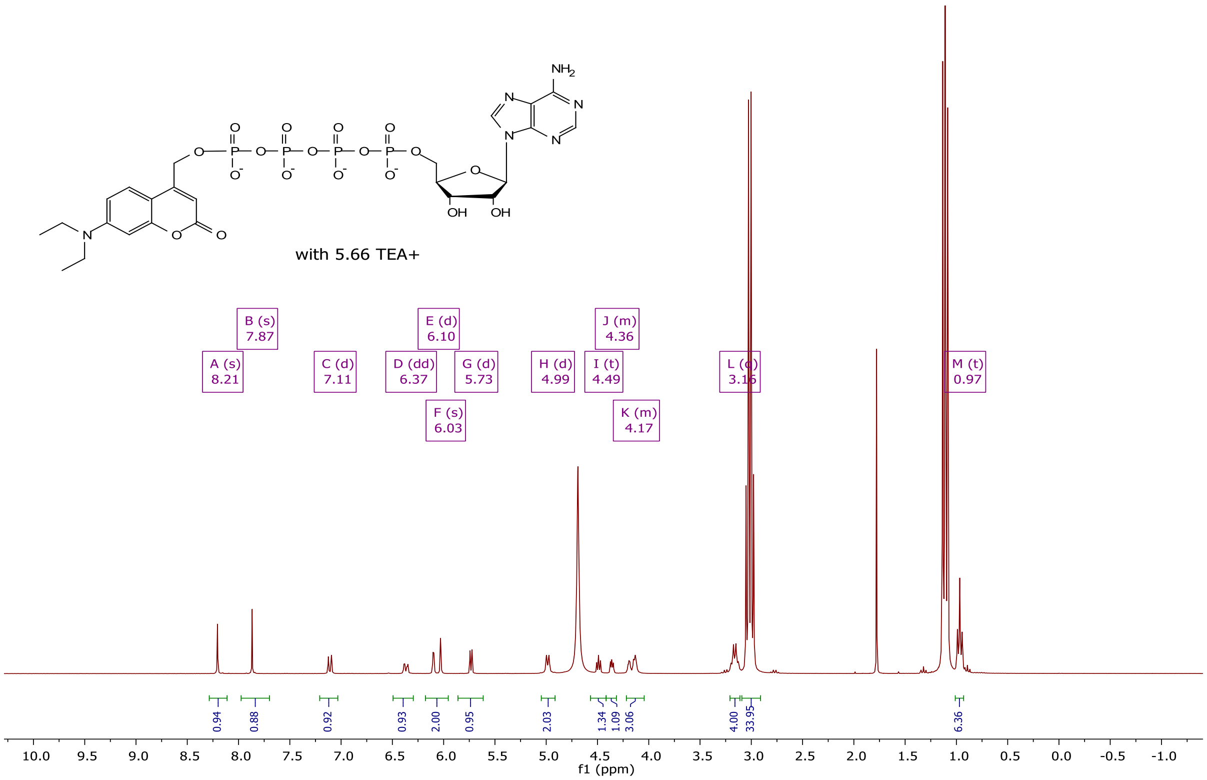

^{1}**H** NMR spectra of **DEACM-caged AP$_4$**

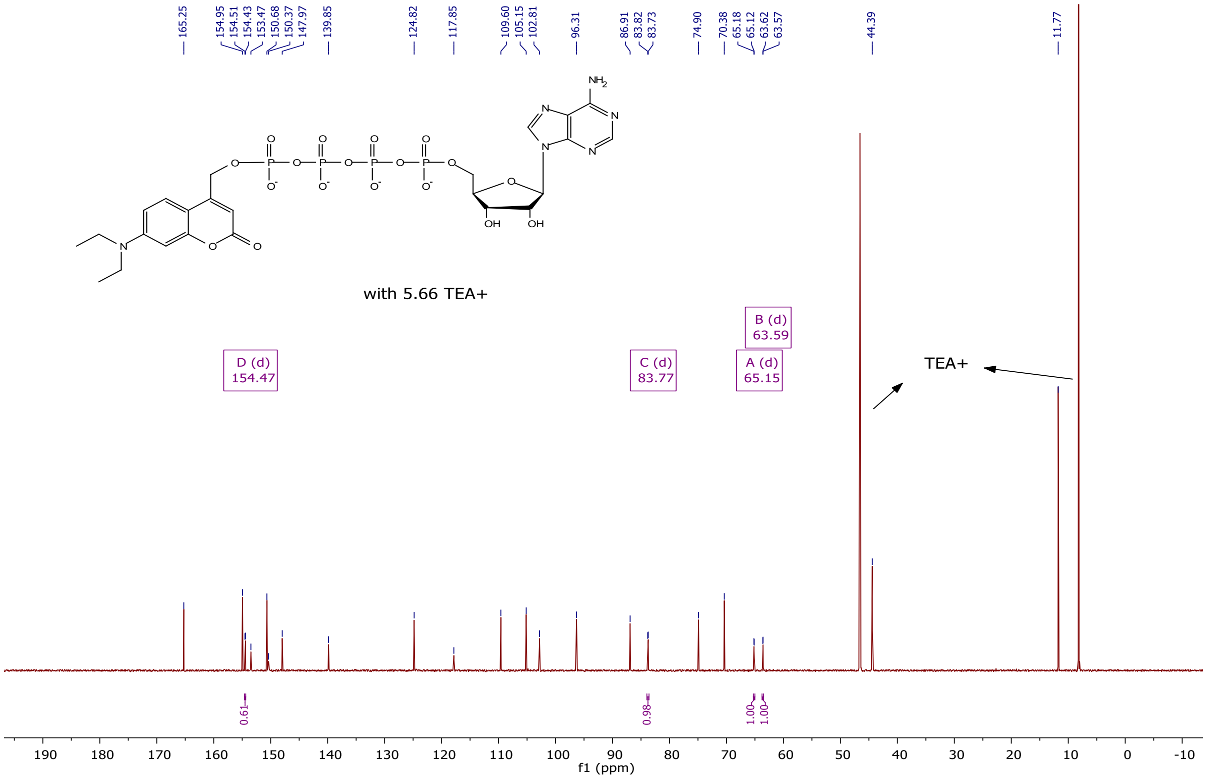

^{13}**C** NMR spectra of **DEACM-caged AP$_4$**

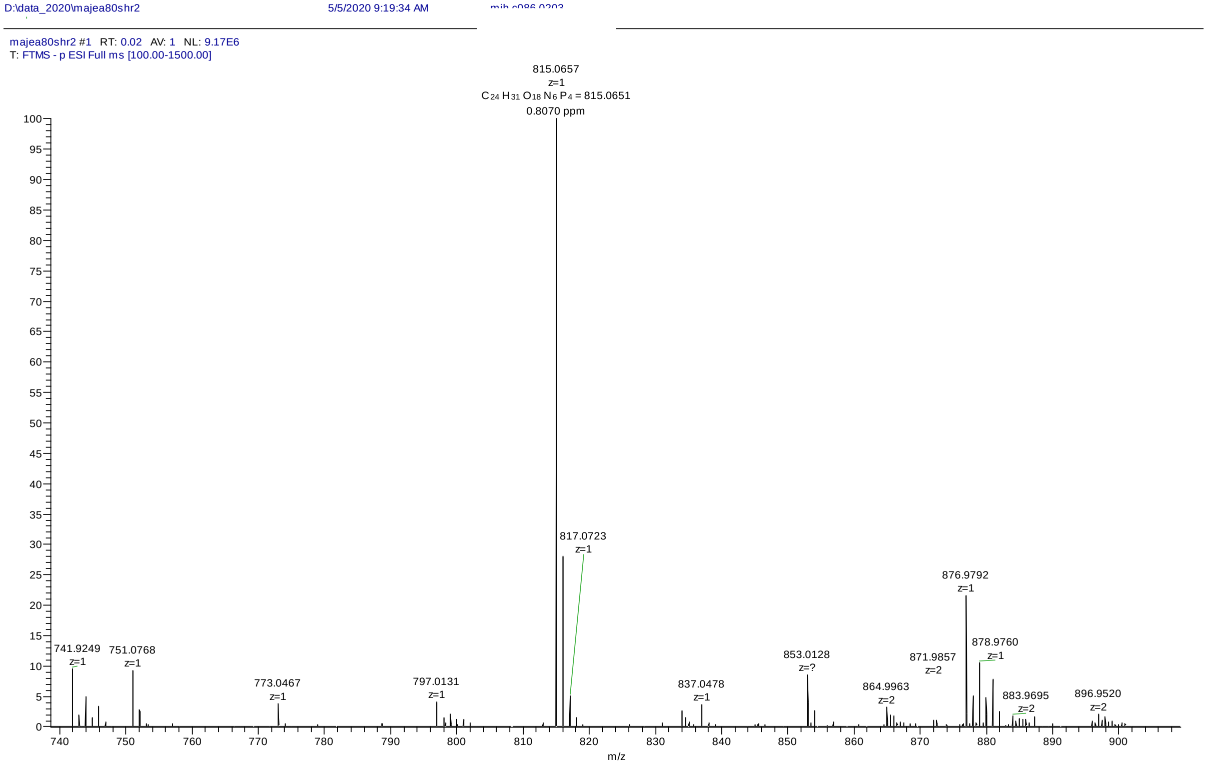

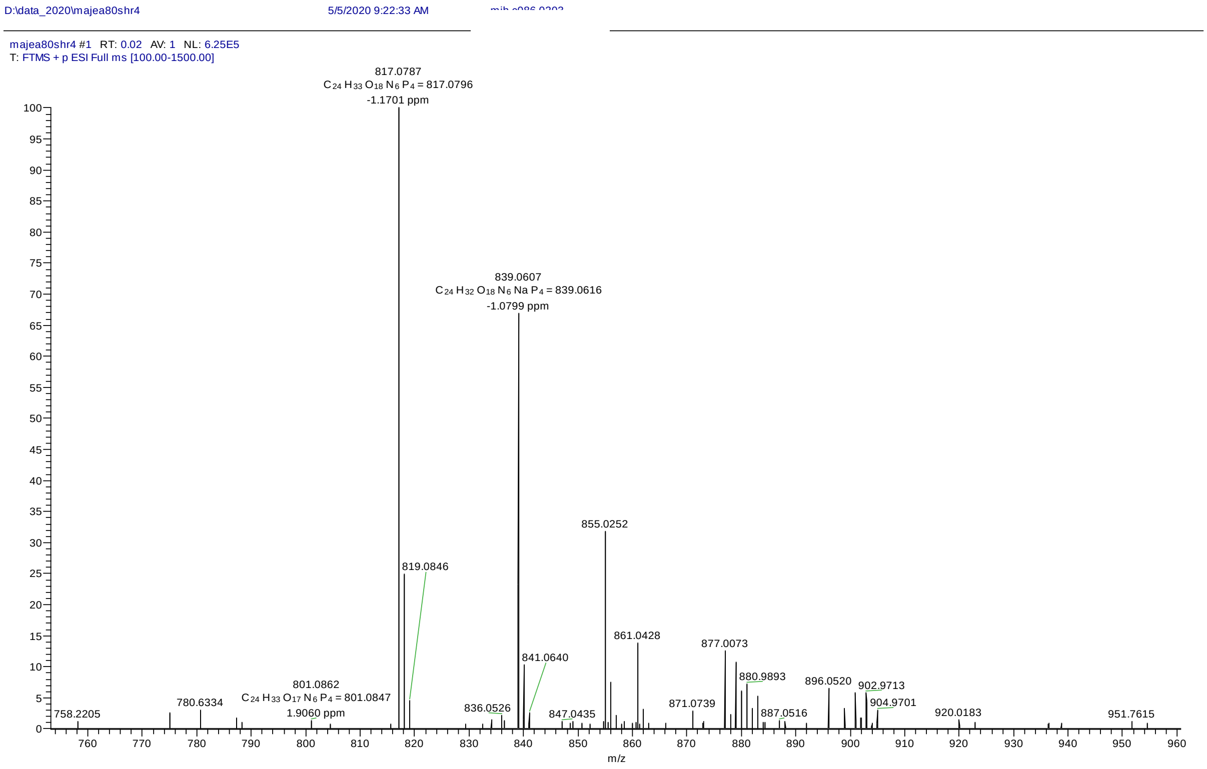

MASS spectra of **DEACM-caged AP$_4$**

Thio-DEACM-AP_4

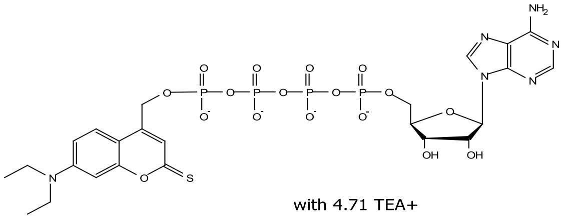

with 4.71 TEA+

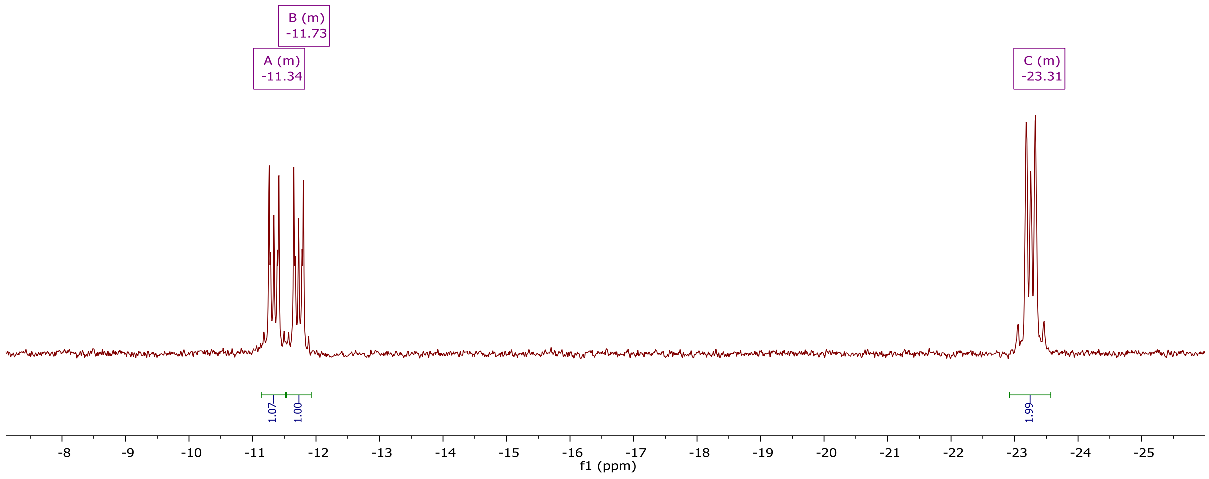

^{31}P NMR spectra of **Thio-DEACM-caged AP_4**

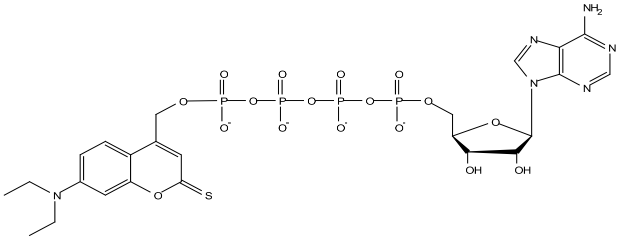

with 4.71 TEA+

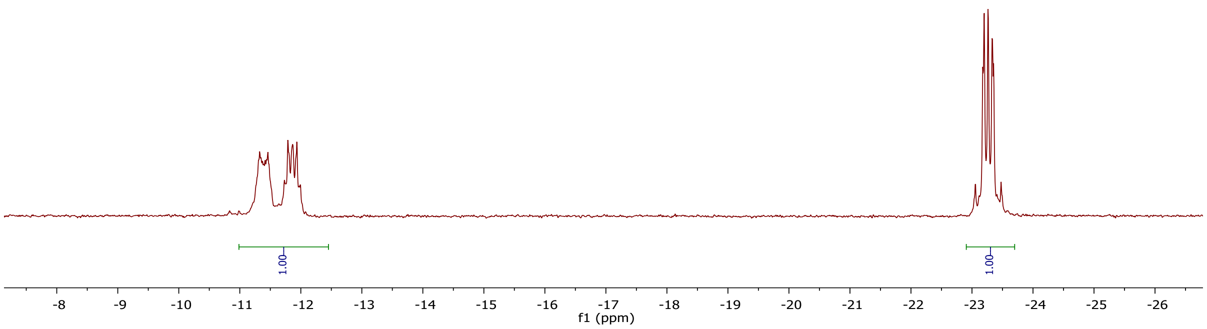

^{31}P coupled NMR spectra of **Thio-DEACM-caged AP_4**

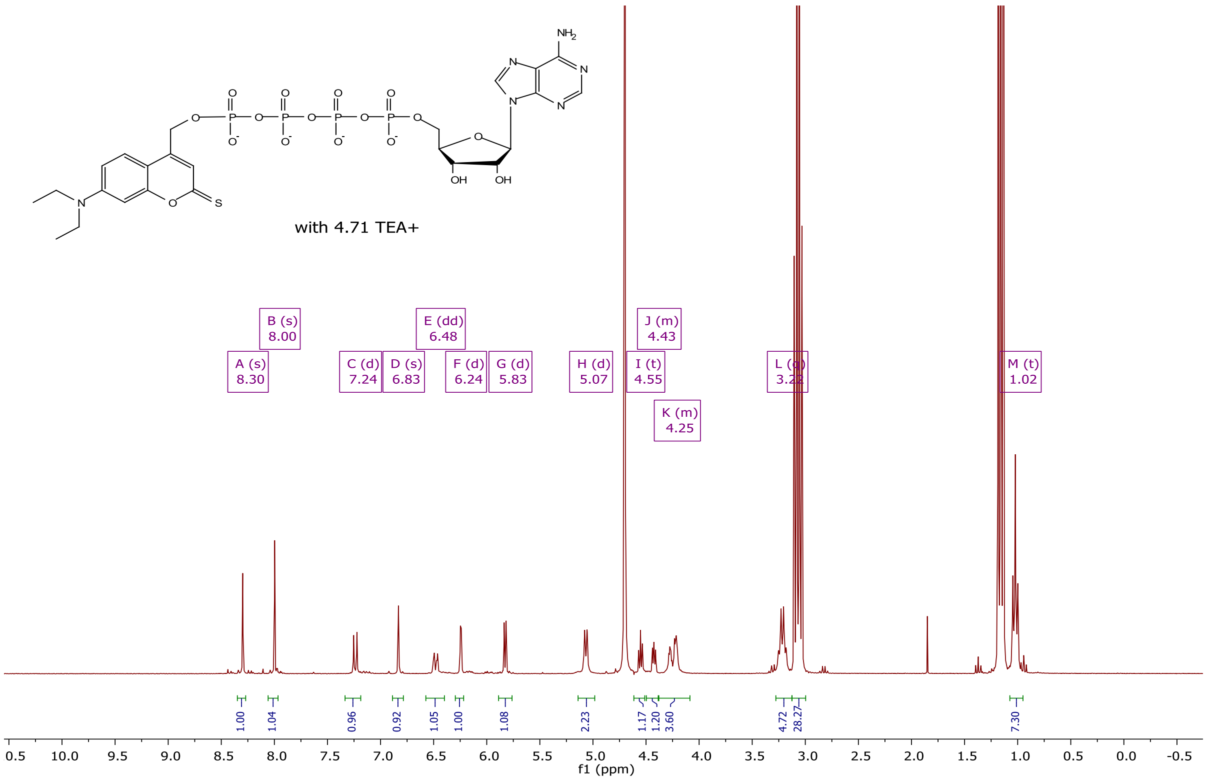

^{1}H NMR spectra of **Thio-DEACM-caged AP_4**

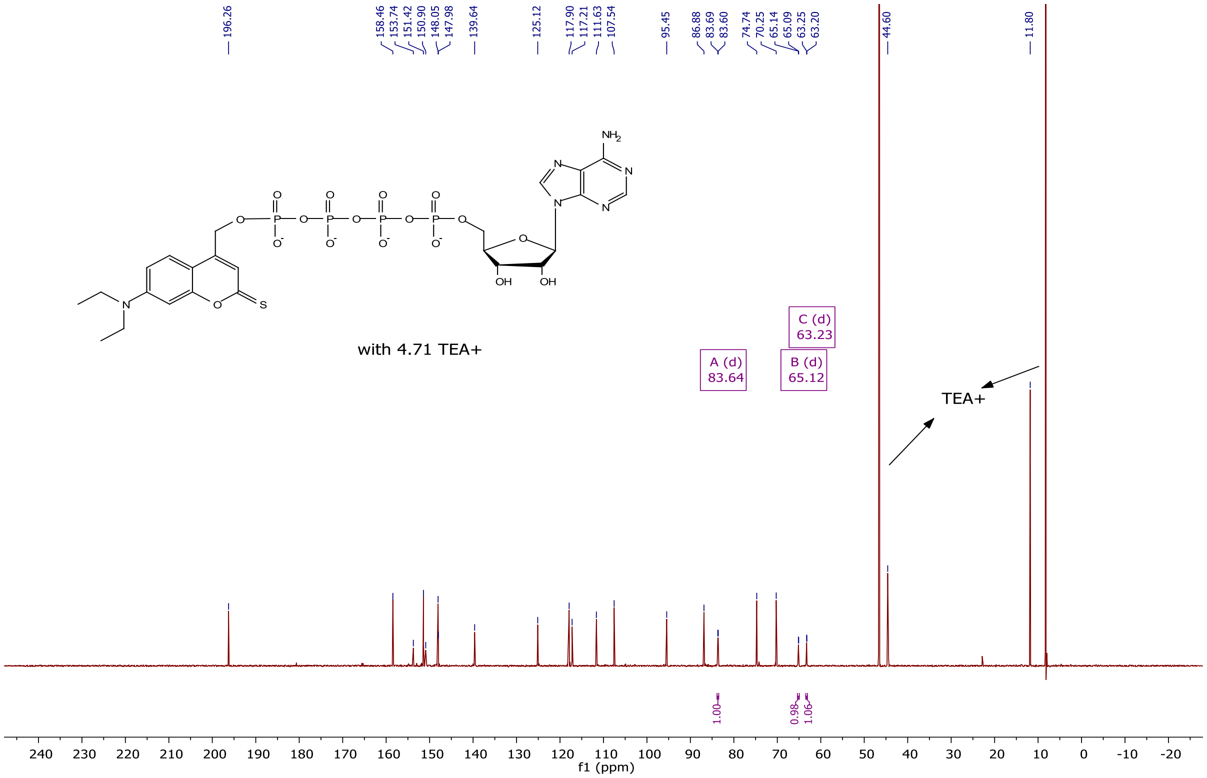

^{13}C NMR spectra of **Thio-DEACM-caged AP_4**

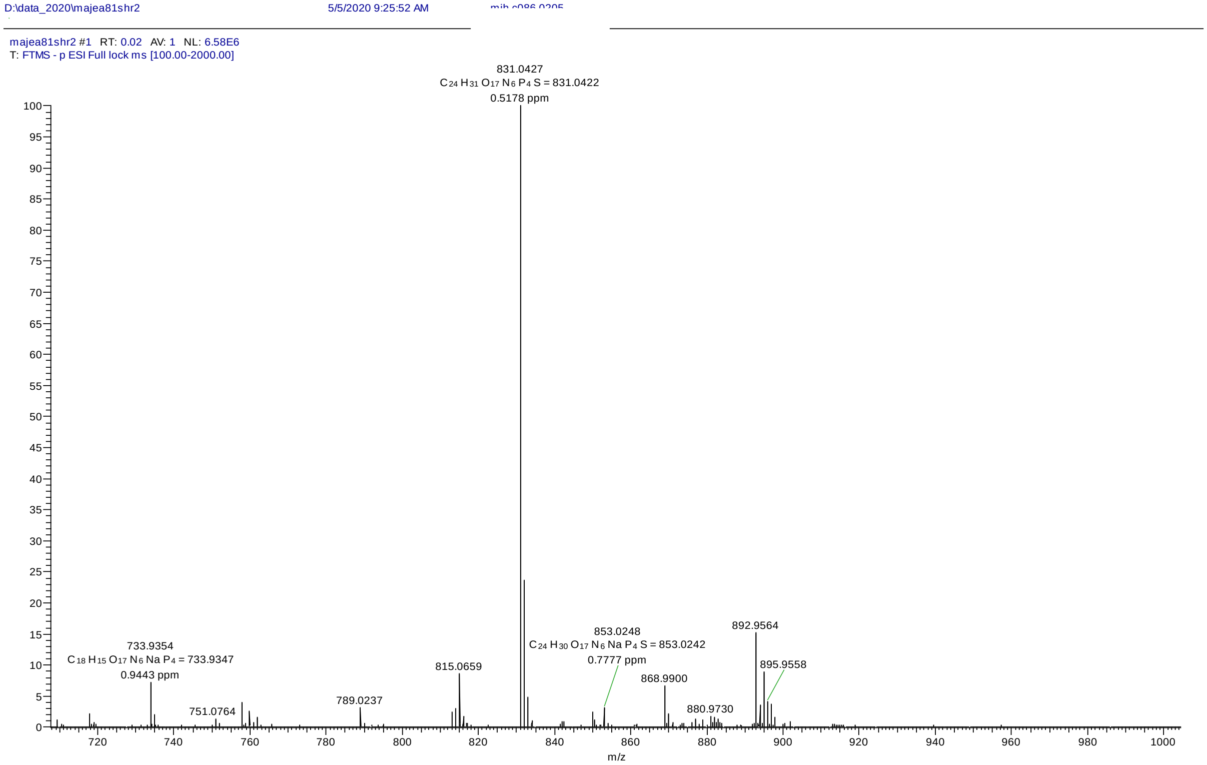

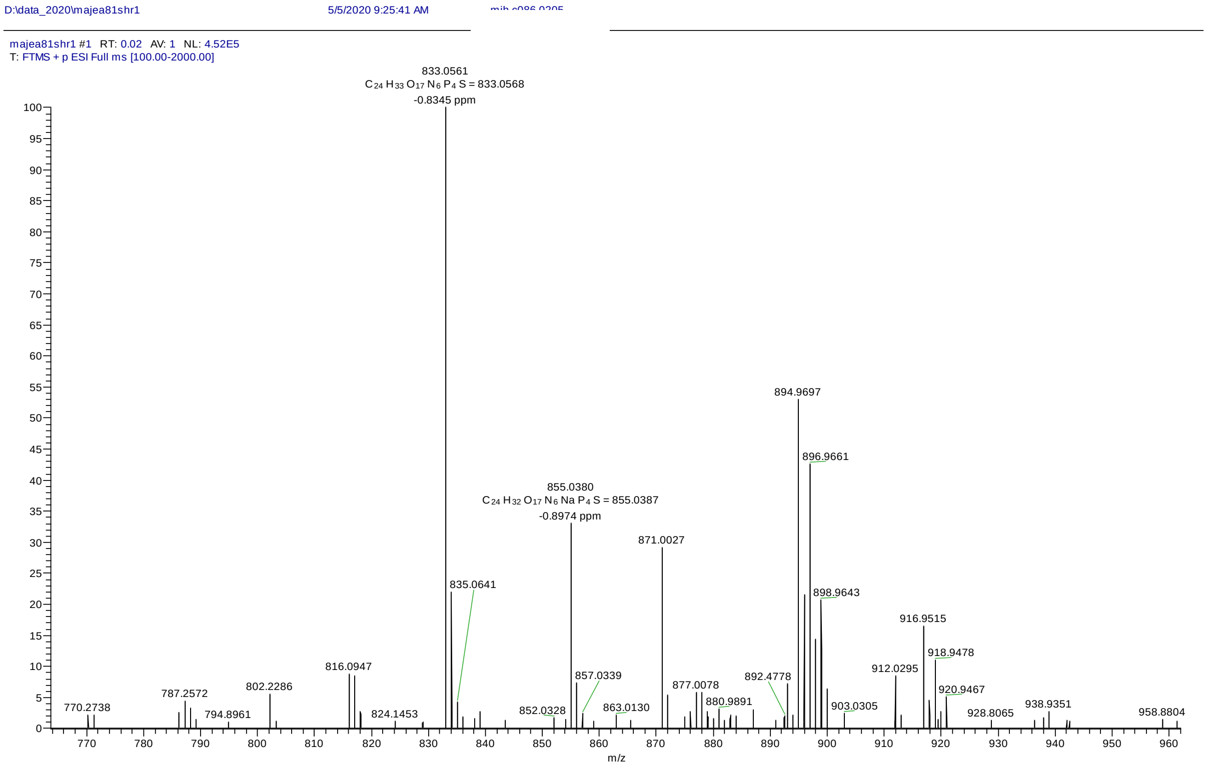

MASS spectra of **Thio-DEACM-caged AP$_4$**

7. Reference

1. Weinrich, T.; Gränz, M.; Grünewald, C.; Prisner, T.F.; Göbel, M.W. Synthesis of a Cytidine Phosphoramidite with Protected Nitroxide Spin Label for EPR Experiments with RNA. *European J. Org. Chem.* **2017**, *2017*, 491–496, doi:10.1002/ejoc.201601174.
2. Fournier, L.; Gauron, C.; Xu, L.; Aujard, I.; Le Saux, T.; Gagey-Eilstein, N.; Maurin, S.; Dubruille, S.; Baudin, J.B.; Bensimon, D.; et al. A blue-absorbing photolabile protecting group for in vivo chromatically orthogonal photoactivation. *ACS Chem. Biol.* **2013**, *8*, 1528–1536, doi:10.1021/cb400178m.
3. Hofer, A.; Cremosnik, G.S.; Müller, A.C.; Giambruno, R.; Trefzer, C.; Superti-Furga, G.; Bennett, K.L.; Jessen, H.J. A Modular Synthesis of Modified Phosphoanhydrides. *Chem. - A Eur. J.* **2015**, *21*, 10116–10122, doi:10.1002/chem.201500838.
4. Becke, A.D. Density-functional thermochemistry. III. The role of exact exchange. *J. Chem. Phys.* **1993**, *98*, 5648–5652, doi:10.1063/1.464913.
5. Stephens, P.J.; Devlin, F.J.; Chabalowski, C.F.; Frisch, M.J. Ab Initio calculation of vibrational absorption and circular dichroism spectra using density functional force fields. *J. Phys. Chem.* **1994**, *98*, 11623–11627, doi:10.1021/j100096a001.
6. Weigend, F.; Ahlrichs, R. Balanced basis sets of split valence, triple zeta valence and quadruple zeta valence quality for H to Rn: Design and assessment of accuracy. *Phys. Chem. Chem. Phys.* **2005**, *7*, 3297–3305, doi:10.1039/b508541a.
7. Weigend, F. Accurate Coulomb-fitting basis sets for H to Rn. *Phys. Chem. Chem. Phys.* **2006**, *8*, 1057–1065, doi:10.1039/b515623h.
8. Miertuš, S.; Scrocco, E.; Tomasi, J. Electrostatic interaction of a solute with a continuum. A direct utilizaion of AB initio molecular potentials for the prevision of solvent effects. *Chem. Phys.* **1981**, *55*, 117–129, doi:10.1016/0301-0104(81)85090-2.
9. Gaussian 16, Revision C.01, M. J. Frisch, G. W. Trucks, H. B. Schlegel, G. E. Scuseria, M. A. Robb, J. R. Cheeseman, G. Scalmani, V. Barone, G. A. Petersson, H. Nakatsuji, X. Li, M. Caricato, A. V. Marenich, J. Bloino, B. G. Janesko, R. Gomperts, B. Mennucci, H. P. Hratchian, J. V. Ortiz, A. F. Izmaylov, J. L. Sonnenberg, D. Williams-Young, F. Ding, F. Lipparini, F. Egidi, J. Goings, B. Peng, A. Petrone, T. Henderson, D. Ranasinghe, V. G. Zakrzewski, J. Gao, N. Rega, G. Zheng, W. Liang, M. Hada, M. Ehara, K. Toyota, R. Fukuda, J. Hasegawa, M. Ishida, T. Nakajima, Y. Honda, O. Kitao, H. Nakai, T. Vreven, K. Throssell, J. A. Montgomery, Jr., J. E. Peralta, F. Ogliaro, M. J. Bearpark, J. J. Heyd, E. N. Brothers, K. N. Kudin, V. N. Staroverov, T. A. Keith, R. Kobayashi, J. Normand, K. Raghavachari, A. P. Rendell, J. C. Burant, S. S. Iyengar, J. Tomasi, M. Cossi, J. M. Millam, M. Klene, C. Adamo, R. Cammi, J. W. Ochterski, R. L. Martin, K. Morokuma, O. Farkas, J. B. Foresman, and D. J. Fox, Gaussian, Inc., Wallingford CT, 2019.
10. T. Bruhn, A. Schaumlöffel, Y. Hemberger, G. Pescitelli, SpecDis, Version 1.70, Berlin, Germany, **2017**.
